# Measurement of lepton differential distributions and the top quark mass in $t\bar{t}$ production in *pp* collisions at $\sqrt{s}=8$ TeV with the ATLAS detector

**DOI:** 10.1140/epjc/s10052-017-5349-9

**Published:** 2017-11-25

**Authors:** M. Aaboud, G. Aad, B. Abbott, O. Abdinov, B. Abeloos, S. H. Abidi, O. S. AbouZeid, N. L. Abraham, H. Abramowicz, H. Abreu, R. Abreu, Y. Abulaiti, B. S. Acharya, S. Adachi, L. Adamczyk, J. Adelman, M. Adersberger, T. Adye, A. A. Affolder, Y. Afik, T. Agatonovic-Jovin, C. Agheorghiesei, J. A. Aguilar-Saavedra, S. P. Ahlen, F. Ahmadov, G. Aielli, S. Akatsuka, H. Akerstedt, T. P. A. Åkesson, E. Akilli, A. V. Akimov, G. L. Alberghi, J. Albert, P. Albicocco, M. J. Alconada Verzini, S. C. Alderweireldt, M. Aleksa, I. N. Aleksandrov, C. Alexa, G. Alexander, T. Alexopoulos, M. Alhroob, B. Ali, M. Aliev, G. Alimonti, J. Alison, S. P. Alkire, B. M. M. Allbrooke, B. W. Allen, P. P. Allport, A. Aloisio, A. Alonso, F. Alonso, C. Alpigiani, A. A. Alshehri, M. I. Alstaty, B. Alvarez Gonzalez, D. Álvarez Piqueras, M. G. Alviggi, B. T. Amadio, Y. Amaral Coutinho, C. Amelung, D. Amidei, S. P. Amor Dos Santos, S. Amoroso, G. Amundsen, C. Anastopoulos, L. S. Ancu, N. Andari, T. Andeen, C. F. Anders, J. K. Anders, K. J. Anderson, A. Andreazza, V. Andrei, S. Angelidakis, I. Angelozzi, A. Angerami, A. V. Anisenkov, N. Anjos, A. Annovi, C. Antel, M. Antonelli, A. Antonov, D. J. Antrim, F. Anulli, M. Aoki, L. Aperio Bella, G. Arabidze, Y. Arai, J. P. Araque, V. Araujo Ferraz, A. T. H. Arce, R. E. Ardell, F. A. Arduh, J-F. Arguin, S. Argyropoulos, M. Arik, A. J. Armbruster, L. J. Armitage, O. Arnaez, H. Arnold, M. Arratia, O. Arslan, A. Artamonov, G. Artoni, S. Artz, S. Asai, N. Asbah, A. Ashkenazi, L. Asquith, K. Assamagan, R. Astalos, M. Atkinson, N. B. Atlay, K. Augsten, G. Avolio, B. Axen, M. K. Ayoub, G. Azuelos, A. E. Baas, M. J. Baca, H. Bachacou, K. Bachas, M. Backes, P. Bagnaia, M. Bahmani, H. Bahrasemani, J. T. Baines, M. Bajic, O. K. Baker, P. J. Bakker, E. M. Baldin, P. Balek, F. Balli, W. K. Balunas, E. Banas, A. Bandyopadhyay, Sw. Banerjee, A. A. E. Bannoura, L. Barak, E. L. Barberio, D. Barberis, M. Barbero, T. Barillari, M-S Barisits, J. T. Barkeloo, T. Barklow, N. Barlow, S. L. Barnes, B. M. Barnett, R. M. Barnett, Z. Barnovska-Blenessy, A. Baroncelli, G. Barone, A. J. Barr, L. Barranco Navarro, F. Barreiro, J. Barreiro Guimarães da Costa, R. Bartoldus, A. E. Barton, P. Bartos, A. Basalaev, A. Bassalat, R. L. Bates, S. J. Batista, J. R. Batley, M. Battaglia, M. Bauce, F. Bauer, H. S. Bawa, J. B. Beacham, M. D. Beattie, T. Beau, P. H. Beauchemin, P. Bechtle, H. P. Beck, H. C. Beck, K. Becker, M. Becker, C. Becot, A. J. Beddall, A. Beddall, V. A. Bednyakov, M. Bedognetti, C. P. Bee, T. A. Beermann, M. Begalli, M. Begel, J. K. Behr, A. S. Bell, G. Bella, L. Bellagamba, A. Bellerive, M. Bellomo, K. Belotskiy, O. Beltramello, N. L. Belyaev, O. Benary, D. Benchekroun, M. Bender, N. Benekos, Y. Benhammou, E. Benhar Noccioli, J. Benitez, D. P. Benjamin, M. Benoit, J. R. Bensinger, S. Bentvelsen, L. Beresford, M. Beretta, D. Berge, E. Bergeaas Kuutmann, N. Berger, J. Beringer, S. Berlendis, N. R. Bernard, G. Bernardi, C. Bernius, F. U. Bernlochner, T. Berry, P. Berta, C. Bertella, G. Bertoli, I. A. Bertram, C. Bertsche, D. Bertsche, G. J. Besjes, O. Bessidskaia Bylund, M. Bessner, N. Besson, A. Bethani, S. Bethke, A. Betti, A. J. Bevan, J. Beyer, R. M. Bianchi, O. Biebel, D. Biedermann, R. Bielski, K. Bierwagen, N. V. Biesuz, M. Biglietti, T. R. V. Billoud, H. Bilokon, M. Bindi, A. Bingul, C. Bini, S. Biondi, T. Bisanz, C. Bittrich, D. M. Bjergaard, J. E. Black, K. M. Black, R. E. Blair, T. Blazek, I. Bloch, C. Blocker, A. Blue, U. Blumenschein, S. Blunier, G. J. Bobbink, V. S. Bobrovnikov, S. S. Bocchetta, A. Bocci, C. Bock, M. Boehler, D. Boerner, D. Bogavac, A. G. Bogdanchikov, C. Bohm, V. Boisvert, P. Bokan, T. Bold, A. S. Boldyrev, A. E. Bolz, M. Bomben, M. Bona, M. Boonekamp, A. Borisov, G. Borissov, J. Bortfeldt, D. Bortoletto, V. Bortolotto, D. Boscherini, M. Bosman, J. D. Bossio Sola, J. Boudreau, E. V. Bouhova-Thacker, D. Boumediene, C. Bourdarios, S. K. Boutle, A. Boveia, J. Boyd, I. R. Boyko, A. J. Bozson, J. Bracinik, A. Brandt, G. Brandt, O. Brandt, F. Braren, U. Bratzler, B. Brau, J. E. Brau, W. D. Breaden Madden, K. Brendlinger, A. J. Brennan, L. Brenner, R. Brenner, S. Bressler, D. L. Briglin, T. M. Bristow, D. Britton, D. Britzger, F. M. Brochu, I. Brock, R. Brock, G. Brooijmans, T. Brooks, W. K. Brooks, J. Brosamer, E. Brost, J. H Broughton, P. A. Bruckman de Renstrom, D. Bruncko, A. Bruni, G. Bruni, L. S. Bruni, S. Bruno, BH Brunt, M. Bruschi, N. Bruscino, P. Bryant, L. Bryngemark, T. Buanes, Q. Buat, P. Buchholz, A. G. Buckley, I. A. Budagov, F. Buehrer, M. K. Bugge, O. Bulekov, D. Bullock, T. J. Burch, S. Burdin, C. D. Burgard, A. M. Burger, B. Burghgrave, K. Burka, S. Burke, I. Burmeister, J. T. P. Burr, D. Büscher, V. Büscher, P. Bussey, J. M. Butler, C. M. Buttar, J. M. Butterworth, P. Butti, W. Buttinger, A. Buzatu, A. R. Buzykaev, S. Cabrera Urbán, D. Caforio, H. Cai, V. M. Cairo, O. Cakir, N. Calace, P. Calafiura, A. Calandri, G. Calderini, P. Calfayan, G. Callea, L. P. Caloba, S. Calvente Lopez, D. Calvet, S. Calvet, T. P. Calvet, R. Camacho Toro, S. Camarda, P. Camarri, D. Cameron, R. Caminal Armadans, C. Camincher, S. Campana, M. Campanelli, A. Camplani, A. Campoverde, V. Canale, M. Cano Bret, J. Cantero, T. Cao, M. D. M. Capeans Garrido, I. Caprini, M. Caprini, M. Capua, R. M. Carbone, R. Cardarelli, F. Cardillo, I. Carli, T. Carli, G. Carlino, B. T. Carlson, L. Carminati, R. M. D. Carney, S. Caron, E. Carquin, S. Carrá, G. D. Carrillo-Montoya, D. Casadei, M. P. Casado, A. F. Casha, M. Casolino, D. W. Casper, R. Castelijn, V. Castillo Gimenez, N. F. Castro, A. Catinaccio, J. R. Catmore, A. Cattai, J. Caudron, V. Cavaliere, E. Cavallaro, D. Cavalli, M. Cavalli-Sforza, V. Cavasinni, E. Celebi, F. Ceradini, L. Cerda Alberich, A. S. Cerqueira, A. Cerri, L. Cerrito, F. Cerutti, A. Cervelli, S. A. Cetin, A. Chafaq, D. Chakraborty, S. K. Chan, W. S. Chan, Y. L. Chan, P. Chang, J. D. Chapman, D. G. Charlton, C. C. Chau, C. A. Chavez Barajas, S. Che, S. Cheatham, A. Chegwidden, S. Chekanov, S. V. Chekulaev, G. A. Chelkov, M. A. Chelstowska, C. Chen, C. Chen, H. Chen, J. Chen, S. Chen, S. Chen, X. Chen, Y. Chen, H. C. Cheng, H. J. Cheng, A. Cheplakov, E. Cheremushkina, R. Cherkaoui El Moursli, E. Cheu, K. Cheung, L. Chevalier, V. Chiarella, G. Chiarelli, G. Chiodini, A. S. Chisholm, A. Chitan, Y. H. Chiu, M. V. Chizhov, K. Choi, A. R. Chomont, S. Chouridou, Y. S. Chow, V. Christodoulou, M. C. Chu, J. Chudoba, A. J. Chuinard, J. J. Chwastowski, L. Chytka, A. K. Ciftci, D. Cinca, V. Cindro, I. A. Cioara, A. Ciocio, F. Cirotto, Z. H. Citron, M. Citterio, M. Ciubancan, A. Clark, B. L. Clark, M. R. Clark, P. J. Clark, R. N. Clarke, C. Clement, Y. Coadou, M. Cobal, A. Coccaro, J. Cochran, L. Colasurdo, B. Cole, A. P. Colijn, J. Collot, T. Colombo, P. Conde Muiño, E. Coniavitis, S. H. Connell, I. A. Connelly, S. Constantinescu, G. Conti, F. Conventi, M. Cooke, A. M. Cooper-Sarkar, F. Cormier, K. J. R. Cormier, M. Corradi, F. Corriveau, A. Cortes-Gonzalez, G. Costa, M. J. Costa, D. Costanzo, G. Cottin, G. Cowan, B. E. Cox, K. Cranmer, S. J. Crawley, R. A. Creager, G. Cree, S. Crépé-Renaudin, F. Crescioli, W. A. Cribbs, M. Cristinziani, V. Croft, G. Crosetti, A. Cueto, T. Cuhadar Donszelmann, A. R. Cukierman, J. Cummings, M. Curatolo, J. Cúth, S. Czekierda, P. Czodrowski, G. D’amen, S. D’Auria, L. D’eramo, M. D’Onofrio, M. J. Da Cunha Sargedas De Sousa, C. Da Via, W. Dabrowski, T. Dado, T. Dai, O. Dale, F. Dallaire, C. Dallapiccola, M. Dam, J. R. Dandoy, M. F. Daneri, N. P. Dang, A. C. Daniells, N. S. Dann, M. Danninger, M. Dano Hoffmann, V. Dao, G. Darbo, S. Darmora, J. Dassoulas, A. Dattagupta, T. Daubney, W. Davey, C. David, T. Davidek, D. R. Davis, P. Davison, E. Dawe, I. Dawson, K. De, R. de Asmundis, A. De Benedetti, S. De Castro, S. De Cecco, N. De Groot, P. de Jong, H. De la Torre, F. De Lorenzi, A. De Maria, D. De Pedis, A. De Salvo, U. De Sanctis, A. De Santo, K. De Vasconcelos Corga, J. B. De Vivie De Regie, R. Debbe, C. Debenedetti, D. V. Dedovich, N. Dehghanian, I. Deigaard, M. Del Gaudio, J. Del Peso, D. Delgove, F. Deliot, C. M. Delitzsch, A. Dell’Acqua, L. Dell’Asta, M. Dell’Orso, M. Della Pietra, D. della Volpe, M. Delmastro, C. Delporte, P. A. Delsart, D. A. DeMarco, S. Demers, M. Demichev, A. Demilly, S. P. Denisov, D. Denysiuk, D. Derendarz, J. E. Derkaoui, F. Derue, P. Dervan, K. Desch, C. Deterre, K. Dette, M. R. Devesa, P. O. Deviveiros, A. Dewhurst, S. Dhaliwal, F. A. Di Bello, A. Di Ciaccio, L. Di Ciaccio, W. K. Di Clemente, C. Di Donato, A. Di Girolamo, B. Di Girolamo, B. Di Micco, R. Di Nardo, K. F. Di Petrillo, A. Di Simone, R. Di Sipio, D. Di Valentino, C. Diaconu, M. Diamond, F. A. Dias, M. A. Diaz, E. B. Diehl, J. Dietrich, S. Díez Cornell, A. Dimitrievska, J. Dingfelder, P. Dita, S. Dita, F. Dittus, F. Djama, T. Djobava, J. I. Djuvsland, M. A. B. do Vale, D. Dobos, M. Dobre, D. Dodsworth, C. Doglioni, J. Dolejsi, Z. Dolezal, M. Donadelli, S. Donati, P. Dondero, J. Donini, J. Dopke, A. Doria, M. T. Dova, A. T. Doyle, E. Drechsler, M. Dris, Y. Du, J. Duarte-Campderros, F. Dubinin, A. Dubreuil, E. Duchovni, G. Duckeck, A. Ducourthial, O. A. Ducu, D. Duda, A. Dudarev, A. Chr. Dudder, E. M. Duffield, L. Duflot, M. Dührssen, C. Dulsen, M. Dumancic, A. E. Dumitriu, A. K. Duncan, M. Dunford, A. Duperrin, H. Duran Yildiz, M. Düren, A. Durglishvili, D. Duschinger, B. Dutta, D. Duvnjak, M. Dyndal, B. S. Dziedzic, C. Eckardt, K. M. Ecker, R. C. Edgar, T. Eifert, G. Eigen, K. Einsweiler, T. Ekelof, M. El Kacimi, R. El Kosseifi, V. Ellajosyula, M. Ellert, S. Elles, F. Ellinghaus, A. A. Elliot, N. Ellis, J. Elmsheuser, M. Elsing, D. Emeliyanov, Y. Enari, J. S. Ennis, M. B. Epland, J. Erdmann, A. Ereditato, M. Ernst, S. Errede, M. Escalier, C. Escobar, B. Esposito, O. Estrada Pastor, A. I. Etienvre, E. Etzion, H. Evans, A. Ezhilov, M. Ezzi, F. Fabbri, L. Fabbri, V. Fabiani, G. Facini, R. M. Fakhrutdinov, S. Falciano, R. J. Falla, J. Faltova, Y. Fang, M. Fanti, A. Farbin, A. Farilla, C. Farina, E. M. Farina, T. Farooque, S. Farrell, S. M. Farrington, P. Farthouat, F. Fassi, P. Fassnacht, D. Fassouliotis, M. Faucci Giannelli, A. Favareto, W. J. Fawcett, L. Fayard, O. L. Fedin, W. Fedorko, S. Feigl, L. Feligioni, C. Feng, E. J. Feng, M. J. Fenton, A. B. Fenyuk, L. Feremenga, P. Fernandez Martinez, J. Ferrando, A. Ferrari, P. Ferrari, R. Ferrari, D. E. Ferreira de Lima, A. Ferrer, D. Ferrere, C. Ferretti, F. Fiedler, A. Filipčič, M. Filipuzzi, F. Filthaut, M. Fincke-Keeler, K. D. Finelli, M. C. N. Fiolhais, L. Fiorini, A. Fischer, C. Fischer, J. Fischer, W. C. Fisher, N. Flaschel, I. Fleck, P. Fleischmann, R. R. M. Fletcher, T. Flick, B. M. Flierl, L. R. Flores Castillo, M. J. Flowerdew, G. T. Forcolin, A. Formica, F. A. Förster, A. Forti, A. G. Foster, D. Fournier, H. Fox, S. Fracchia, P. Francavilla, M. Franchini, S. Franchino, D. Francis, L. Franconi, M. Franklin, M. Frate, M. Fraternali, D. Freeborn, S. M. Fressard-Batraneanu, B. Freund, D. Froidevaux, J. A. Frost, C. Fukunaga, T. Fusayasu, J. Fuster, O. Gabizon, A. Gabrielli, A. Gabrielli, G. P. Gach, S. Gadatsch, S. Gadomski, G. Gagliardi, L. G. Gagnon, C. Galea, B. Galhardo, E. J. Gallas, B. J. Gallop, P. Gallus, G. Galster, K. K. Gan, S. Ganguly, Y. Gao, Y. S. Gao, F. M. Garay Walls, C. García, J. E. García Navarro, J. A. García Pascual, M. Garcia-Sciveres, R. W. Gardner, N. Garelli, V. Garonne, A. Gascon Bravo, K. Gasnikova, C. Gatti, A. Gaudiello, G. Gaudio, I. L. Gavrilenko, C. Gay, G. Gaycken, E. N. Gazis, C. N. P. Gee, J. Geisen, M. Geisen, M. P. Geisler, K. Gellerstedt, C. Gemme, M. H. Genest, C. Geng, S. Gentile, C. Gentsos, S. George, D. Gerbaudo, G. Geßner, S. Ghasemi, M. Ghneimat, B. Giacobbe, S. Giagu, N. Giangiacomi, P. Giannetti, S. M. Gibson, M. Gignac, M. Gilchriese, D. Gillberg, G. Gilles, D. M. Gingrich, M. P. Giordani, F. M. Giorgi, P. F. Giraud, P. Giromini, G. Giugliarelli, D. Giugni, F. Giuli, C. Giuliani, M. Giulini, B. K. Gjelsten, S. Gkaitatzis, I. Gkialas, E. L. Gkougkousis, P. Gkountoumis, L. K. Gladilin, C. Glasman, J. Glatzer, P. C. F. Glaysher, A. Glazov, M. Goblirsch-Kolb, J. Godlewski, S. Goldfarb, T. Golling, D. Golubkov, A. Gomes, R. Gonçalo, R. Goncalves Gama, J. Goncalves Pinto Firmino Da Costa, G. Gonella, L. Gonella, A. Gongadze, J. L. Gonski, S. González de la Hoz, S. Gonzalez-Sevilla, L. Goossens, P. A. Gorbounov, H. A. Gordon, I. Gorelov, B. Gorini, E. Gorini, A. Gorišek, A. T. Goshaw, C. Gössling, M. I. Gostkin, C. A. Gottardo, C. R. Goudet, D. Goujdami, A. G. Goussiou, N. Govender, E. Gozani, I. Grabowska-Bold, P. O. J. Gradin, J. Gramling, E. Gramstad, S. Grancagnolo, V. Gratchev, P. M. Gravila, C. Gray, H. M. Gray, Z. D. Greenwood, C. Grefe, K. Gregersen, I. M. Gregor, P. Grenier, K. Grevtsov, J. Griffiths, A. A. Grillo, K. Grimm, S. Grinstein, Ph. Gris, J.-F. Grivaz, S. Groh, E. Gross, J. Grosse-Knetter, G. C. Grossi, Z. J. Grout, A. Grummer, L. Guan, W. Guan, J. Guenther, F. Guescini, D. Guest, O. Gueta, B. Gui, E. Guido, T. Guillemin, S. Guindon, U. Gul, C. Gumpert, J. Guo, W. Guo, Y. Guo, R. Gupta, S. Gurbuz, G. Gustavino, B. J. Gutelman, P. Gutierrez, N. G. Gutierrez Ortiz, C. Gutschow, C. Guyot, M. P. Guzik, C. Gwenlan, C. B. Gwilliam, A. Haas, C. Haber, H. K. Hadavand, N. Haddad, A. Hadef, S. Hageböck, M. Hagihara, H. Hakobyan, M. Haleem, J. Haley, G. Halladjian, G. D. Hallewell, K. Hamacher, P. Hamal, K. Hamano, A. Hamilton, G. N. Hamity, P. G. Hamnett, L. Han, S. Han, K. Hanagaki, K. Hanawa, M. Hance, D. M. Handl, B. Haney, P. Hanke, J. B. Hansen, J. D. Hansen, M. C. Hansen, P. H. Hansen, K. Hara, A. S. Hard, T. Harenberg, F. Hariri, S. Harkusha, P. F. Harrison, N. M. Hartmann, Y. Hasegawa, A. Hasib, S. Hassani, S. Haug, R. Hauser, L. Hauswald, L. B. Havener, M. Havranek, C. M. Hawkes, R. J. Hawkings, D. Hayakawa, D. Hayden, C. P. Hays, J. M. Hays, H. S. Hayward, S. J. Haywood, S. J. Head, T. Heck, V. Hedberg, L. Heelan, S. Heer, K. K. Heidegger, S. Heim, T. Heim, B. Heinemann, J. J. Heinrich, L. Heinrich, C. Heinz, J. Hejbal, L. Helary, A. Held, S. Hellman, C. Helsens, R. C. W. Henderson, Y. Heng, S. Henkelmann, A. M. Henriques Correia, S. Henrot-Versille, G. H. Herbert, H. Herde, V. Herget, Y. Hernández Jiménez, H. Herr, G. Herten, R. Hertenberger, L. Hervas, T. C. Herwig, G. G. Hesketh, N. P. Hessey, J. W. Hetherly, S. Higashino, E. Higón-Rodriguez, K. Hildebrand, E. Hill, J. C. Hill, K. H. Hiller, S. J. Hillier, M. Hils, I. Hinchliffe, M. Hirose, D. Hirschbuehl, B. Hiti, O. Hladik, D. R. Hlaluku, X. Hoad, J. Hobbs, N. Hod, M. C. Hodgkinson, P. Hodgson, A. Hoecker, M. R. Hoeferkamp, F. Hoenig, D. Hohn, T. R. Holmes, M. Homann, S. Honda, T. Honda, T. M. Hong, B. H. Hooberman, W. H. Hopkins, Y. Horii, A. J. Horton, J-Y. Hostachy, A. Hostiuc, S. Hou, A. Hoummada, J. Howarth, J. Hoya, M. Hrabovsky, J. Hrdinka, I. Hristova, J. Hrivnac, T. Hryn’ova, A. Hrynevich, P. J. Hsu, S.-C. Hsu, Q. Hu, S. Hu, Y. Huang, Z. Hubacek, F. Hubaut, F. Huegging, T. B. Huffman, E. W. Hughes, M. Huhtinen, R. F. H. Hunter, P. Huo, N. Huseynov, J. Huston, J. Huth, R. Hyneman, G. Iacobucci, G. Iakovidis, I. Ibragimov, L. Iconomidou-Fayard, Z. Idrissi, P. Iengo, O. Igonkina, T. Iizawa, Y. Ikegami, M. Ikeno, Y. Ilchenko, D. Iliadis, N. Ilic, F. Iltzsche, G. Introzzi, P. Ioannou, M. Iodice, K. Iordanidou, V. Ippolito, M. F. Isacson, N. Ishijima, M. Ishino, M. Ishitsuka, C. Issever, S. Istin, F. Ito, J. M. Iturbe Ponce, R. Iuppa, H. Iwasaki, J. M. Izen, V. Izzo, S. Jabbar, P. Jackson, R. M. Jacobs, V. Jain, K. B. Jakobi, K. Jakobs, S. Jakobsen, T. Jakoubek, D. O. Jamin, D. K. Jana, R. Jansky, J. Janssen, M. Janus, P. A. Janus, G. Jarlskog, N. Javadov, T. Javůrek, M. Javurkova, F. Jeanneau, L. Jeanty, J. Jejelava, A. Jelinskas, P. Jenni, C. Jeske, S. Jézéquel, H. Ji, J. Jia, H. Jiang, Y. Jiang, Z. Jiang, S. Jiggins, J. Jimenez Pena, S. Jin, A. Jinaru, O. Jinnouchi, H. Jivan, P. Johansson, K. A. Johns, C. A. Johnson, W. J. Johnson, K. Jon-And, R. W. L. Jones, S. D. Jones, S. Jones, T. J. Jones, J. Jongmanns, P. M. Jorge, J. Jovicevic, X. Ju, A. Juste Rozas, M. K. Köhler, A. Kaczmarska, M. Kado, H. Kagan, M. Kagan, S. J. Kahn, T. Kaji, E. Kajomovitz, C. W. Kalderon, A. Kaluza, S. Kama, A. Kamenshchikov, N. Kanaya, L. Kanjir, V. A. Kantserov, J. Kanzaki, B. Kaplan, L. S. Kaplan, D. Kar, K. Karakostas, N. Karastathis, M. J. Kareem, E. Karentzos, S. N. Karpov, Z. M. Karpova, K. Karthik, V. Kartvelishvili, A. N. Karyukhin, K. Kasahara, L. Kashif, R. D. Kass, A. Kastanas, Y. Kataoka, C. Kato, A. Katre, J. Katzy, K. Kawade, K. Kawagoe, T. Kawamoto, G. Kawamura, E. F. Kay, V. F. Kazanin, R. Keeler, R. Kehoe, J. S. Keller, E. Kellermann, J. J. Kempster, J Kendrick, H. Keoshkerian, O. Kepka, B. P. Kerševan, S. Kersten, R. A. Keyes, M. Khader, F. Khalil-zada, A. Khanov, A. G. Kharlamov, T. Kharlamova, A. Khodinov, T. J. Khoo, V. Khovanskiy, E. Khramov, J. Khubua, S. Kido, C. R. Kilby, H. Y. Kim, S. H. Kim, Y. K. Kim, N. Kimura, O. M. Kind, B. T. King, D. Kirchmeier, J. Kirk, A. E. Kiryunin, T. Kishimoto, D. Kisielewska, V. Kitali, O. Kivernyk, E. Kladiva, T. Klapdor-Kleingrothaus, M. H. Klein, M. Klein, U. Klein, K. Kleinknecht, P. Klimek, A. Klimentov, R. Klingenberg, T. Klingl, T. Klioutchnikova, F. F. Klitzner, E.-E. Kluge, P. Kluit, S. Kluth, E. Kneringer, E. B. F. G. Knoops, A. Knue, A. Kobayashi, D. Kobayashi, T. Kobayashi, M. Kobel, M. Kocian, P. Kodys, T. Koffas, E. Koffeman, N. M. Köhler, T. Koi, M. Kolb, I. Koletsou, A. A. Komar, T. Kondo, N. Kondrashova, K. Köneke, A. C. König, T. Kono, R. Konoplich, N. Konstantinidis, B. Konya, R. Kopeliansky, S. Koperny, A. K. Kopp, K. Korcyl, K. Kordas, A. Korn, A. A. Korol, I. Korolkov, E. V. Korolkova, O. Kortner, S. Kortner, T. Kosek, V. V. Kostyukhin, A. Kotwal, A. Koulouris, A. Kourkoumeli-Charalampidi, C. Kourkoumelis, E. Kourlitis, V. Kouskoura, A. B. Kowalewska, R. Kowalewski, T. Z. Kowalski, C. Kozakai, W. Kozanecki, A. S. Kozhin, V. A. Kramarenko, G. Kramberger, D. Krasnopevtsev, M. W. Krasny, A. Krasznahorkay, D. Krauss, J. A. Kremer, J. Kretzschmar, K. Kreutzfeldt, P. Krieger, K. Krizka, K. Kroeninger, H. Kroha, J. Kroll, J. Kroll, J. Kroseberg, J. Krstic, U. Kruchonak, H. Krüger, N. Krumnack, M. C. Kruse, T. Kubota, H. Kucuk, S. Kuday, J. T. Kuechler, S. Kuehn, A. Kugel, F. Kuger, T. Kuhl, V. Kukhtin, R. Kukla, Y. Kulchitsky, S. Kuleshov, Y. P. Kulinich, M. Kuna, T. Kunigo, A. Kupco, T. Kupfer, O. Kuprash, H. Kurashige, L. L. Kurchaninov, Y. A. Kurochkin, M. G. Kurth, E. S. Kuwertz, M. Kuze, J. Kvita, T. Kwan, D. Kyriazopoulos, A. La Rosa, J. L. La Rosa Navarro, L. La Rotonda, F. La Ruffa, C. Lacasta, F. Lacava, J. Lacey, D. P. J. Lack, H. Lacker, D. Lacour, E. Ladygin, R. Lafaye, B. Laforge, T. Lagouri, S. Lai, S. Lammers, W. Lampl, E. Lançon, U. Landgraf, M. P. J. Landon, M. C. Lanfermann, V. S. Lang, J. C. Lange, R. J. Langenberg, A. J. Lankford, F. Lanni, K. Lantzsch, A. Lanza, A. Lapertosa, S. Laplace, J. F. Laporte, T. Lari, F. Lasagni Manghi, M. Lassnig, T. S. Lau, P. Laurelli, W. Lavrijsen, A. T. Law, P. Laycock, T. Lazovich, M. Lazzaroni, B. Le, O. Le Dortz, E. Le Guirriec, E. P. Le Quilleuc, M. LeBlanc, T. LeCompte, F. Ledroit-Guillon, C. A. Lee, G. R. Lee, S. C. Lee, L. Lee, B. Lefebvre, G. Lefebvre, M. Lefebvre, F. Legger, C. Leggett, G. Lehmann Miotto, X. Lei, W. A. Leight, M. A. L. Leite, R. Leitner, D. Lellouch, B. Lemmer, K. J. C. Leney, T. Lenz, B. Lenzi, R. Leone, S. Leone, C. Leonidopoulos, G. Lerner, C. Leroy, R. Les, A. A. J. Lesage, C. G. Lester, M. Levchenko, J. Levêque, D. Levin, L. J. Levinson, M. Levy, D. Lewis, B. Li, C. Li, H. Li, L. Li, Q. Li, Q. Li, S. Li, X. Li, Y. Li, Z. Liang, B. Liberti, A. Liblong, K. Lie, J. Liebal, W. Liebig, A. Limosani, C. Y. Lin, K. Lin, S. C. Lin, T. H. Lin, R. A. Linck, B. E. Lindquist, A. E. Lionti, E. Lipeles, A. Lipniacka, M. Lisovyi, T. M. Liss, A. Lister, A. M. Litke, B. Liu, H. Liu, H. Liu, J. K. K. Liu, J. Liu, J. B. Liu, K. Liu, L. Liu, M. Liu, Y. L. Liu, Y. Liu, M. Livan, A. Lleres, J. Llorente Merino, S. L. Lloyd, C. Y. Lo, F. Lo Sterzo, E. M. Lobodzinska, P. Loch, F. K. Loebinger, A. Loesle, K. M. Loew, T. Lohse, K. Lohwasser, M. Lokajicek, B. A. Long, J. D. Long, R. E. Long, L. Longo, K. A. Looper, J. A. Lopez, I. Lopez Paz, A. Lopez Solis, J. Lorenz, N. Lorenzo Martinez, M. Losada, P. J. Lösel, X. Lou, A. Lounis, J. Love, P. A. Love, H. Lu, N. Lu, Y. J. Lu, H. J. Lubatti, C. Luci, A. Lucotte, C. Luedtke, F. Luehring, W. Lukas, L. Luminari, O. Lundberg, B. Lund-Jensen, M. S. Lutz, P. M. Luzi, D. Lynn, R. Lysak, E. Lytken, F. Lyu, V. Lyubushkin, H. Ma, L. L. Ma, Y. Ma, G. Maccarrone, A. Macchiolo, C. M. Macdonald, B. Maček, J. Machado Miguens, D. Madaffari, R. Madar, W. F. Mader, A. Madsen, N. Madysa, J. Maeda, S. Maeland, T. Maeno, A. S. Maevskiy, V. Magerl, C. Maiani, C. Maidantchik, T. Maier, A. Maio, O. Majersky, S. Majewski, Y. Makida, N. Makovec, B. Malaescu, Pa. Malecki, V. P. Maleev, F. Malek, U. Mallik, D. Malon, C. Malone, S. Maltezos, S. Malyukov, J. Mamuzic, G. Mancini, I. Mandić, J. Maneira, L. Manhaes de Andrade Filho, J. Manjarres Ramos, K. H. Mankinen, A. Mann, A. Manousos, B. Mansoulie, J. D. Mansour, R. Mantifel, M. Mantoani, S. Manzoni, L. Mapelli, G. Marceca, L. March, L. Marchese, G. Marchiori, M. Marcisovsky, C. A. Marin Tobon, M. Marjanovic, D. E. Marley, F. Marroquim, S. P. Marsden, Z. Marshall, M.U.F. Martensson, S. Marti-Garcia, C. B. Martin, T. A. Martin, V. J. Martin, B. Martin dit Latour, M. Martinez, V. I. Martinez Outschoorn, S. Martin-Haugh, V. S. Martoiu, A. C. Martyniuk, A. Marzin, L. Masetti, T. Mashimo, R. Mashinistov, J. Masik, A. L. Maslennikov, L. H. Mason, L. Massa, P. Mastrandrea, A. Mastroberardino, T. Masubuchi, P. Mättig, J. Maurer, S. J. Maxfield, D. A. Maximov, R. Mazini, I. Maznas, S. M. Mazza, N. C. Mc Fadden, G. Mc Goldrick, S. P. Mc Kee, A. McCarn, R. L. McCarthy, T. G. McCarthy, L. I. McClymont, E. F. McDonald, J. A. Mcfayden, G. Mchedlidze, S. J. McMahon, P. C. McNamara, C. J. McNicol, R. A. McPherson, S. Meehan, T. J. Megy, S. Mehlhase, A. Mehta, T. Meideck, K. Meier, B. Meirose, D. Melini, B. R. Mellado Garcia, J. D. Mellenthin, M. Melo, F. Meloni, A. Melzer, S. B. Menary, L. Meng, X. T. Meng, A. Mengarelli, S. Menke, E. Meoni, S. Mergelmeyer, C. Merlassino, P. Mermod, L. Merola, C. Meroni, F. S. Merritt, A. Messina, J. Metcalfe, A. S. Mete, C. Meyer, J-P. Meyer, J. Meyer, H. Meyer Zu Theenhausen, F. Miano, R. P. Middleton, S. Miglioranzi, L. Mijović, G. Mikenberg, M. Mikestikova, M. Mikuž, M. Milesi, A. Milic, D. A. Millar, D. W. Miller, C. Mills, A. Milov, D. A. Milstead, A. A. Minaenko, Y. Minami, I. A. Minashvili, A. I. Mincer, B. Mindur, M. Mineev, Y. Minegishi, Y. Ming, L. M. Mir, A. Mirto, K. P. Mistry, T. Mitani, J. Mitrevski, V. A. Mitsou, A. Miucci, P. S. Miyagawa, A. Mizukami, J. U. Mjörnmark, T. Mkrtchyan, M. Mlynarikova, T. Moa, K. Mochizuki, P. Mogg, S. Mohapatra, S. Molander, R. Moles-Valls, M. C. Mondragon, K. Mönig, J. Monk, E. Monnier, A. Montalbano, J. Montejo Berlingen, F. Monticelli, S. Monzani, R. W. Moore, N. Morange, D. Moreno, M. Moreno Llácer, P. Morettini, S. Morgenstern, D. Mori, T. Mori, M. Morii, M. Morinaga, V. Morisbak, A. K. Morley, G. Mornacchi, J. D. Morris, L. Morvaj, P. Moschovakos, M. Mosidze, H. J. Moss, J. Moss, K. Motohashi, R. Mount, E. Mountricha, E. J. W. Moyse, S. Muanza, F. Mueller, J. Mueller, R. S. P. Mueller, D. Muenstermann, P. Mullen, G. A. Mullier, F. J. Munoz Sanchez, W. J. Murray, H. Musheghyan, M. Muškinja, A. G. Myagkov, M. Myska, B. P. Nachman, O. Nackenhorst, K. Nagai, R. Nagai, K. Nagano, Y. Nagasaka, K. Nagata, M. Nagel, E. Nagy, A. M. Nairz, Y. Nakahama, K. Nakamura, T. Nakamura, I. Nakano, R. F. Naranjo Garcia, R. Narayan, D. I. Narrias Villar, I. Naryshkin, T. Naumann, G. Navarro, R. Nayyar, H. A. Neal, P. Yu. Nechaeva, T. J. Neep, A. Negri, M. Negrini, S. Nektarijevic, C. Nellist, A. Nelson, M. E. Nelson, S. Nemecek, P. Nemethy, M. Nessi, M. S. Neubauer, M. Neumann, P. R. Newman, T. Y. Ng, Y. S. Ng, T. Nguyen Manh, R. B. Nickerson, R. Nicolaidou, J. Nielsen, N. Nikiforou, V. Nikolaenko, I. Nikolic-Audit, K. Nikolopoulos, J. K. Nilsen, P. Nilsson, Y. Ninomiya, A. Nisati, N. Nishu, R. Nisius, I. Nitsche, T. Nitta, T. Nobe, Y. Noguchi, M. Nomachi, I. Nomidis, M. A. Nomura, T. Nooney, M. Nordberg, N. Norjoharuddeen, O. Novgorodova, M. Nozaki, L. Nozka, K. Ntekas, E. Nurse, F. Nuti, K. O’connor, D. C. O’Neil, A. A. O’Rourke, V. O’Shea, F. G. Oakham, H. Oberlack, T. Obermann, J. Ocariz, A. Ochi, I. Ochoa, J. P. Ochoa-Ricoux, S. Oda, S. Odaka, A. Oh, S. H. Oh, C. C. Ohm, H. Ohman, H. Oide, H. Okawa, Y. Okumura, T. Okuyama, A. Olariu, L. F. Oleiro Seabra, S. A. Olivares Pino, D. Oliveira Damazio, A. Olszewski, J. Olszowska, A. Onofre, K. Onogi, P. U. E. Onyisi, H. Oppen, M. J. Oreglia, Y. Oren, D. Orestano, N. Orlando, R. S. Orr, B. Osculati, R. Ospanov, G. Otero y Garzon, H. Otono, M. Ouchrif, F. Ould-Saada, A. Ouraou, K. P. Oussoren, Q. Ouyang, M. Owen, R. E. Owen, V. E. Ozcan, N. Ozturk, K. Pachal, A. Pacheco Pages, L. Pacheco Rodriguez, C. Padilla Aranda, S. Pagan Griso, M. Paganini, F. Paige, G. Palacino, S. Palazzo, S. Palestini, M. Palka, D. Pallin, E. St. Panagiotopoulou, I. Panagoulias, C. E. Pandini, J. G. Panduro Vazquez, P. Pani, S. Panitkin, D. Pantea, L. Paolozzi, Th. D. Papadopoulou, K. Papageorgiou, A. Paramonov, D. Paredes Hernandez, A. J. Parker, M. A. Parker, K. A. Parker, F. Parodi, J. A. Parsons, U. Parzefall, V. R. Pascuzzi, J. M. Pasner, E. Pasqualucci, S. Passaggio, Fr. Pastore, S. Pataraia, J. R. Pater, T. Pauly, B. Pearson, S. Pedraza Lopez, R. Pedro, S. V. Peleganchuk, O. Penc, C. Peng, H. Peng, J. Penwell, B. S. Peralva, M. M. Perego, D. V. Perepelitsa, F. Peri, L. Perini, H. Pernegger, S. Perrella, R. Peschke, V. D. Peshekhonov, K. Peters, R. F. Y. Peters, B. A. Petersen, T. C. Petersen, E. Petit, A. Petridis, C. Petridou, P. Petroff, E. Petrolo, M. Petrov, F. Petrucci, N. E. Pettersson, A. Peyaud, R. Pezoa, F. H. Phillips, P. W. Phillips, G. Piacquadio, E. Pianori, A. Picazio, M. A. Pickering, R. Piegaia, J. E. Pilcher, A. D. Pilkington, M. Pinamonti, J. L. Pinfold, H. Pirumov, M. Pitt, L. Plazak, M.-A. Pleier, V. Pleskot, E. Plotnikova, D. Pluth, P. Podberezko, R. Poettgen, R. Poggi, L. Poggioli, I. Pogrebnyak, D. Pohl, I. Pokharel, G. Polesello, A. Poley, A. Policicchio, R. Polifka, A. Polini, C. S. Pollard, V. Polychronakos, K. Pommès, D. Ponomarenko, L. Pontecorvo, G. A. Popeneciu, D. M. Portillo Quintero, S. Pospisil, K. Potamianos, I. N. Potrap, C. J. Potter, H. Potti, T. Poulsen, J. Poveda, M. E. Pozo Astigarraga, P. Pralavorio, A. Pranko, S. Prell, D. Price, M. Primavera, S. Prince, N. Proklova, K. Prokofiev, F. Prokoshin, S. Protopopescu, J. Proudfoot, M. Przybycien, A. Puri, P. Puzo, J. Qian, G. Qin, Y. Qin, A. Quadt, M. Queitsch-Maitland, D. Quilty, S. Raddum, V. Radeka, V. Radescu, S. K. Radhakrishnan, P. Radloff, P. Rados, F. Ragusa, G. Rahal, J. A. Raine, S. Rajagopalan, C. Rangel-Smith, T. Rashid, S. Raspopov, M. G. Ratti, D. M. Rauch, F. Rauscher, S. Rave, I. Ravinovich, J. H. Rawling, M. Raymond, A. L. Read, N. P. Readioff, M. Reale, D. M. Rebuzzi, A. Redelbach, G. Redlinger, R. Reece, R. G. Reed, K. Reeves, L. Rehnisch, J. Reichert, A. Reiss, C. Rembser, H. Ren, M. Rescigno, S. Resconi, E. D. Resseguie, S. Rettie, E. Reynolds, O. L. Rezanova, P. Reznicek, R. Rezvani, R. Richter, S. Richter, E. Richter-Was, O. Ricken, M. Ridel, P. Rieck, C. J. Riegel, J. Rieger, O. Rifki, M. Rijssenbeek, A. Rimoldi, M. Rimoldi, L. Rinaldi, G. Ripellino, B. Ristić, E. Ritsch, I. Riu, F. Rizatdinova, E. Rizvi, C. Rizzi, R. T. Roberts, S. H. Robertson, A. Robichaud-Veronneau, D. Robinson, J. E. M. Robinson, A. Robson, E. Rocco, C. Roda, Y. Rodina, S. Rodriguez Bosca, A. Rodriguez Perez, D. Rodriguez Rodriguez, S. Roe, C. S. Rogan, O. Røhne, J. Roloff, A. Romaniouk, M. Romano, S. M. Romano Saez, E. Romero Adam, N. Rompotis, M. Ronzani, L. Roos, S. Rosati, K. Rosbach, P. Rose, N.-A. Rosien, E. Rossi, L. P. Rossi, J. H. N. Rosten, R. Rosten, M. Rotaru, J. Rothberg, D. Rousseau, A. Rozanov, Y. Rozen, X. Ruan, F. Rubbo, F. Rühr, A. Ruiz-Martinez, Z. Rurikova, N. A. Rusakovich, H. L. Russell, J. P. Rutherfoord, N. Ruthmann, E. M. Rüttinger, Y. F. Ryabov, M. Rybar, G. Rybkin, S. Ryu, A. Ryzhov, G. F. Rzehorz, A. F. Saavedra, G. Sabato, S. Sacerdoti, H.F-W. Sadrozinski, R. Sadykov, F. Safai Tehrani, P. Saha, M. Sahinsoy, M. Saimpert, M. Saito, T. Saito, H. Sakamoto, Y. Sakurai, G. Salamanna, J. E. Salazar Loyola, D. Salek, P. H. Sales De Bruin, D. Salihagic, A. Salnikov, J. Salt, D. Salvatore, F. Salvatore, A. Salvucci, A. Salzburger, D. Sammel, D. Sampsonidis, D. Sampsonidou, J. Sánchez, V. Sanchez Martinez, A. Sanchez Pineda, H. Sandaker, R. L. Sandbach, C. O. Sander, M. Sandhoff, C. Sandoval, D. P. C. Sankey, M. Sannino, Y. Sano, A. Sansoni, C. Santoni, H. Santos, I. Santoyo Castillo, A. Sapronov, J. G. Saraiva, B. Sarrazin, O. Sasaki, K. Sato, E. Sauvan, G. Savage, P. Savard, N. Savic, C. Sawyer, L. Sawyer, J. Saxon, C. Sbarra, A. Sbrizzi, T. Scanlon, D. A. Scannicchio, J. Schaarschmidt, P. Schacht, B. M. Schachtner, D. Schaefer, L. Schaefer, R. Schaefer, J. Schaeffer, S. Schaepe, S. Schaetzel, U. Schäfer, A. C. Schaffer, D. Schaile, R. D. Schamberger, V. A. Schegelsky, D. Scheirich, M. Schernau, C. Schiavi, S. Schier, L. K. Schildgen, C. Schillo, M. Schioppa, S. Schlenker, K. R. Schmidt-Sommerfeld, K. Schmieden, C. Schmitt, S. Schmitt, S. Schmitz, U. Schnoor, L. Schoeffel, A. Schoening, B. D. Schoenrock, E. Schopf, M. Schott, J. F. P. Schouwenberg, J. Schovancova, S. Schramm, N. Schuh, A. Schulte, M. J. Schultens, H.-C. Schultz-Coulon, H. Schulz, M. Schumacher, B. A. Schumm, Ph. Schune, A. Schwartzman, T. A. Schwarz, H. Schweiger, Ph. Schwemling, R. Schwienhorst, J. Schwindling, A. Sciandra, G. Sciolla, M. Scornajenghi, F. Scuri, F. Scutti, J. Searcy, P. Seema, S. C. Seidel, A. Seiden, J. M. Seixas, G. Sekhniaidze, K. Sekhon, S. J. Sekula, N. Semprini-Cesari, S. Senkin, C. Serfon, L. Serin, L. Serkin, M. Sessa, R. Seuster, H. Severini, T. Sfiligoj, F. Sforza, A. Sfyrla, E. Shabalina, N. W. Shaikh, L. Y. Shan, R. Shang, J. T. Shank, M. Shapiro, P. B. Shatalov, K. Shaw, S. M. Shaw, A. Shcherbakova, C. Y. Shehu, Y. Shen, N. Sherafati, A. D. Sherman, P. Sherwood, L. Shi, S. Shimizu, C. O. Shimmin, M. Shimojima, I. P. J. Shipsey, S. Shirabe, M. Shiyakova, J. Shlomi, A. Shmeleva, D. Shoaleh Saadi, M. J. Shochet, S. Shojaii, D. R. Shope, S. Shrestha, E. Shulga, M. A. Shupe, P. Sicho, A. M. Sickles, P. E. Sidebo, E. Sideras Haddad, O. Sidiropoulou, A. Sidoti, F. Siegert, Dj. Sijacki, J. Silva, S. B. Silverstein, V. Simak, L. Simic, S. Simion, E. Simioni, B. Simmons, M. Simon, P. Sinervo, N. B. Sinev, M. Sioli, G. Siragusa, I. Siral, S. Yu. Sivoklokov, J. Sjölin, M. B. Skinner, P. Skubic, M. Slater, T. Slavicek, M. Slawinska, K. Sliwa, R. Slovak, V. Smakhtin, B. H. Smart, J. Smiesko, N. Smirnov, S. Yu. Smirnov, Y. Smirnov, L. N. Smirnova, O. Smirnova, J. W. Smith, M. N. K. Smith, R. W. Smith, M. Smizanska, K. Smolek, A. A. Snesarev, I. M. Snyder, S. Snyder, R. Sobie, F. Socher, A. Soffer, A. Søgaard, D. A. Soh, G. Sokhrannyi, C. A. Solans Sanchez, M. Solar, E. Yu. Soldatov, U. Soldevila, A. A. Solodkov, A. Soloshenko, O. V. Solovyanov, V. Solovyev, P. Sommer, H. Son, A. Sopczak, D. Sosa, C. L. Sotiropoulou, S. Sottocornola, R. Soualah, A. M. Soukharev, D. South, B. C. Sowden, S. Spagnolo, M. Spalla, M. Spangenberg, F. Spanò, D. Sperlich, F. Spettel, T. M. Spieker, R. Spighi, G. Spigo, L. A. Spiller, M. Spousta, R. D. St. Denis, A. Stabile, R. Stamen, S. Stamm, E. Stanecka, R. W. Stanek, C. Stanescu, M. M. Stanitzki, B. S. Stapf, S. Stapnes, E. A. Starchenko, G. H. Stark, J. Stark, S. H Stark, P. Staroba, P. Starovoitov, S. Stärz, R. Staszewski, M. Stegler, P. Steinberg, B. Stelzer, H. J. Stelzer, O. Stelzer-Chilton, H. Stenzel, T. J. Stevenson, G. A. Stewart, M. C. Stockton, M. Stoebe, G. Stoicea, P. Stolte, S. Stonjek, A. R. Stradling, A. Straessner, M. E. Stramaglia, J. Strandberg, S. Strandberg, M. Strauss, P. Strizenec, R. Ströhmer, D. M. Strom, R. Stroynowski, A. Strubig, S. A. Stucci, B. Stugu, N. A. Styles, D. Su, J. Su, S. Suchek, Y. Sugaya, M. Suk, V. V. Sulin, DMS Sultan, S. Sultansoy, T. Sumida, S. Sun, X. Sun, K. Suruliz, C. J. E. Suster, M. R. Sutton, S. Suzuki, M. Svatos, M. Swiatlowski, S. P. Swift, I. Sykora, T. Sykora, D. Ta, K. Tackmann, J. Taenzer, A. Taffard, R. Tafirout, E. Tahirovic, N. Taiblum, H. Takai, R. Takashima, E. H. Takasugi, K. Takeda, T. Takeshita, Y. Takubo, M. Talby, A. A. Talyshev, J. Tanaka, M. Tanaka, R. Tanaka, S. Tanaka, R. Tanioka, B. B. Tannenwald, S. Tapia Araya, S. Tapprogge, S. Tarem, G. F. Tartarelli, P. Tas, M. Tasevsky, T. Tashiro, E. Tassi, A. Tavares Delgado, Y. Tayalati, A. C. Taylor, A. J. Taylor, G. N. Taylor, P. T. E. Taylor, W. Taylor, P. Teixeira-Dias, D. Temple, H. Ten Kate, P. K. Teng, J. J. Teoh, F. Tepel, S. Terada, K. Terashi, J. Terron, S. Terzo, M. Testa, R. J. Teuscher, S. J. Thais, T. Theveneaux-Pelzer, F. Thiele, J. P. Thomas, J. Thomas-Wilsker, P. D. Thompson, A. S. Thompson, L. A. Thomsen, E. Thomson, Y. Tian, M. J. Tibbetts, R. E. Ticse Torres, V. O. Tikhomirov, Yu. A. Tikhonov, S. Timoshenko, P. Tipton, S. Tisserant, K. Todome, S. Todorova-Nova, S. Todt, J. Tojo, S. Tokár, K. Tokushuku, E. Tolley, L. Tomlinson, M. Tomoto, L. Tompkins, K. Toms, B. Tong, P. Tornambe, E. Torrence, H. Torres, E. Torró Pastor, J. Toth, F. Touchard, D. R. Tovey, C. J. Treado, T. Trefzger, F. Tresoldi, A. Tricoli, I. M. Trigger, S. Trincaz-Duvoid, M. F. Tripiana, W. Trischuk, B. Trocmé, A. Trofymov, C. Troncon, M. Trottier-McDonald, M. Trovatelli, L. Truong, M. Trzebinski, A. Trzupek, K. W. Tsang, J.C-L. Tseng, P. V. Tsiareshka, G. Tsipolitis, N. Tsirintanis, S. Tsiskaridze, V. Tsiskaridze, E. G. Tskhadadze, I. I. Tsukerman, V. Tsulaia, S. Tsuno, D. Tsybychev, Y. Tu, A. Tudorache, V. Tudorache, T. T. Tulbure, A. N. Tuna, S. Turchikhin, D. Turgeman, I. Turk Cakir, R. Turra, P. M. Tuts, G. Ucchielli, I. Ueda, M. Ughetto, F. Ukegawa, G. Unal, A. Undrus, G. Unel, F. C. Ungaro, Y. Unno, K. Uno, C. Unverdorben, J. Urban, P. Urquijo, P. Urrejola, G. Usai, J. Usui, L. Vacavant, V. Vacek, B. Vachon, K. O. H. Vadla, A. Vaidya, C. Valderanis, E. Valdes Santurio, M. Valente, S. Valentinetti, A. Valero, L. Valéry, S. Valkar, A. Vallier, J. A. Valls Ferrer, W. Van Den Wollenberg, H. van der Graaf, P. van Gemmeren, J. Van Nieuwkoop, I. van Vulpen, M. C. van Woerden, M. Vanadia, W. Vandelli, A. Vaniachine, P. Vankov, G. Vardanyan, R. Vari, E. W. Varnes, C. Varni, T. Varol, D. Varouchas, A. Vartapetian, K. E. Varvell, J. G. Vasquez, G. A. Vasquez, F. Vazeille, D. Vazquez Furelos, T. Vazquez Schroeder, J. Veatch, V. Veeraraghavan, L. M. Veloce, F. Veloso, S. Veneziano, A. Ventura, M. Venturi, N. Venturi, A. Venturini, V. Vercesi, M. Verducci, W. Verkerke, A. T. Vermeulen, J. C. Vermeulen, M. C. Vetterli, N. Viaux Maira, O. Viazlo, I. Vichou, T. Vickey, O. E. Vickey Boeriu, G. H. A. Viehhauser, S. Viel, L. Vigani, M. Villa, M. Villaplana Perez, E. Vilucchi, M. G. Vincter, V. B. Vinogradov, A. Vishwakarma, C. Vittori, I. Vivarelli, S. Vlachos, M. Vogel, P. Vokac, G. Volpi, H. von der Schmitt, E. von Toerne, V. Vorobel, K. Vorobev, M. Vos, R. Voss, J. H. Vossebeld, N. Vranjes, M. Vranjes Milosavljevic, V. Vrba, M. Vreeswijk, R. Vuillermet, I. Vukotic, P. Wagner, W. Wagner, J. Wagner-Kuhr, H. Wahlberg, S. Wahrmund, J. Walder, R. Walker, W. Walkowiak, V. Wallangen, C. Wang, C. Wang, F. Wang, H. Wang, H. Wang, J. Wang, J. Wang, Q. Wang, R.-J. Wang, R. Wang, S. M. Wang, T. Wang, W. Wang, W. Wang, Z. Wang, C. Wanotayaroj, A. Warburton, C. P. Ward, D. R. Wardrope, A. Washbrook, P. M. Watkins, A. T. Watson, M. F. Watson, G. Watts, S. Watts, B. M. Waugh, A. F. Webb, S. Webb, M. S. Weber, S. M. Weber, S. W. Weber, S. A. Weber, J. S. Webster, A. R. Weidberg, B. Weinert, J. Weingarten, M. Weirich, C. Weiser, H. Weits, P. S. Wells, T. Wenaus, T. Wengler, S. Wenig, N. Wermes, M. D. Werner, P. Werner, M. Wessels, T. D. Weston, K. Whalen, N. L. Whallon, A. M. Wharton, A. S. White, A. White, M. J. White, R. White, D. Whiteson, B. W. Whitmore, F. J. Wickens, W. Wiedenmann, M. Wielers, C. Wiglesworth, L. A. M. Wiik-Fuchs, A. Wildauer, F. Wilk, H. G. Wilkens, H. H. Williams, S. Williams, C. Willis, S. Willocq, J. A. Wilson, I. Wingerter-Seez, E. Winkels, F. Winklmeier, O. J. Winston, B. T. Winter, M. Wittgen, M. Wobisch, T. M. H. Wolf, R. Wolff, M. W. Wolter, H. Wolters, V. W. S. Wong, N. L. Woods, S. D. Worm, B. K. Wosiek, J. Wotschack, K. W. Wozniak, M. Wu, S. L. Wu, X. Wu, Y. Wu, T. R. Wyatt, B. M. Wynne, S. Xella, Z. Xi, L. Xia, D. Xu, L. Xu, T. Xu, W. Xu, B. Yabsley, S. Yacoob, D. Yamaguchi, Y. Yamaguchi, A. Yamamoto, S. Yamamoto, T. Yamanaka, F. Yamane, M. Yamatani, T. Yamazaki, Y. Yamazaki, Z. Yan, H. Yang, H. Yang, Y. Yang, Z. Yang, W-M. Yao, Y. C. Yap, Y. Yasu, E. Yatsenko, K. H. Yau Wong, J. Ye, S. Ye, I. Yeletskikh, E. Yigitbasi, E. Yildirim, K. Yorita, K. Yoshihara, C. Young, C. J. S. Young, J. Yu, J. Yu, S. P. Y. Yuen, I. Yusuff, B. Zabinski, G. Zacharis, R. Zaidan, A. M. Zaitsev, N. Zakharchuk, J. Zalieckas, A. Zaman, S. Zambito, D. Zanzi, C. Zeitnitz, G. Zemaityte, A. Zemla, J. C. Zeng, Q. Zeng, O. Zenin, T. Ženiš, D. Zerwas, D. Zhang, D. Zhang, F. Zhang, G. Zhang, H. Zhang, J. Zhang, L. Zhang, L. Zhang, M. Zhang, P. Zhang, R. Zhang, R. Zhang, X. Zhang, Y. Zhang, Z. Zhang, X. Zhao, Y. Zhao, Z. Zhao, A. Zhemchugov, B. Zhou, C. Zhou, L. Zhou, M. Zhou, M. Zhou, N. Zhou, Y. Zhou, C. G. Zhu, H. Zhu, J. Zhu, Y. Zhu, X. Zhuang, K. Zhukov, A. Zibell, D. Zieminska, N. I. Zimine, C. Zimmermann, S. Zimmermann, Z. Zinonos, M. Zinser, M. Ziolkowski, L. Živković, G. Zobernig, A. Zoccoli, R. Zou, M. zur Nedden, L. Zwalinski, ATLAS Collaboration

**Affiliations:** 1 Department of Physics, University of Adelaide, Adelaide, Australia; 2 Physics Department, SUNY Albany, Albany, NY USA; 3 Department of Physics, University of Alberta, Edmonton, AB Canada; 4 Department of Physics, Ankara University, Ankara, Turkey; 5 Istanbul Aydin University, Istanbul, Turkey; 6 Division of Physics, TOBB University of Economics and Technology, Ankara, Turkey; 7 LAPP, CNRS/IN2P3 and Université Savoie Mont Blanc, Annecy-le-Vieux, France; 8 High Energy Physics Division, Argonne National Laboratory, Argonne, IL USA; 9 Department of Physics, University of Arizona, Tucson, AZ USA; 10 Department of Physics, The University of Texas at Arlington, Arlington, TX USA; 11 Physics Department, National and Kapodistrian University of Athens, Athens, Greece; 12 Physics Department, National Technical University of Athens, Zografou, Greece; 13 Department of Physics, The University of Texas at Austin, Austin, TX USA; 14 Institute of Physics, Azerbaijan Academy of Sciences, Baku, Azerbaijan; 15 Institut de Física d’Altes Energies (IFAE), The Barcelona Institute of Science and Technology, Barcelona, Spain; 16 Institute of Physics, University of Belgrade, Belgrade, Serbia; 17 Department for Physics and Technology, University of Bergen, Bergen, Norway; 18 Physics Division, Lawrence Berkeley National Laboratory, University of California, Berkeley, CA USA; 19 Department of Physics, Humboldt University, Berlin, Germany; 20 Albert Einstein Center for Fundamental Physics, Laboratory for High Energy Physics, University of Bern, Bern, Switzerland; 21 School of Physics and Astronomy, University of Birmingham, Birmingham, UK; 22 Department of Physics, Bogazici University, Istanbul, Turkey; 23 Department of Physics Engineering, Gaziantep University, Gaziantep, Turkey; 24 Faculty of Engineering and Natural Sciences, Istanbul Bilgi University, Istanbul, Turkey; 25 Faculty of Engineering and Natural Sciences, Bahcesehir University, Istanbul, Turkey; 26 Centro de Investigaciones, Universidad Antonio Narino, Bogota, Colombia; 27 INFN Sezione di Bologna, Bologna, Italy; 28 Dipartimento di Fisica e Astronomia, Università di Bologna, Bologna, Italy; 29 Physikalisches Institut, University of Bonn, Bonn, Germany; 30 Department of Physics, Boston University, Boston, MA USA; 31 Department of Physics, Brandeis University, Waltham, MA USA; 32 Universidade Federal do Rio De Janeiro COPPE/EE/IF, Rio de Janeiro, Brazil; 33 Electrical Circuits Department, Federal University of Juiz de Fora (UFJF), Juiz de Fora, Brazil; 34 Federal University of Sao Joao del Rei (UFSJ), Sao Joao del Rei, Brazil; 35 Instituto de Fisica, Universidade de Sao Paulo, São Paulo, Brazil; 36 Physics Department, Brookhaven National Laboratory, Upton, NY USA; 37 Transilvania University of Brasov, Brasov, Romania; 38 Horia Hulubei National Institute of Physics and Nuclear Engineering, Bucharest, Romania; 39 Department of Physics, Alexandru Ioan Cuza University of Iasi, Iasi, Romania; 40 Physics Department, National Institute for Research and Development of Isotopic and Molecular Technologies, Cluj-Napoca, Romania; 41 University Politehnica Bucharest, Bucharest, Romania; 42 West University in Timisoara, Timisoara, Romania; 43 Departamento de Física, Universidad de Buenos Aires, Buenos Aires, Argentina; 44 Cavendish Laboratory, University of Cambridge, Cambridge, UK; 45 Department of Physics, Carleton University, Ottawa, ON Canada; 46 CERN, Geneva, Switzerland; 47 Enrico Fermi Institute, University of Chicago, Chicago, IL USA; 48 Departamento de Física, Pontificia Universidad Católica de Chile, Santiago, Chile; 49 Departamento de Física, Universidad Técnica Federico Santa María, Valparaíso, Chile; 50 Institute of High Energy Physics, Chinese Academy of Sciences, Beijing, China; 51 Department of Physics, Nanjing University, Nanjing, Jiangsu China; 52 Physics Department, Tsinghua University, Beijing, 100084 China; 53 University of Chinese Academy of Science (UCAS), Beijing, China; 54 Department of Modern Physics and State Key Laboratory of Particle Detection and Electronics, University of Science and Technology of China, Hefei, Anhui China; 55 School of Physics, Shandong University, Jinan, Shandong China; 56 Department of Physics and Astronomy, Key Laboratory for Particle Physics, Astrophysics and Cosmology, Ministry of Education, Shanghai Key Laboratory for Particle Physics and Cosmology, Shanghai Jiao Tong University, Shanghai (also at PKU-CHEP), Shanghai, China; 57 Université Clermont Auvergne, CNRS/IN2P3, LPC, Clermont-Ferrand, France; 58 Nevis Laboratory, Columbia University, Irvington, NY USA; 59 Niels Bohr Institute, University of Copenhagen, Kobenhavn, Denmark; 60 INFN Gruppo Collegato di Cosenza, Laboratori Nazionali di Frascati, Frascati, Italy; 61 Dipartimento di Fisica, Università della Calabria, Rende, Italy; 62 Faculty of Physics and Applied Computer Science, AGH University of Science and Technology, Krakow, Poland; 63 Marian Smoluchowski Institute of Physics, Jagiellonian University, Krakow, Poland; 64 Institute of Nuclear Physics, Polish Academy of Sciences, Krakow, Poland; 65 Physics Department, Southern Methodist University, Dallas, TX USA; 66 Physics Department, University of Texas at Dallas, Richardson, TX USA; 67 DESY, Hamburg and Zeuthen, Germany; 68 Lehrstuhl für Experimentelle Physik IV, Technische Universität Dortmund, Dortmund, Germany; 69 Institut für Kern- und Teilchenphysik, Technische Universität Dresden, Dresden, Germany; 70 Department of Physics, Duke University, Durham, NC USA; 71 SUPA-School of Physics and Astronomy, University of Edinburgh, Edinburgh, UK; 72 INFN e Laboratori Nazionali di Frascati, Frascati, Italy; 73 Fakultät für Mathematik und Physik, Albert-Ludwigs-Universität, Freiburg, Germany; 74 Departement de Physique Nucleaire et Corpusculaire, Université de Genève, Geneva, Switzerland; 75 INFN Sezione di Genova, Genoa, Italy; 76 Dipartimento di Fisica, Università di Genova, Genoa, Italy; 77 E. Andronikashvili Institute of Physics, Iv. Javakhishvili Tbilisi State University, Tbilisi, Georgia; 78 High Energy Physics Institute, Tbilisi State University, Tbilisi, Georgia; 79 II Physikalisches Institut, Justus-Liebig-Universität Giessen, Giessen, Germany; 80 SUPA-School of Physics and Astronomy, University of Glasgow, Glasgow, UK; 81 II Physikalisches Institut, Georg-August-Universität, Göttingen, Germany; 82 Laboratoire de Physique Subatomique et de Cosmologie, Université Grenoble-Alpes, CNRS/IN2P3, Grenoble, France; 83 Laboratory for Particle Physics and Cosmology, Harvard University, Cambridge, MA USA; 84 Kirchhoff-Institut für Physik, Ruprecht-Karls-Universität Heidelberg, Heidelberg, Germany; 85 Physikalisches Institut, Ruprecht-Karls-Universität Heidelberg, Heidelberg, Germany; 86 Faculty of Applied Information Science, Hiroshima Institute of Technology, Hiroshima, Japan; 87 Department of Physics, The Chinese University of Hong Kong, Shatin, NT Hong Kong; 88 Department of Physics, The University of Hong Kong, Hong Kong, China; 89 Department of Physics, Institute for Advanced Study, The Hong Kong University of Science and Technology, Clear Water Bay, Kowloon, Hong Kong, China; 90 Department of Physics, National Tsing Hua University, Hsinchu, Taiwan; 91 Department of Physics, Indiana University, Bloomington, IN USA; 92 Institut für Astro- und Teilchenphysik, Leopold-Franzens-Universität, Innsbruck, Austria; 93 University of Iowa, Iowa City, IA USA; 94 Department of Physics and Astronomy, Iowa State University, Ames, IA USA; 95 Joint Institute for Nuclear Research, JINR Dubna, Dubna, Russia; 96 KEK, High Energy Accelerator Research Organization, Tsukuba, Japan; 97 Graduate School of Science, Kobe University, Kobe, Japan; 98 Faculty of Science, Kyoto University, Kyoto, Japan; 99 Kyoto University of Education, Kyoto, Japan; 100 Research Center for Advanced Particle Physics and Department of Physics, Kyushu University, Fukuoka, Japan; 101 Instituto de Física La Plata, Universidad Nacional de La Plata and CONICET, La Plata, Argentina; 102 Physics Department, Lancaster University, Lancaster, UK; 103 INFN Sezione di Lecce, Lecce, Italy; 104 Dipartimento di Matematica e Fisica, Università del Salento, Lecce, Italy; 105 Oliver Lodge Laboratory, University of Liverpool, Liverpool, UK; 106 Department of Experimental Particle Physics, Jožef Stefan Institute and Department of Physics, University of Ljubljana, Ljubljana, Slovenia; 107 School of Physics and Astronomy, Queen Mary University of London, London, UK; 108 Department of Physics, Royal Holloway University of London, Surrey, UK; 109 Department of Physics and Astronomy, University College London, London, UK; 110 Louisiana Tech University, Ruston, LA USA; 111 Laboratoire de Physique Nucléaire et de Hautes Energies, UPMC and Université Paris-Diderot and CNRS/IN2P3, Paris, France; 112 Fysiska institutionen, Lunds universitet, Lund, Sweden; 113 Departamento de Fisica Teorica C-15, Universidad Autonoma de Madrid, Madrid, Spain; 114 Institut für Physik, Universität Mainz, Mainz, Germany; 115 School of Physics and Astronomy, University of Manchester, Manchester, UK; 116 CPPM, Aix-Marseille Université and CNRS/IN2P3, Marseille, France; 117 Department of Physics, University of Massachusetts, Amherst, MA USA; 118 Department of Physics, McGill University, Montreal, QC Canada; 119 School of Physics, University of Melbourne, Victoria, Australia; 120 Department of Physics, The University of Michigan, Ann Arbor, MI USA; 121 Department of Physics and Astronomy, Michigan State University, East Lansing, MI USA; 122 INFN Sezione di Milano, Milan, Italy; 123 Dipartimento di Fisica, Università di Milano, Milan, Italy; 124 B.I. Stepanov Institute of Physics, National Academy of Sciences of Belarus, Minsk, Republic of Belarus; 125 Research Institute for Nuclear Problems of Byelorussian State University, Minsk, Republic of Belarus; 126 Group of Particle Physics, University of Montreal, Montreal, QC Canada; 127 P.N. Lebedev Physical Institute of the Russian Academy of Sciences, Moscow, Russia; 128 Institute for Theoretical and Experimental Physics (ITEP), Moscow, Russia; 129 National Research Nuclear University MEPhI, Moscow, Russia; 130 D.V. Skobeltsyn Institute of Nuclear Physics, M.V. Lomonosov Moscow State University, Moscow, Russia; 131 Fakultät für Physik, Ludwig-Maximilians-Universität München, Münich, Germany; 132 Max-Planck-Institut für Physik (Werner-Heisenberg-Institut), Münich, Germany; 133 Nagasaki Institute of Applied Science, Nagasaki, Japan; 134 Graduate School of Science and Kobayashi-Maskawa Institute, Nagoya University, Nagoya, Japan; 135 INFN Sezione di Napoli, Naples, Italy; 136 Dipartimento di Fisica, Università di Napoli, Naples, Italy; 137 Department of Physics and Astronomy, University of New Mexico, Albuquerque, NM USA; 138 Institute for Mathematics, Astrophysics and Particle Physics, Radboud University Nijmegen/Nikhef, Nijmegen, The Netherlands; 139 Nikhef National Institute for Subatomic Physics, University of Amsterdam, Amsterdam, The Netherlands; 140 Department of Physics, Northern Illinois University, DeKalb, IL USA; 141 Budker Institute of Nuclear Physics, SB RAS, Novosibirsk, Russia; 142 Department of Physics, New York University, New York, NY USA; 143 Ohio State University, Columbus, OH USA; 144 Faculty of Science, Okayama University, Okayama, Japan; 145 Homer L. Dodge Department of Physics and Astronomy, University of Oklahoma, Norman, OK USA; 146 Department of Physics, Oklahoma State University, Stillwater, OK USA; 147 Palacký University, RCPTM, Olomouc, Czech Republic; 148 Center for High Energy Physics, University of Oregon, Eugene, OR USA; 149 LAL, Univ. Paris-Sud, CNRS/IN2P3, Université Paris-Saclay, Orsay, France; 150 Graduate School of Science, Osaka University, Osaka, Japan; 151 Department of Physics, University of Oslo, Oslo, Norway; 152 Department of Physics, Oxford University, Oxford, UK; 153 INFN Sezione di Pavia, Pavia, Italy; 154 Dipartimento di Fisica, Università di Pavia, Pavia, Italy; 155 Department of Physics, University of Pennsylvania, Philadelphia, PA USA; 156 National Research Centre “Kurchatov Institute” B.P. Konstantinov Petersburg Nuclear Physics Institute, St. Petersburg, Russia; 157 INFN Sezione di Pisa, Pisa, Italy; 158 Dipartimento di Fisica E. Fermi, Università di Pisa, Pisa, Italy; 159 Department of Physics and Astronomy, University of Pittsburgh, Pittsburgh, PA USA; 160 Laboratório de Instrumentação e Física Experimental de Partículas-LIP, Lisbon, Portugal; 161 Faculdade de Ciências, Universidade de Lisboa, Lisbon, Portugal; 162 Department of Physics, University of Coimbra, Coimbra, Portugal; 163 Centro de Física Nuclear da Universidade de Lisboa, Lisbon, Portugal; 164 Departamento de Fisica, Universidade do Minho, Braga, Portugal; 165 Departamento de Fisica Teorica y del Cosmos, Universidad de Granada, Granada, Spain; 166 Dep Fisica and CEFITEC of Faculdade de Ciencias e Tecnologia, Universidade Nova de Lisboa, Caparica, Portugal; 167 Institute of Physics, Academy of Sciences of the Czech Republic, Praha, Czech Republic; 168 Czech Technical University in Prague, Praha, Czech Republic; 169 Faculty of Mathematics and Physics, Charles University, Prague, Czech Republic; 170 State Research Center Institute for High Energy Physics (Protvino), NRC KI, Protvino, Russia; 171 Particle Physics Department, Rutherford Appleton Laboratory, Didcot, UK; 172 INFN Sezione di Roma, Rome, Italy; 173 Dipartimento di Fisica, Sapienza Università di Roma, Rome, Italy; 174 INFN Sezione di Roma Tor Vergata, Rome, Italy; 175 Dipartimento di Fisica, Università di Roma Tor Vergata, Rome, Italy; 176 INFN Sezione di Roma Tre, Rome, Italy; 177 Dipartimento di Matematica e Fisica, Università Roma Tre, Rome, Italy; 178 Faculté des Sciences Ain Chock, Réseau Universitaire de Physique des Hautes Energies-Université Hassan II, Casablanca, Morocco; 179 Centre National de l’Energie des Sciences Techniques Nucleaires, Rabat, Morocco; 180 Faculté des Sciences Semlalia, Université Cadi Ayyad, LPHEA-Marrakech, Marrakech, Morocco; 181 Faculté des Sciences, Université Mohamed Premier and LPTPM, Oujda, Morocco; 182 Faculté des Sciences, Université Mohammed V, Rabat, Morocco; 183 DSM/IRFU (Institut de Recherches sur les Lois Fondamentales de l’Univers), CEA Saclay (Commissariat à l’Energie Atomique et aux Energies Alternatives), Gif-sur-Yvette, France; 184 Santa Cruz Institute for Particle Physics, University of California Santa Cruz, Santa Cruz, CA USA; 185 Department of Physics, University of Washington, Seattle, WA USA; 186 Department of Physics and Astronomy, University of Sheffield, Sheffield, UK; 187 Department of Physics, Shinshu University, Nagano, Japan; 188 Department Physik, Universität Siegen, Siegen, Germany; 189 Department of Physics, Simon Fraser University, Burnaby, BC Canada; 190 SLAC National Accelerator Laboratory, Stanford, CA USA; 191 Faculty of Mathematics, Physics and Informatics, Comenius University, Bratislava, Slovak Republic; 192 Department of Subnuclear Physics, Institute of Experimental Physics of the Slovak Academy of Sciences, Kosice, Slovak Republic; 193 Department of Physics, University of Cape Town, Cape Town, South Africa; 194 Department of Physics, University of Johannesburg, Johannesburg, South Africa; 195 School of Physics, University of the Witwatersrand, Johannesburg, South Africa; 196 Department of Physics, Stockholm University, Stockholm, Sweden; 197 The Oskar Klein Centre, Stockholm, Sweden; 198 Physics Department, Royal Institute of Technology, Stockholm, Sweden; 199 Departments of Physics and Astronomy and Chemistry, Stony Brook University, Stony Brook, NY USA; 200 Department of Physics and Astronomy, University of Sussex, Brighton, UK; 201 School of Physics, University of Sydney, Sydney, Australia; 202 Institute of Physics, Academia Sinica, Taipei, Taiwan; 203 Department of Physics, Technion: Israel Institute of Technology, Haifa, Israel; 204 Raymond and Beverly Sackler School of Physics and Astronomy, Tel Aviv University, Tel Aviv, Israel; 205 Department of Physics, Aristotle University of Thessaloniki, Thessaloniki, Greece; 206 International Center for Elementary Particle Physics and Department of Physics, The University of Tokyo, Tokyo, Japan; 207 Graduate School of Science and Technology, Tokyo Metropolitan University, Tokyo, Japan; 208 Department of Physics, Tokyo Institute of Technology, Tokyo, Japan; 209 Tomsk State University, Tomsk, Russia; 210 Department of Physics, University of Toronto, Toronto, ON Canada; 211 INFN-TIFPA, Trento, Italy; 212 University of Trento, Trento, Italy; 213 TRIUMF, Vancouver, BC Canada; 214 Department of Physics and Astronomy, York University, Toronto, ON Canada; 215 Faculty of Pure and Applied Sciences, and Center for Integrated Research in Fundamental Science and Engineering, University of Tsukuba, Tsukuba, Japan; 216 Department of Physics and Astronomy, Tufts University, Medford, MA USA; 217 Department of Physics and Astronomy, University of California Irvine, Irvine, CA USA; 218 INFN Gruppo Collegato di Udine, Sezione di Trieste, Udine, Italy; 219 ICTP, Trieste, Italy; 220 Dipartimento di Chimica, Fisica e Ambiente, Università di Udine, Udine, Italy; 221 Department of Physics and Astronomy, University of Uppsala, Uppsala, Sweden; 222 Department of Physics, University of Illinois, Urbana, IL USA; 223 Instituto de Fisica Corpuscular (IFIC), Centro Mixto Universidad de Valencia-CSIC, Valencia, Spain; 224 Department of Physics, University of British Columbia, Vancouver, BC Canada; 225 Department of Physics and Astronomy, University of Victoria, Victoria, BC Canada; 226 Department of Physics, University of Warwick, Coventry, UK; 227 Waseda University, Tokyo, Japan; 228 Department of Particle Physics, The Weizmann Institute of Science, Rehovot, Israel; 229 Department of Physics, University of Wisconsin, Madison, WI USA; 230 Fakultät für Physik und Astronomie, Julius-Maximilians-Universität, Würzburg, Germany; 231 Fakultät für Mathematik und Naturwissenschaften, Fachgruppe Physik, Bergische Universität Wuppertal, Wuppertal, Germany; 232 Department of Physics, Yale University, New Haven, CT USA; 233 Yerevan Physics Institute, Yerevan, Armenia; 234 Centre de Calcul de l’Institut National de Physique Nucléaire et de Physique des Particules (IN2P3), Villeurbanne, France; 235 Academia Sinica Grid Computing, Institute of Physics, Academia Sinica, Taipei, Taiwan; 236 CERN, 1211 Geneva 23, Switzerland

## Abstract

This paper presents single lepton and dilepton kinematic distributions measured in dileptonic $t\bar{t}$ events produced in 20.2$\hbox {fb}^{-1}$ of $\sqrt{s}=8$ TeV *pp* collisions recorded by the ATLAS experiment at the LHC. Both absolute and normalised differential cross-sections are measured, using events with an opposite-charge $e\mu$ pair and one or two *b*-tagged jets. The cross-sections are measured in a fiducial region corresponding to the detector acceptance for leptons, and are compared to the predictions from a variety of Monte Carlo event generators, as well as fixed-order QCD calculations, exploring the sensitivity of the cross-sections to the gluon parton distribution function. Some of the distributions are also sensitive to the top quark pole mass; a combined fit of NLO fixed-order predictions to all the measured distributions yields a top quark mass value of ${m_t^{\mathrm {pole}}}=173.2\pm 0.9\pm 0.8\pm 1.2$ GeV, where the three uncertainties arise from data statistics, experimental systematics, and theoretical sources.

## Introduction

The top quark is the heaviest known fundamental particle, with a mass ($m_t$) that is much larger than any of the other quarks, and close to the scale of electroweak symmetry breaking. The study of its production and decay properties in proton–proton (*pp*) collisions forms an important part of the ATLAS physics program at the CERN Large Hadron Collider (LHC). Due to its large mass and production cross-section, top quark production is also a significant background to many searches for physics beyond the Standard Model, making precise predictions of absolute rates and differential distributions for top quark production a vital tool in fully exploiting the discovery potential of the LHC.

At the LHC, top quarks are primarily produced as quark-antiquark pairs ($t\bar{t}$). The inclusive $t\bar{t}$ production cross-section $\sigma _{t\bar{t}}$ has been calculated at full next-to-next-to-leading-order (NNLO) accuracy in the strong coupling constant $\alpha _{\text {S}}$, including the resummation of next-to-next-to-leading logarithmic (NNLL) soft gluon terms [1–5]. The resulting prediction at a centre-of-mass energy $\sqrt{s}=8$ TeV is ${\sigma _{t\bar{t}}}=252.9\pm 11.7^{+6,4}_{-8.6}$ pb for a top quark mass of 172.5 GeV, calculated using the top++ 2.0 program [6]. The first uncertainty is due to parton distribution function (PDF) and $\alpha _{\text {S}}$ uncertainties, calculated using the PDF4LHC prescription [7] with the MSTW2008 68% [8, 9], CT10 NNLO [10, 11] and NNPDF 2.3 5f FFN [12] PDF sets, and the second to quantum chromodynamics (QCD) scale variations. This prediction, which has a relative precision of 5.5%, agrees with measurements from ATLAS and CMS at $\sqrt{s}=8$ TeV [13–15] which have reached a precision of 3–4%. Measurements in LHC *pp* collisions at $\sqrt{s}=7$ TeV [13, 15] and more recently at $\sqrt{s}=13$ TeV [16, 17] are also in good agreement with the corresponding NNLO + NNLL predictions.

Going beyond the inclusive production cross-section, measurements of $t\bar{t}$ production as a function of the top quark and $t\bar{t}$ system kinematics properties allow the predictions of QCD calculations and Monte Carlo event-generator programs to be probed in more detail. These comparisons are typically more sensitive at the level of normalised differential cross-sections, i.e. shape comparisons, where both experimental and theoretical uncertainties are reduced. Measurements by ATLAS [18–21] and CMS [22–24] have generally demonstrated good agreement with the predictions of leading-order (LO) multi-leg and next-to-leading-order (NLO) event generators and calculations, though the top quark $p_{\text {T}}$ spectrum is measured to be softer than the predictions by both experiments; this distribution appears to be sensitive to the additional corrections contributing at NNLO [25]. Measurements of jet activity in $t\bar{t}$ events [26–29] are also sensitive to gluon radiation and hence the $t\bar{t}$ production dynamics, without the need to fully reconstruct the kinematics of the $t\bar{t}$ system. However, all these measurements require sophisticated unfolding procedures to correct for the detector acceptance and resolution. This leads to significant systematic uncertainties, especially due to modelling of the showers and hadronisation of the quarks produced in the top quark decays, and the measurement of the resulting jets in the detector.

In the Standard Model (SM), the top quark decays almost exclusively to a *W* boson and a *b* quark, and the final state topologies in $t\bar{t}$ production are governed by the decay modes of the *W* bosons. The channel where one *W* boson decays to an electron ($W\rightarrow e\nu$) and the other to a muon ($W\rightarrow \mu \nu$), giving rise to the $e^+\mu ^-\nu \bar{\nu }b\bar{b}$ final state,^1^ is particularly clean and was exploited to make the most precise ATLAS measurements of $\sigma _{t\bar{t}}$ [13, 17]. The leptons carry information about the underlying top quark kinematics, are free of the uncertainties related to the hadronic part of the final state, and are precisely measured in the detector. Measurements of the $t\bar{t}$ differential cross-section as a function of the lepton kinematics therefore have the potential to provide a complementary view of $t\bar{t}$ production and decay dynamics to that provided by the complete reconstruction of the $t\bar{t}$ final state.

This paper reports such a measurement of the absolute and normalised differential cross-sections for $t\bar{t} \rightarrow e\mu \nu \bar{\nu }b\bar{b}$ produced in *pp* collisions at $\sqrt{s}=8$ TeV, as a function of the kinematics of the single leptons and of the dilepton system. Eight differential cross-section distributions are measured: the transverse momentum $p_{\mathrm T}^{\ell }$ and absolute pseudorapidity $|\eta ^{\ell }|$ of the single leptons (identical for electrons and muons), the $p_{\text {T}}$, invariant mass and absolute rapidity of the dilepton system ($p_{\mathrm T}^{e\mu }$, $m^{e\mu }$ and $|y^{e\mu }|$), the azimuthal angle in the transverse plane $\Delta \phi ^{e\mu }$ between the two leptons, the scalar sum $p_{\mathrm T}^{e}+p_{\mathrm T}^{\mu }$ of the $p_{\text {T}}$ of the two leptons, and the sum $E^{e}+E^{\mu }$ of the energies of the two leptons.^2^ The measurements are corrected to particle level and reported in a fiducial volume where both leptons have $p_{\text {T}} >25$ GeV and $|\eta |<2.5$, avoiding extrapolations into regions of leptonic phase space which are not measured. The particle-level definition includes the contribution of events where one or both *W* bosons decay to electrons or muons via leptonic decays of $\tau$-leptons ($t\rightarrow W\rightarrow \tau \rightarrow e/\mu$), but an alternative set of results is provided where the contributions of $\tau$-leptons are removed with a correction derived from simulation. The definition of the fiducial volume does not make any requirement on the presence of jets from the hadronic decay products of the $t\bar{t}$ system. The measurements are made using events with an opposite-charge $e\mu$ pair and one or two *b*-tagged jets, and extrapolated to the fiducial volume (without jet requirements), using an extension of the double-tagging technique used in the inclusive $t\bar{t}$ cross-section measurement [13]. This approach minimises the systematic uncertainties due to the use of jets and *b*-tagging in the experimental event selection. Since the lepton kinematics are precisely measured in the ATLAS detector, a simple bin-by-bin correction technique is adequate to correct for efficiency and resolution effects, without the need for a full unfolding procedure.

The results are compared to the predictions of various NLO and LO multi-leg $t\bar{t}$ event generators, and to fixed-order perturbative QCD predictions from the MCFM [30] program, which is used to explore the sensitivity to PDFs and QCD scale uncertainties. These comparisons are complementary to previous ATLAS analyses exploring how well $t\bar{t}$ event generators can describe the jet activity [27] and production of extra heavy-flavour jets [31] in the $\sqrt{s}=8$ TeV $t\bar{t}$ dilepton sample.

Some of the cross-section distributions are sensitive to the top quark mass, as suggested in Ref. [32], and mass measurements are made by comparing the measured distributions to predictions from both NLO plus parton shower event generators and fixed-order QCD calculations. The former are similar to traditional measurements where the top quark mass is reconstructed from its decay products [33–36], but rely only on the leptonic decay products of the $t\bar{t}$ system and are less sensitive to experimental uncertainties related to the hadronic part of the final state. The measurements based on fixed-order QCD predictions in a well-defined renormalisation scheme correspond more directly to a measurement of the top quark pole mass $m_t^{\mathrm {pole}}$, the mass definition corresponding to that of a free particle, which may differ from that measured in direct reconstruction of the decay products by $O(1\,\mathrm{GeV})$ [37–39]. Previous determinations of $m_t^{\mathrm {pole}}$ from inclusive and differential $t\bar{t}$ cross-section measurements are compatible with the top quark mass measured from direct reconstruction, with uncertainties of 2–3 GeV [13, 15, 40, 41].

The data and Monte Carlo simulation samples used in this analysis are described in Sect. 2, followed by the event reconstruction and selection in Sect. 3, definition and determination of the fiducial differential cross-sections in Sect. 4 and systematic uncertainties in Sect. 5. Results and comparisons with predictions are given in Sect. 6. The ability of the data to constrain the gluon PDF is investigated in Sect. 7 and the determination of the top quark mass is discussed in Sect. 8. Finally, conclusions are given in Sect. 9.

**Table 1:** Summary of simulated event samples used for $t\bar{t}$ signal and background modelling, giving the matrix-element event generator, PDF set, parton shower and associated tune parameter set. More details, including generator version numbers and references, are given in the text

Process	Matrix-element	PDF	Parton shower	Tune	Comments
$t\bar{t}$	Powheg	CT10	Pythia6	P2011C	${h_{\mathrm {damp}}}={m_t}$
	Powheg	CT10	Herwig+Jimmy	AUET2	${h_{\mathrm {damp}}}=\infty$
	MC@NLO	CT10	Herwig+Jimmy	AUET2	
	Alpgen	CTEQ6L1	Herwig+Jimmy	AUET2	incl. $t\bar{t}$ $b\bar{b}$, $t\bar{t}$ $c\bar{c}$
	Powheg	CT10	Pythia6	P2012 radHi	${h_{\mathrm {damp}}}=2{m_t}$, $\frac{1}{2}\mu _{F,R}$
	Powheg	CT10	Pythia6	P2012 radLo	${h_{\mathrm {damp}}}={m_t}$, $2\mu _{F,R}$
*Wt*	Powheg	CT10	Pythia6	P2011C	diagram removal
*Z*, *W*+jets	Alpgen	CTEQ6L1	Pythia6	P2011C	incl. $Zb\bar{b}$
*WW*, *WZ*, *ZZ*	Alpgen	CTEQ6L1	Herwig	AUET2	
$t\bar{t}$ +*W*, *Z*	MadGraph	CTEQ6L1	Pythia6	P2011C	
$W\gamma$+jets	Sherpa	CT10	Sherpa	default	
*t*-channel top	AcerMC	CTEQ6L1	Pythia6	AUET2B	

## Data and simulated samples

The ATLAS detector [42] at the LHC covers nearly the entire solid angle around the collision point, and consists of an inner tracking detector surrounded by a thin superconducting solenoid magnet producing a 2 T axial magnetic field, electromagnetic and hadronic calorimeters, and an external muon spectrometer incorporating three large toroidal magnet assemblies. The analysis was performed on a sample of proton–proton collision data at $\sqrt{s}=8$ TeV recorded by the ATLAS detector in 2012, corresponding to an integrated luminosity of 20.2 $\hbox {fb}^{-1}$. Events were required to pass a single-electron or single-muon trigger, with thresholds set to be fully efficient for leptons with $p_{\text {T}} >25$ GeV passing offline selections. Each triggered event also includes the signals from on average 20 additional inelastic *pp* collisions in the same bunch crossing, referred to as pileup.

Monte Carlo simulated event samples were used to develop the analysis procedures, to compare with data, and to evaluate signal efficiencies and background contributions. An overview of the samples used for signal and background modelling is shown in Table 1, and further details are given below. Samples were processed using either the full ATLAS detector simulation [43] based on GEANT4 [44], or a faster simulation making use of parameterised showers in the calorimeters [45]. The effects of pileup were simulated by generating additional inelastic *pp* collisions with Pythia8 [46] using the A2 parameter set (tune) [47] and overlaying them on the primary simulated events. These combined events were then processed using the same reconstruction and analysis chain as the data. Small corrections were applied to the lepton trigger and selection efficiencies better to model the performance measured in data.

The baseline simulated $t\bar{t}$ sample was produced using the NLO matrix element event generator Powheg-Box v1.0 (referred to hereafter as Powheg) [48–51] using the CT10 PDFs [10], interfaced to Pythia6 (version 6.426) [52] with the CTEQ6L1 PDF set [53] and the Perugia 2011C (P2011C) tune [54] for parton shower, hadronisation and underlying event modelling. This setup provides an NLO QCD prediction of the $t\bar{t}$ production process, a leading-order prediction for the top quark decays, and an approximate treatment of the spin correlations between the quark and antiquark. The Powheg parameter $h_{\mathrm {damp}}$, used in the damping function that limits the resummation of higher-order effects incorporated into the Sudakov form factor, was set to $m_t$. This value was found to give a better modelling of the $t\bar{t}$ system $p_{\text {T}}$ at $\sqrt{s}=7$ TeV [55] than the setting of ${h_{\mathrm {damp}}}=\infty$ used for the baseline $t\bar{t}$ sample in Ref. [13], which corresponds to no damping.

Alternative $t\bar{t}$ simulation samples used to evaluate systematic uncertainties were generated with Powheg interfaced to Herwig (version 6.520) [56, 57] with the ATLAS AUET2 tune [58] and Jimmy (version 4.31) [59] for underlying event modelling, with MC@NLO (version 4.01) [60, 61] interfaced to Herwig + Jimmy, and with the leading-order ‘multi-leg’ event generator Alpgen (version 2.13) [62], also interfaced to Herwig + Jimmy. The Alpgen samples used leading-order matrix elements for $t\bar{t}$ production accompanied by up to three additional light partons, and dedicated matrix elements for $t\bar{t}$ plus $b\bar{b}$ or $c\bar{c}$ production, together with the MLM parton-jet matching scheme [63] to account for double-counting of configurations generated by both the parton shower and matrix-element calculation. The effects of additional radiation in $t\bar{t}$ events were further studied using two additional Powheg + Pythia6 samples, one using the Perugia 2012 radHi tune [54], with $h_{\mathrm {damp}}$ set to $2{m_t}$ and factorisation and renormalisation scales $\mu _F$ and $\mu _R$ reduced from their event generator defaults by a factor of two, giving more parton shower radiation; and one with the Perugia 2012 radLo tune [54], $\mu _F$ and $\mu _R$ increased by a factor of two and ${h_{\mathrm {damp}}}={m_t}$, giving less parton shower radiation. The parameters of these samples were chosen to span the uncertainties in jet observables measured by ATLAS in $t\bar{t}$ events at $\sqrt{s}=7$ TeV [26, 55, 64]. The top quark mass was set to 172.5 GeV in all these samples, consistent with recent measurements by ATLAS [35] and CMS [36]. They were all normalised to the NNLO + NNLL cross-section prediction discussed in Sect. 1 when comparing simulation with data. Further $t\bar{t}$ simulation samples with different event generator setups were used for comparisons with the measured differential cross-sections as discussed in Sect. 6.2, and in the extraction of the top quark mass as discussed in Sect. 8.

Backgrounds to the $t\bar{t}$ event selection are classified into two types: those with two real prompt leptons from *W* or *Z* boson decays (including those produced via leptonic $\tau$ decays), and those where one of the reconstructed lepton candidates is misidentified, i.e. a non-prompt lepton from the decay of a bottom or charm hadron, an electron from a photon conversion, hadronic jet activity misidentified as an electron, or a muon produced from the decay in flight of a pion or kaon. The first category is dominated by the associated production of a *W* boson and a single top quark, *Wt*, that is simulated using Powheg + Pythia6 with the CT10 PDFs and the P2011C tune. The ‘diagram removal’ scheme was used to handle the interference between the $t\bar{t}$ and *Wt* final states that occurs at NLO [65, 66]. Smaller backgrounds result from $Z\rightarrow \tau \tau (\rightarrow e\mu )$+jets, modelled using Alpgen + Pythia6 including leading-order matrix elements for $Zb\bar{b}$ production, and diboson (*WW*, *WZ* and *ZZ*) production in association with jets, modelled with Alpgen + Herwig + Jimmy. The *Wt* background was normalised to the approximate NNLO cross-section of $22.4\pm 1.5$ pb, determined as in Ref. [67]. The inclusive *Z* cross-section was set to the NNLO prediction from FEWZ [68], but the normalisation of the $Z\rightarrow \tau \tau$ background with *b*-tagged jets was determined with the help of data control samples as discussed in Sect. 4.2. The small diboson background was normalised to the NLO QCD inclusive cross-section predictions calculated with MCFM [69], using the Alpgen + Herwig prediction for the fraction of diboson events with extra jets. Production of $t\bar{t}$ in association with a *W* or *Z* boson, which contributes to the control sample with two same-charge leptons, was simulated with MadGraph [70] interfaced to Pythia6 with CTEQ6L1 PDFs, and normalised to NLO cross-section predictions [71, 72].

Backgrounds with one real and one misidentified lepton arise from $t\bar{t}$ events with one hadronically-decaying *W*; *W*+jets production, modelled as described above for *Z*+jets; $W\gamma$+jets, modelled with Sherpa 1.4.1 [73] with CT10 PDFs; and *t*-channel single top production, modelled with AcerMC [74] with the AUET2B tune [75] and CTEQ6L1 PDFs interfaced to Pythia6. The normalisations of these backgrounds in the opposite-charge $e\mu$ samples were determined with the help of the corresponding same-charge $e\mu$ samples in data. Other backgrounds, including processes with two misidentified leptons, are negligible after the event selections used in this analysis.

## Event reconstruction and selection

The analysis makes use of reconstructed electrons, muons, and *b*-tagged jets, selected exactly as described in Ref. [13]. In brief, electron candidates [76] were required to satisfy $E_{\text {T}} >25$ GeV and $|\eta |<2.47$, and to not lie within the transition region $1.37<|\eta |<1.52$ between the barrel and endcap electromagnetic calorimeters. Muon candidates [77] were required to satisfy $p_{\text {T}} >25$ GeV and $|\eta |<2.5$. In order to reduce background from non-prompt leptons, electrons were required to be isolated from nearby hadronic activity using both calorimeter and tracking information, and muons were required to be isolated using tracking information alone. Jets were reconstructed using the anti-$k_t$ algorithm [78, 79] with radius parameter $R=0.4$ using calorimeter energy clusters calibrated with the local cluster weighting method [80]. Jets were further calibrated using information from both simulation and data [81, 82], and required to satisfy $p_{\text {T}} >25$ GeV and $|\eta |<2.5$. Jets satisfying $p_{\text {T}} <50$ GeV and $|\eta |<2.4$ were additionally required to pass pileup rejection criteria based on their associated tracks [82]. To further suppress non-isolated leptons likely to originate from heavy-flavour decays within jets, electron and muon candidates within $\Delta R<0.4$ of selected jets were discarded. Finally, jets likely to contain *b*-hadrons were *b*-tagged using the MV1 algorithm [83], a multivariate discriminant making use of track impact parameters and reconstructed secondary vertices. A tagging working point corresponding to a 70% efficiency for tagging *b*-quark jets from top decays in $t\bar{t}$ events was used, giving a rejection factor of about 140 against light-quark and gluon jets, and about five against jets originating from charm quarks.

**Table 2:** Observed numbers of opposite-sign $e\mu$ events with one and two *b*-tagged jets ($N_1$ and $N_2$) together with the estimates of backgrounds and associated total uncertainties described in Sect. 5

Event counts	$N_1$	$N_2$
Data	21666	11739
*Wt* single top	$2080\pm 210$	$350\pm 120$
$Z(\rightarrow \tau \tau \rightarrow e\mu )$+jets	$210\pm 40$	$7\pm 2$
Diboson	$120\pm 30$	$3\pm 1$
Misidentified leptons	$220\pm 80$	$78\pm 50$
Total background	$2630\pm 230$	$440\pm 130$

As in Ref. [13], events were required to have at least one reconstructed primary vertex^3^ and to have no jets with $p_{\text {T}} >20$ GeV failing jet quality requirements [81]. Events having muons compatible with cosmic-ray interactions or losing substantial energy following bremsstrahlung in the calorimeter material were rejected. A preselection requiring exactly one electron and one muon selected as described above was then applied, requiring at least one selected lepton to be matched to a corresponding electron or muon trigger signature. Events with an opposite-charge-sign $e\mu$ pair formed the main analysis sample, with events having a same-sign pair being used to estimate the background from misidentified leptons.

**Fig. 1:**
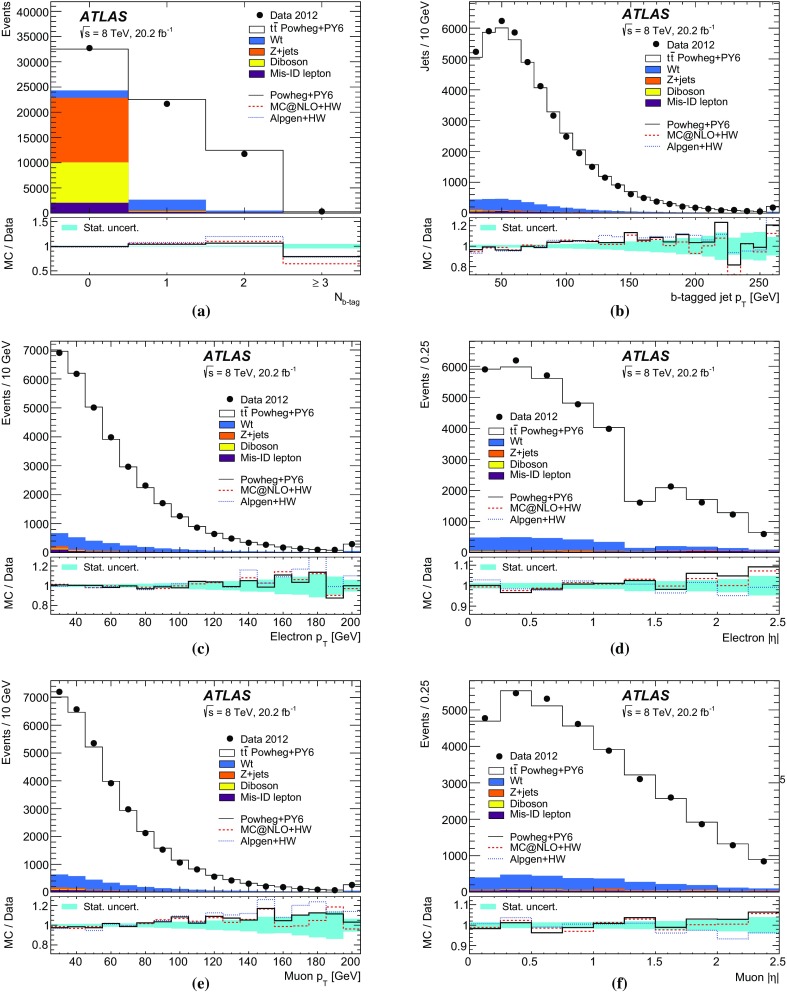
Distributions of **a** the number of *b*-tagged jets in preselected opposite-sign $e\mu$ events; and **b** the $p_{\text {T}}$ of *b*-tagged jets, **c** the $p_{\text {T}}$ of the electron, **d** the $|\eta |$ of the electron, **e** the $p_{\text {T}}$ of the muon and **f** the $|\eta |$ of the muon, in events with an opposite-sign $e\mu$ pair and at least one *b*-tagged jet. The reconstruction-level data are compared to the expectation from simulation, broken down into contributions from $t\bar{t}$  (Powheg + Pythia6), single top, *Z*+jets, dibosons, and events with misidentified electrons or muons. The simulation prediction is normalised to the same integrated luminosity as the data in **a** and to the same number of entries as the data in **b**–**f**. The lower parts of the figure show the ratios of simulation to data, using various $t\bar{t}$ signal samples and with the cyan band indicating the data statistical uncertainty. The last bin includes the overflow in panels **b**, **c** and **e**

**Fig. 2:**
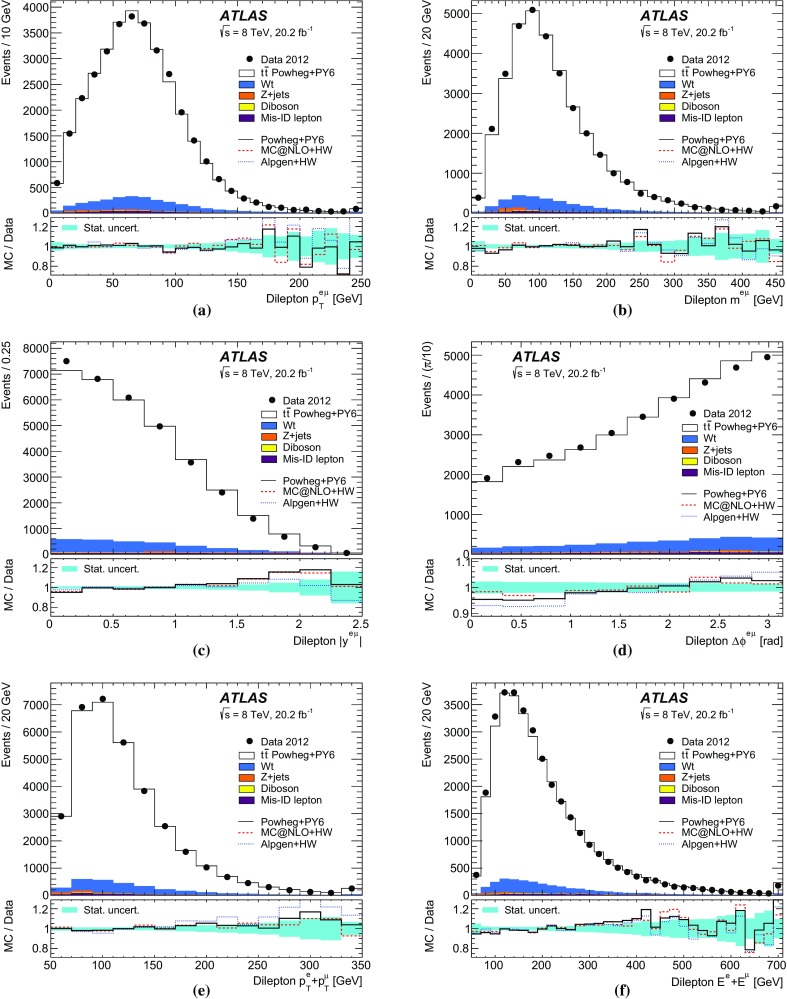
Distributions of **a** the dilepton $p_{\mathrm T}^{e\mu }$, **b** invariant mass $m^{e\mu }$, **c** rapidity $|y^{e\mu }|$, **d** azimuthal angle difference $\Delta \phi ^{e\mu }$, **e** lepton $p_{\text {T}}$ sum $p_{\mathrm T}^{e}+p_{\mathrm T}^{\mu }$ and **f** lepton energy sum $E^{e}+E^{\mu }$, in events with an opposite-sign $e\mu$ pair and at least one *b*-tagged jet. The reconstruction-level data are compared to the expectation from simulation, broken down into contributions from $t\bar{t}$  (Powheg + Pythia6), single top, *Z*+jets, dibosons, and events with misidentified electrons or muons, normalised to the same number of entries as the data. The lower parts of the figure show the ratios of simulation to data, using various $t\bar{t}$ signal samples and with the cyan band indicating the data statistical uncertainty. The last bin includes the overflow in panels **a**, **b**, **e** and **f**

A total of 66,453 data events passed the opposite-sign $e\mu$ preselection. Events were then further sub-divided according to the number of *b*-tagged jets, irrespective of the number of untagged jets, and events having one or two *b*-tagged jets were retained for further analysis. The numbers of one and two *b*-tagged jet events selected in data are shown in Table 2, compared with expected non-$t\bar{t}$ contributions from *Wt* and dibosons evaluated from simulation, and $Z(\rightarrow \tau \tau \rightarrow e\mu )$+jets and misidentified leptons evaluated from data and simulation, as discussed in detail in Sects. 4.2 and 5 below.^4^ In simulation, the one *b*-tagged sample is about 88% pure and the two *b*-tagged sample 96% pure in $t\bar{t}$ events, with the largest backgrounds coming from *Wt* production in both cases. The distribution of the number of *b*-tagged jets in preselected opposite-sign $e\mu$ events is shown in Fig. 1a, compared to the predictions from simulation using Powheg + Pythia6 (PY6), MC@NLO + Herwig (HW) and Alpgen + Herwig $t\bar{t}$ samples, normalising the total simulation prediction in each case using the integrated luminosity of the data sample. The distributions of the $p_{\text {T}}$ of *b*-tagged jets, and the reconstructed electron and muon $p_{\text {T}}$ and $|\eta |$ in events with at least one *b*-tagged jet are shown in Fig. 1b–f, with the total simulation prediction normalised to the same number of events as the data to facilitate shape comparisons. The distributions of the reconstructed dilepton variables $p_{\mathrm T}^{e\mu }$, $m^{e\mu }$, $|y^{e\mu }|$, $\Delta \phi ^{e\mu }$, $p_{\mathrm T}^{e}+p_{\mathrm T}^{\mu }$ and $E^{e}+E^{\mu }$ are shown in Fig. 2, with the simulation normalised as for Fig. 1b–f. In general the data are well described by the predictions using the different $t\bar{t}$ models, but a few differences are visible. The lepton $p_{\text {T}}$ spectra are softer in data than in simulation, the lepton $|\eta ^{\ell }|$ and dilepton $|y^{e\mu }|$ distributions are more central than the Powheg + Pythia6 and MC@NLO + Herwig predictions, and the $\Delta \phi ^{e\mu }$ distribution is slightly flatter in data than in all the predictions.

## Fiducial cross-section determination

The cross-section measurements were made for a fiducial region, where the particle-level electron and muon were required to have opposite charge signs, to each come from *W* decays either directly or via $W\rightarrow \tau \rightarrow e/\mu$ and to each satisfy $p_{\text {T}} >25$ GeV and $|\eta |<2.5$. The lepton four-momenta were taken after final-state radiation, and ‘dressed’ by including the four-momenta of any photons within a cone of size $\Delta R=0.1$ around the lepton direction, excluding photons produced from hadronic decays or interactions with the detector material. The total cross-section within this fiducial volume corresponds to the fiducial cross-section measured in Ref. [13]. According to the predictions of the baseline Powheg + Pythia6 $t\bar{t}$ simulation, it is about 44% of the total $t\bar{t} \rightarrow e\mu \nu \bar{\nu }b\bar{b}$ cross-section without restrictions on the lepton acceptance and including contributions via $W\rightarrow \tau \rightarrow e/\mu$.

### Cross-section extraction

The differential cross-sections were measured using an extension of the technique used in Ref. [13], counting the number of leptons or events with one ($N^i_1$) or two ($N^i_2$) *b*-tagged jets where the lepton(s) fall in bin *i* of a differential distribution at reconstruction level. For the single-lepton distributions $p_{\mathrm T}^{\ell }$ and $|\eta ^{\ell }|$, there are two counts per event, in the two bins corresponding to the electron and muon. For the dilepton distributions, each event contributes a single count corresponding to the bin in which the appropriate dilepton variable falls. For each measured distribution, these counts satisfy the tagging equations:

1 $$\begin{aligned} \begin{array}{lll} {N^i_1}&{} = &{} L {\sigma ^i_{t\bar{t}}}\ {G^i_{e\mu }}2{\epsilon ^i_{b}}(1-{C^i_b}{\epsilon ^i_{b}}) + {N_1^{i,\mathrm {bkg}}}, \\ *[2mm] {N^i_2}&{} = &{} L {\sigma ^i_{t\bar{t}}}\ {G^i_{e\mu }}{C^i_b}({\epsilon ^i_{b}})^2 + {N_2^{i,\mathrm {bkg}}}, \end{array} \end{aligned}$$

where $\sigma ^i_{t\bar{t}}$ is the absolute fiducial differential cross-section in bin *i*, and *L* is the integrated luminosity of the sample. The reconstruction efficiency $G^i_{e\mu }$ represents the ratio of the number of reconstructed $e\mu$ events (or leptons for $p_{\mathrm T}^{\ell }$ and $|\eta ^{\ell }|$) falling in bin *i* at reconstruction level to the number of true $e\mu$ events (or leptons) falling in the same bin at particle level, evaluated using $t\bar{t}$ simulation without making any requirements on reconstructed or particle-level jets. It therefore corrects for both the lepton reconstruction efficiency and bin migration, where events corresponding to bin *j* at particle level appear in a different bin $i\ne j$ at reconstruction level. The values of $G^i_{e\mu }$ in simulation are typically in the range 0.5–0.6, with some dependence on lepton kinematics due to the varying reconstruction efficiencies with lepton $|\eta |$ and $p_{\text {T}}$, and the effect of isolation requirements when the leptons are close together in the detector.

The efficiency $\epsilon ^i_{b}$ represents the combined probability for a jet from the quark *q* in the $t\rightarrow Wq$ decay to fall within the detector acceptance, be reconstructed as a jet with $p_{\text {T}} >25$ GeV and be tagged as a *b*-jet. Although this quark is almost always a *b*-quark, $\epsilon ^i_{b}$ also accounts for the 0.2% of top quarks that decay to *Ws* or *Wd*. If the kinematics of the two *b* quarks produced in the top quark decays are uncorrelated, the probability to tag both is given by ${\epsilon ^i_{bb}}=({\epsilon ^i_{b}})^2$. In practice, small correlations are present, for example due to kinematic correlations between the *b*-jets from the top quark decays, or extra $b\bar{b}$ or $c\bar{c}$ pairs produced in association with the $t\bar{t}$ system [13]. Their effects are corrected via the tagging correlation coefficient ${C^i_b}={\epsilon ^i_{bb}}/({\epsilon ^i_{b}})^2$, whose values are taken from $t\bar{t}$ simulation. They depend slightly on the bin *i* of the dilepton system but are always within 1–2% of unity, even for the bins at the edges of the differential distributions. The correlation $C^i_b$ also corrects for the small effects on $N^i_1$, $N^i_2$ and $\epsilon ^i_{b}$ of the small fraction of $t\bar{t}$ events which have additional *b* quarks produced in association with the $t\bar{t}$ system, and the even smaller effects from mistagged light quark, charm or gluon jets in $t\bar{t}$ events. This formalism involving $\epsilon ^i_{b}$ and $C^i_b$ allows the fraction of top quarks where the jet was not reconstructed to be inferred from the counts $N^i_1$ and $N^i_2$, minimising the exposure to systematic uncertainties from jet measurements and *b*-tagging, and allowing the fiducial cross-sections $\sigma ^i_{t\bar{t}}$ to be defined with no requirements on the jets in the final state.

Backgrounds from sources other than $t\bar{t} \rightarrow e\mu \nu \bar{\nu }b\bar{b}$ events also contribute to the counts $N^i_1$ and $N^i_2$, and are represented by the terms $N_1^{i,\mathrm {bkg}}$ and $N_2^{i,\mathrm {bkg}}$ in Eq. (1). These contributions were evaluated using a combination of simulation- and data-based methods as discussed in Sect. 4.2 below.

The tagging equations were solved numerically in each bin *i* of each differential distribution separately. The bin ranges for each distribution were chosen according to the experimental resolution, minimising the bin-to-bin migration by keeping the bin purities (the fractions of reconstructed events in bin *i* that originate from events which are also in bin *i* at particle level) above about 0.9. The resolution on the reconstructed kinematic quantities is dominated by the electron energy and muon momentum measurements, and the purities for the distributions which depend mainly on angular variables are higher, around 0.96 for $|y^{e\mu }|$ and 0.99 for $|\eta ^{\ell }|$ and $\Delta \phi ^{e\mu }$. For these distributions, the bin ranges were chosen so as to give about ten bins for each distribution. The bin range choices for all distributions can be seen in Tables 3, 4, 5 and 6 in Sect. 6, and the last bin of the $p_{\mathrm T}^{\ell }$, $p_{\mathrm T}^{e\mu }$, $m^{e\mu }$, $p_{\mathrm T}^{e}+p_{\mathrm T}^{\mu }$ and $E^{e}+E^{\mu }$ distributions includes overflow events falling above the last bin boundary, indicated by the ‘+’ sign after the upper bin limit.

The normalised fiducial differential cross-section distributions $\varsigma ^i_{t\bar{t}}$ were calculated from the absolute cross-sections $\sigma ^i_{t\bar{t}}$ determined from Eq. (1) as follows:

2 $$\begin{aligned} {\varsigma ^i_{t\bar{t}}}= \frac{{\sigma ^i_{t\bar{t}}}}{\Sigma _j\ {\sigma ^j_{t\bar{t}}}} = \frac{{\sigma ^i_{t\bar{t}}}}{{\sigma ^{t\bar{t}}_{\mathrm {fid}}}}, \end{aligned}$$

where $\sigma ^{t\bar{t}}_{\mathrm {fid}}$ is the total cross-section summed over all bins of the fiducial region. The $\varsigma ^i_{t\bar{t}}$ values are divided by the bin widths $W_i$, to produce the cross-sections differential in the variable *x* ($x={p_{\mathrm T}^{\ell }}$, $|\eta ^{\ell }|$, etc.):

$$\begin{aligned} \frac{1}{\sigma }\left( \frac{{\mathrm d}\sigma }{{\mathrm d}x}\right) _i = \frac{{\varsigma ^i_{t\bar{t}}}}{W_i}\ . \end{aligned}$$

The normalisation condition in Eq. (2) induces a statistical correlation between the normalised measurements in each bin. The absolute dilepton cross-section measurements are not statistically correlated between bins, but kinematic correlations between the electron and muon in each event induce small statistical correlations between bins of the absolute single lepton $p_{\mathrm T}^{\ell }$ and $|\eta ^{\ell }|$ distributions, as discussed in Sect. 4.3 below.

The measured cross-sections include contributions where one or both leptons are produced via leptonic tau decays ($t\rightarrow W\rightarrow \tau \rightarrow e/\mu$), but the fixed-order predictions discussed in Sect. 6.3 only include the direct decays $t\rightarrow W\rightarrow e/\mu$. To allow comparison with such predictions, a second set of cross-section results were derived with a bin-by-bin multiplicative correction $f^i_{\bar{\tau }}$ to remove the $\tau$ contributions:

3 $$\begin{aligned} {\sigma ^i_{t\bar{t}}}\,(\text{ no-- }\tau ) = {f^i_{\bar{\tau }}}{\sigma ^i_{t\bar{t}}}\ , \end{aligned}$$

and similarly for the normalised cross-sections ${\varsigma ^i_{t\bar{t}}}\,(\text{ no- }\tau )$. The corrections $f^i_{\bar{\tau }}$ were evaluated from the baseline Powheg + Pythia6 $t\bar{t}$ simulation and are typically close to 0.9, decreasing to 0.8–0.85 at low lepton $p_{\text {T}}$.

### Background estimates

**Fig. 3:**
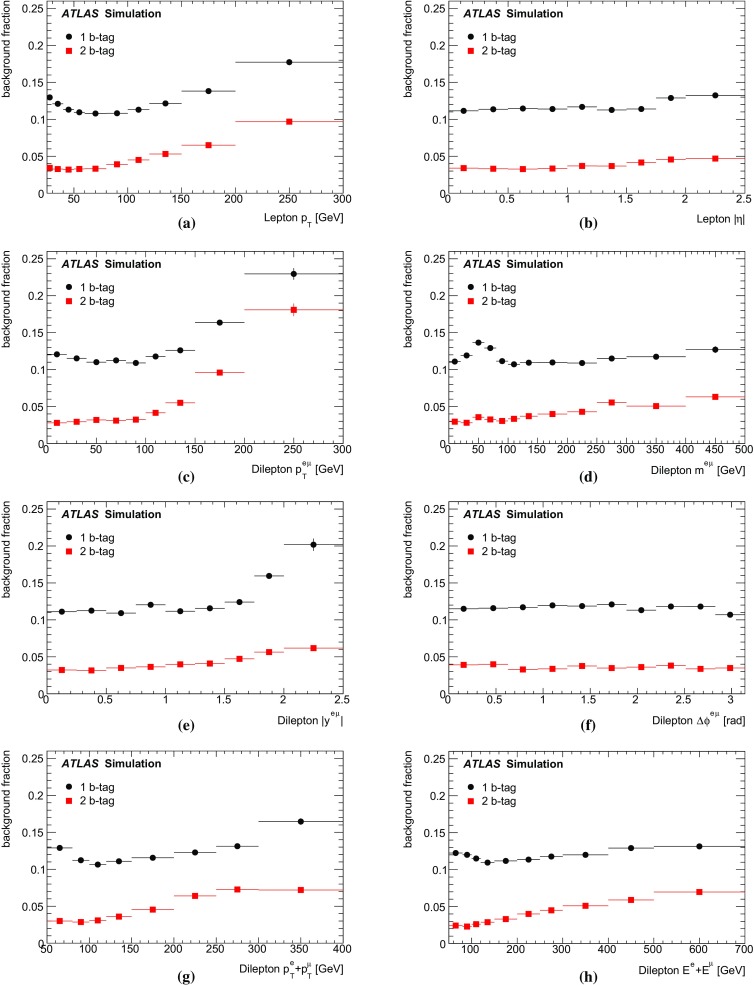
Estimated background fractions in the one and two *b*-tagged samples as functions of each lepton and dilepton differential variable, estimated from simulation alone. The error bars correspond to the statistical uncertainties of the simulation samples, and are often smaller than the marker size

The *Wt* single top and diboson backgrounds were estimated from simulation using the samples discussed in Sect. 2, whilst the *Z*+jets background (with $Z\rightarrow \tau \tau \rightarrow e\mu 4\nu$) and the contribution from events with one real and one misidentified lepton were estimated using both simulation and data as discussed below. The backgrounds in both the one and two *b*-tagged samples are dominated by *Wt* (see Table 2). The total background fraction (i.e. the predicted fraction of events in each bin which do not come from $t\bar{t}$ with two real prompt leptons) varies significantly as a function of some of the differential variables, as shown in Fig. 3. This variation is taken into account by estimating the background contributions $N_1^{i,\mathrm {bkg}}$ and $N_2^{i,\mathrm {bkg}}$ separately in each bin of each differential distribution.

The production cross-sections for *Z* bosons accompanied by heavy-flavour jets are subject to large theoretical uncertainties. The background predictions from Alpgen + Pythia6 in each bin of each distribution were therefore normalised from data, by multiplying them by constant scale factors of $1.4\pm 0.2$ for the one *b*-tagged jet sample and $1.1\pm 0.3$ for the two *b*-tagged jet sample. These scale factors were derived from the comparison of data and simulated event yields for $Z\rightarrow ee$ and $Z\rightarrow \mu \mu$ plus one or two *b*-tagged jets, inclusively for all lepton pairs passing the kinematic selections for electrons and muons [13]. The uncertainties are dominated by the dependence of the scale factors on lepton kinematics, investigated by studying their variation with *Z*-boson $p_{\text {T}}$, reconstructed from the *ee* or $\mu \mu$ system.

**Fig. 4:**
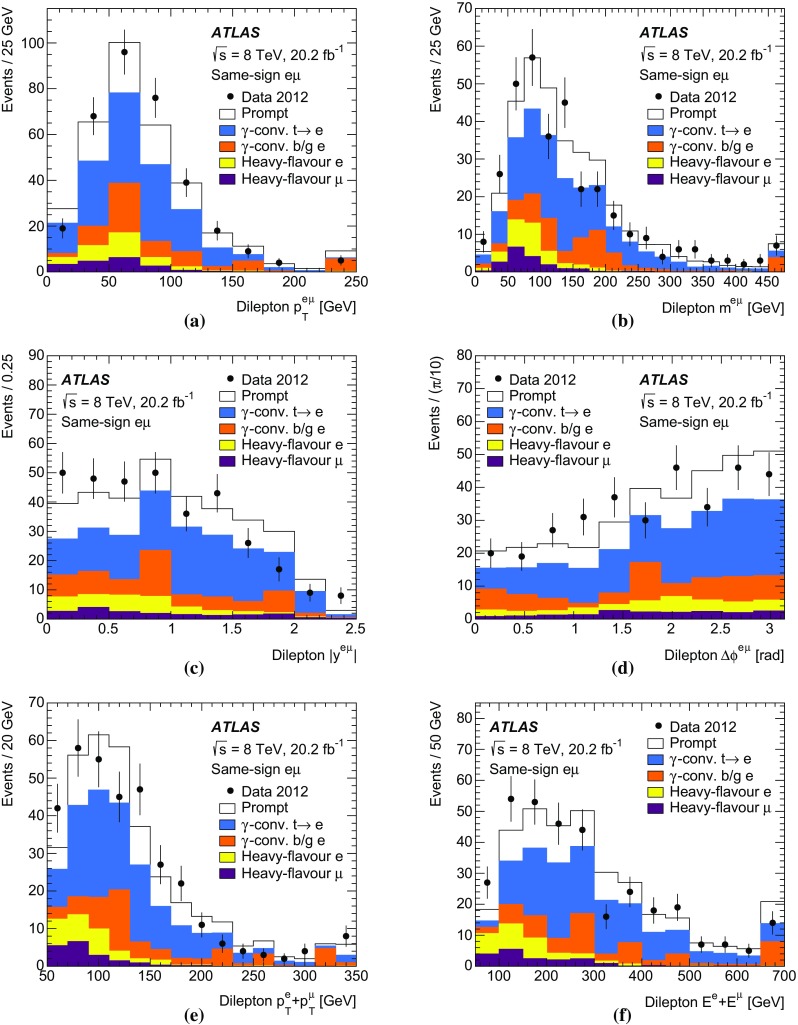
Distributions of **a** the dilepton $p_{\mathrm T}^{e\mu }$, **b** invariant mass $m^{e\mu }$, **c** rapidity $|y^{e\mu }|$, **d** azimuthal angle difference $\Delta \phi ^{e\mu }$, **e** lepton $p_{\text {T}}$ sum $p_{\mathrm T}^{e}+p_{\mathrm T}^{\mu }$ and **f** lepton energy sum $E^{e}+E^{\mu }$, in events with a same-sign $e\mu$ pair and at least one *b*-tagged jet. The simulation prediction is normalised to the same integrated luminosity as the data, and broken down into contributions where both leptons are prompt, or one is a misidentified lepton from a photon conversion originating from a top quark decay or from background, or from heavy-flavour decay. In the $p_{\mathrm T}^{e\mu }$, $m^{e\mu }$, $p_{\mathrm T}^{e}+p_{\mathrm T}^{\mu }$ and $E^{e}+E^{\mu }$ distributions, the last bin includes the overflows

The background from events with one real and one misidentified lepton was estimated using a combination of data and simulation in control regions with an electron and muon of the same charge [13]. Simulation studies showed that the samples with a same-sign $e\mu$ pair and one or two *b*-tagged jets are dominated by events with a misidentified lepton, with rates and kinematic distributions similar to those in the opposite-sign sample. The distributions of the dilepton kinematic variables for same-sign events with at least one *b*-tagged jet in data are shown in Fig. 4, and compared with the predictions from simulation. The expected contributions are shown separately for events with two prompt leptons, events where the electron candidate originates from a converted photon radiated from an electron produced in a top quark decay, events with a converted photon from other sources, and events where the electron or muon originates from the decay of a bottom or charm hadron. The analogous distributions for the electron and muon $p_{\text {T}}$ and $|\eta |$ are shown in Ref. [13]. In general, the simulation models the rates and kinematic distributions of the same-sign events well. The modelling of misidentified leptons was further tested in control samples where either the electron or muon isolation requirements were relaxed in order to enhance the contributions from heavy-flavour decays, and similar levels of agreement were observed.

The contributions $N_j^{i,\mathrm {mis{-}id}}$ of events with misidentified leptons to the opposite-sign samples with $j=1$, 2 *b*-tagged jets were estimated in each bin *i* of each distribution using

4 $$\begin{aligned} \begin{array}{rll} {N_j^{i,\mathrm {mis{-}id}}}&{} = &{} R^i_j ({N_j^{i,\mathrm {data,SS}}}-{N_j^{i,\mathrm {prompt,SS}}}) , \\ *[1mm] R^i_j &{} = &{} \frac{{N_j^{i,\mathrm {mis{-}id,OS}}}}{{N_j^{i,\mathrm {mis{-}id,SS}}}}, \end{array} \end{aligned}$$

where $N_j^{i,\mathrm {data,SS}}$ is the number of observed same-sign events in bin *i* with *j* *b*-tagged jets, $N_j^{i,\mathrm {prompt,SS}}$ is the estimated number of events in this bin with two prompt leptons, and $R^i_j$ is the ratio of the number of opposite- to same-sign events with misidentified leptons in bin *i* with *j* *b*-tagged jets. This formalism uses the observed data same-sign event rate in each bin to predict the corresponding opposite-sign contribution from misidentified leptons. It relies on simulation to predict the ratios of opposite- to same-sign rates and the prompt same-sign contribution, but not the absolute normalisation of misidentified leptons. The prompt-lepton contribution in Eq. (4) comes mainly from semileptonic $t\bar{t}$ events with an additional *W* or *Z* boson, diboson events with two same-sign leptons, and $t\bar{t} \rightarrow e\mu \nu \bar{\nu }b\bar{b}$ events where the electron charge was misreconstructed. These components were evaluated directly from simulation in each bin (*i*, *j*), and an uncertainty of ± 50% was assigned [13]. The values of $R^i_j$ were taken from simulation, separately for each differential distribution and $j=1$ and 2 *b*-tagged jets, and averaged over several consecutive bins *i* in order to reduce statistical fluctuations. The values of $R^i_1$ range from 0.8 to 1.5, and $R^i_2$ from 1.2 to 2.0, as the predicted background composition changes across the kinematic distributions. As in Ref. [13], uncertainties of ± 0.25 and ± 0.5 were assigned to $R^i_1$ and $R^i_2$, based on the variation of $R^i_j$ for different components of the misidentified lepton background, and taken to be correlated across all bins (*i*, *j*).

**Fig. 5:**
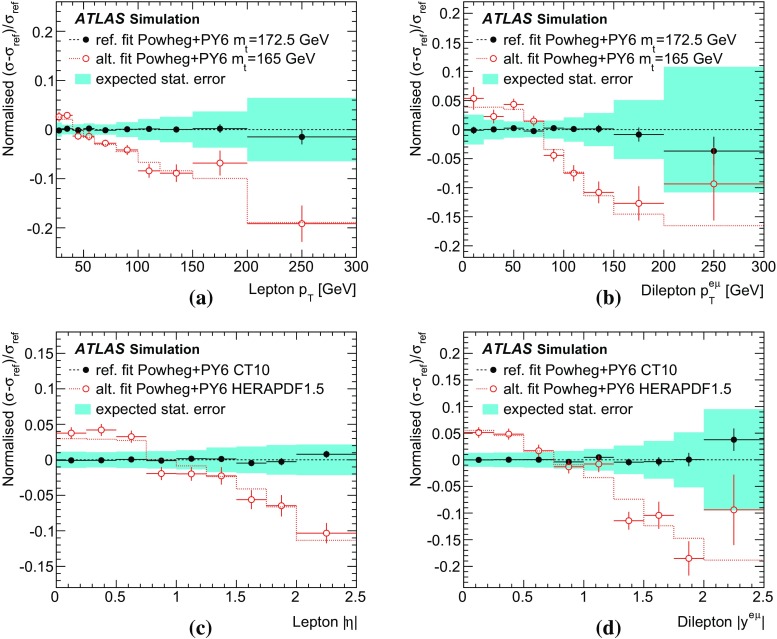
Results of pseudo-experiment studies on simulated events for the extraction of the normalised differential cross-section distributions for **a** $p_{\mathrm T}^{\ell }$, **b** $p_{\mathrm T}^{e\mu }$, **c** $|\eta ^{\ell }|$ and **d** $|y^{e\mu }|$, shown as relative deviations $(\sigma -\sigma _{\mathrm {ref}})/\sigma _{\mathrm {ref}}$ from the reference cross-section values in the baseline Powheg+Pythia6 CT10 sample with ${m_t}=172.5$ GeV. The black points show the mean deviations from the reference when fitting pseudo-data samples generated with the baseline simulation sample, with error bars indicating the uncertainties due to the limited number of simulated events. The cyan bands indicate the expected statistical uncertainties for a single sample corresponding to the data integrated luminosity. The open red points show the mean deviations from the reference values when fitting pseudo-experiments generated from alternative simulation samples with ${m_t}=165$ GeV (**a**, **b**) or with the HERAPDF 1.5 PDF (**c**, **d**), with error bars due to the limited size of these alternative samples. The red dotted lines show the true deviations from the reference in the alternative samples

### Validation of the analysis procedure

The method for the differential cross-section determination was tested on simulated events in order to check for biases and determine the expected statistical uncertainties. Pseudo-data samples corresponding to the data integrated luminosity were produced by varying the event counts $N^i_1$ and $N^i_2$ in each bin *i* independently, according to Poisson distributions with mean values predicted from a chosen $t\bar{t}$ simulation sample plus non-$t\bar{t}$ backgrounds. The tagging equations Eq. (1) were then solved for each pseudo-experiment using the values of $G^i_{e\mu }$, $C^i_b$, $N_1^{i,\mathrm {bkg}}$ and $N_2^{i,\mathrm {bkg}}$ calculated with the baseline simulation samples. An initial set of 1000 pseudo-experiments was performed using the baseline simulation sample as a reference, and the mean and RMS width of the deviations of the result in each bin from the reference values were used to validate the analysis procedure. The black points in Fig. 5 show the mean deviation of the results (averaged over all pseudo-experiments) for four of the measured normalised distributions, with error bars corresponding to the uncertainty in the mean due to the finite size of the simulation samples (about 17 times the data integrated luminosity). The residual biases of the mean deviations away from the reference are compatible with zero and in all cases much smaller than the expected statistical uncertainties in data, measured by the RMS widths and shown by the cyan bands. Similar results were obtained for the other normalised differential cross-section distributions, and for the absolute distributions. The pull distributions (i.e. the distributions of deviations divided by the estimated statistical uncertainty from each pseudo-experiment) were also found to have widths within a few percent of unity. The $\chi ^2$ values for the compatibility of each measured distribution with the reference were also calculated for each pseudo-experiment and the distribution of the corresponding *p*-values across all pseudo-experiments was found to be uniform between zero and one. These tests confirm that the analysis procedure is unbiased and correctly estimates the statistical uncertainties in each bin of each distribution.

Additional pseudo-experiments were performed to test the ability of the analysis procedure to reconstruct distributions different from the reference, taking the values of $G^i_{e\mu }$, $C^i_b$, $N_1^{i,\mathrm {bkg}}$ and $N_2^{i,\mathrm {bkg}}$ from the baseline samples. Tests were conducted using simulated Powheg + Pythia6 and MC@NLO + Herwig $t\bar{t}$ samples with different top mass values, a Powheg + Pythia6 sample generated using the HERAPDF 1.5 [84, 85] PDF set instead of CT10, and a Powheg + Pythia6 sample reweighted to reproduce the top quark $p_{\text {T}}$ distribution calculated at NNLO from Ref. [25]. In all cases, the analysis procedure recovered the true distributions from the alternative samples within the statistical precision of the test, demonstrating the adequacy of the bin-by-bin correction procedure without the need for iteration or a more sophisticated matrix-based unfolding technique. Some examples are shown by the red points and dotted lines in Fig. 5, for an alternative sample with ${m_t}=165$ GeV for $p_{\mathrm T}^{\ell }$ and $p_{\mathrm T}^{e\mu }$, and for HERAPDF 1.5 for $|\eta ^{\ell }|$ and $|y^{e\mu }|$, both simulation samples having about twice the statistics of the data. These figures also demonstrate the sensitivities of some of the measured distributions to $m_t$ and different PDFs.

For the single-lepton distributions $p_{\mathrm T}^{\ell }$ and $|\eta ^{\ell }|$, which have two entries per event, the formalism of Eq. (1) and the pseudo-experiments generated by fluctuating each bin independently do not take into account correlations between the kinematics of the electron and muon in each event. This effect was checked by generating pseudo-data samples corresponding to the data integrated luminosity from individual simulated events, taken at random from a large $t\bar{t}$ sample combining both full and fast simulation and corresponding to about 70 times the data integrated luminosity. The effect of neglecting the electron-muon correlations within an event was found to correspond to at most a 2% fractional overestimate of the absolute and 2% fractional underestimate of the normalised cross-section uncertainties. Hence, no corresponding corrections to the statistical uncertainties were made.

## Systematic uncertainties

**Fig. 6:**
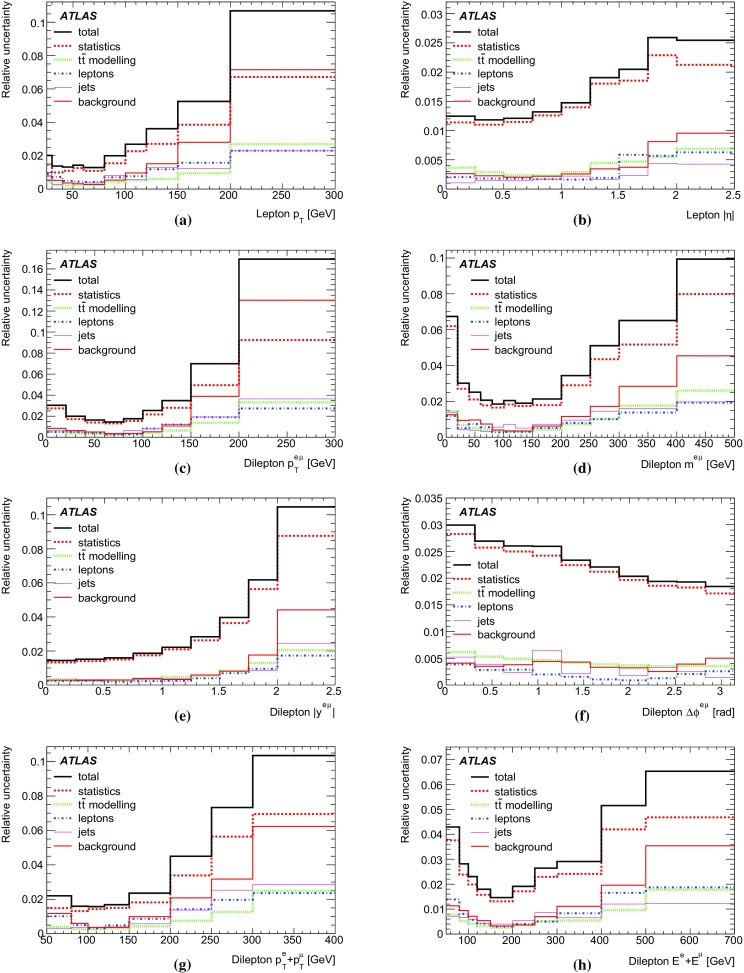
Relative uncertainties on the measured normalised differential cross-sections coming from data statistics, $t\bar{t}$ modelling, leptons, jets and background, as a function of each lepton or dilepton differential variable. The total uncertainty is shown by the black lines, and also includes small contributions from the integrated luminosity and LHC beam energy uncertainties

Systematic uncertainties in the measured cross-sections arise from uncertainties in the values of the input quantities $G^i_{e\mu }$, $C^i_b$, $N_1^{i,\mathrm {bkg}}$, $N_2^{i,\mathrm {bkg}}$ and *L* used in Eq. (1). Each source of systematic uncertainty was evaluated by coherently changing the values of all relevant input quantities and re-solving Eq. (1), thus taking into account correlations of the uncertainties in e.g. $G^i_{e\mu }$ and $C^i_b$. The uncertainties are divided into five groups ($t\bar{t}$ modelling, leptons, jets/*b*-tagging, background and luminosity/beam energy uncertainties) and are discussed in Sects. 5.1–5.5. The resulting relative uncertainties in each measured differential cross-section value are shown in the results Tables 3, 4, 5 and 6, and the grouped systematic uncertainties for the normalised differential cross-sections are shown in Fig. 6, together with the statistical and total uncertainties.

### $t\bar{t}$ modelling

The uncertainties in $G^i_{e\mu }$ and $C^i_b$ (and $f^i_{\bar{\tau }}$ for the $\tau$-corrected cross-sections) were evaluated using the various alternative $t\bar{t}$ simulation samples detailed in Sect. 2.

- ${\varvec{t}}\bar{{\varvec{t}}}$ **generator:** Event generator uncertainties were evaluated by comparing the baseline Powheg + Pythia6 $t\bar{t}$ sample (with ${h_{\mathrm {damp}}}={m_t}$) with alternative samples generated with MC@NLO interfaced to Herwig (thus changing both the NLO hard-scattering event generator and the parton shower, hadronisation and underlying event model), and with the LO multi-leg event generator Alpgen, also interfaced to Herwig. The bin-by-bin shifts in $G^i_{e\mu }$ and $C^i_b$ were fitted with polynomial functions in order to reduce statistical fluctuations caused by the limited size of the simulated samples, and the larger of the differences between the baseline and the two alternative samples was taken in each bin to define the generator uncertainty. As also found in the inclusive cross-section analysis [13], a substantial part of the differences in $G^i_{e\mu }$ in the various samples arises from differences in the hadronic activity close to the leptons, which affects the efficiency of the lepton isolation requirements. These efficiencies were therefore measured in situ in $t\bar{t}$ events selected in data as discussed in Sect. 5.2 below, and the simulation uncertainties on $G^i_{e\mu }$ evaluated by considering the lepton reconstruction, identification and lepton-jet overlap requirements only. The resulting uncertainties on $G^i_{e\mu }$ are typically 0.5–1% in most regions of the phase space, varying only slightly as a function of the lepton and dilepton kinematics. The same procedure was used to evaluate uncertainties in $C^i_b$, and the predictions of the three simulation samples were found to agree at the 0.5–1% level, giving similar predictions for the variations of $C^i_b$ across the bins of the various measured distributions. Alternative $t\bar{t}$ samples generated with Powheg + Pythia6 and Powheg + Herwig (both with ${h_{\mathrm {damp}}}=\infty$) were also considered, but the resulting differences in $G^i_{e\mu }$ and $C^i_b$ were found to be significantly less than those from the comparisons with MC@NLO + Herwig and thus no additional uncertainty was assigned. Variations in the predictions of $f^i_{\bar{\tau }}$ from the three $t\bar{t}$ samples were found to be at the 0.2% level, and were also taken into account for the $\tau$-corrected cross-section results.
- **Initial/final-state radiation:** The effects on $G^i_{e\mu }$  $C^i_b$ and $f^i_{\bar{\tau }}$ of uncertainties in the modelling of additional radiation in $t\bar{t}$ events were assessed as half the difference between Powheg + Pythia6 samples tuned to span the uncertainties in jet activity measured in $\sqrt{s}=7$ TeV ATLAS data [26, 55, 64], as discussed in Sect. 2. The uncertainties were taken as half the difference between the upward and downward variations, and were substantially reduced by measuring the lepton isolation efficiencies from data, in the same way as for the $t\bar{t}$ generator uncertainties discussed above.
- **Parton distribution functions:** The uncertainties in $G^i_{e\mu }$ due to limited knowledge of the proton PDFs were evaluated using the error sets of the CT10 [10], MSTW 2008 68% CL [8] and NNPDF 2.3 [12] NLO PDF sets, by reweighting the MC@NLO + Herwig $t\bar{t}$ sample based on the *x* and $Q^2$ values of the partons participating in the hard scattering in each event. The final uncertainty in each bin was calculated as half the envelope encompassing the predictions from all three PDF sets and their associated uncertainties, following the PDF4LHC prescription [7]. The resulting uncertainties on $G^i_{e\mu }$ are typically around 0.3% except at the high ends of the distributions, and were taken to be fully correlated across all bins.
- **Top quark mass:** The values of $G^i_{e\mu }$ and the predicted levels of *Wt* background depend weakly on the assumed value of $m_t$. These effects were evaluated with $t\bar{t}$ and *Wt* samples simulated with $m_t$ values of 170 and 175 GeV, and scaled to a nominal $\pm 1$ GeV mass variation. The resulting effects are at the level of 0.1–0.2% on $G^i_{e\mu }$, and are partially cancelled by the variations in the *Wt* background, whose cross-section decreases with increasing $m_t$. The residual uncertainties are typically around 0.1% for the absolute cross-sections except at the extreme ends of the distributions, and smaller for the normalised cross-sections.

The total $t\bar{t}$ modelling uncertainties in the normalised differential cross-sections also include the small uncertainties on $G^i_{e\mu }$ and $C^i_b$ from the limited size of the simulated $t\bar{t}$ samples, and are shown by the green lines in Fig. 6. They are typically dominated by the $t\bar{t}$ event generator comparisons.

### Lepton identification and measurement

Uncertainties in the modelling of the detector response to electrons and muons affect both $G^i_{e\mu }$ and the background estimates, with the largest uncertainties in the cross-section measurements coming via the former.

- **Lepton identification:** The modelling of the electron and muon identification efficiencies, and the rate of electron charge misidentification, were studied using $Z\rightarrow ee/\mu \mu$, $J/\psi \rightarrow ee/\mu \mu$ and $W\rightarrow e\nu$ events in data and simulation [76, 77], taking into account the systematic correlations across different regions of the lepton $p_{\text {T}}$ and $\eta$ spectrum. The uncertainties in $G^i_{e\mu }$ are typically below 0.5% for electron and below 0.3% for muon efficiencies, with significant cancellations in the normalised differential cross-sections.
- **Lepton scales and resolution:** The electron and muon energy/momentum scales and resolutions were determined using $Z\rightarrow ee/\mu \mu$, $Z\rightarrow (ee/\mu \mu )\gamma$, $J/\psi \rightarrow ee/\mu \mu$ and $\Upsilon \rightarrow \mu \mu$ decays [77, 86]. The largest uncertainty comes from the limited knowledge of the electron energy scale, which gives uncertainties varying from 0.2% to over 2% for the bins involving the highest energy electrons. The muon momentum scale uncertainties are small in comparison.
- **Lepton isolation:** Building on the studies described in Ref. [13], the efficiencies of the lepton isolation requirements were measured in data, using the fractions of selected opposite-sign $e\mu$ events with at least one *b*-tagged jet where either the electron or the muon fails the isolation requirement. After correcting for the contamination from events with a misidentified lepton, these fractions give the inefficiency of the isolation requirements on signal $t\bar{t}$ events. The misidentified lepton backgrounds were measured both by using the same-sign $e\mu$ control samples discussed in Sect. 4.2 above, and by using the distributions of lepton impact parameter significance $|d_0|/\sigma _{d_0}$, where $d_0$ is the distance of closest approach of the lepton track to the event primary vertex in the transverse plane, and $\sigma _{d_0}$ its uncertainty. The isolation inefficiencies were measured as functions of lepton $p_{\text {T}}$ separately for the barrel ($|\eta |<1.5$) and endcap regions of the detector. Consistent results were obtained using both misidentified lepton estimation methods, and showed that the baseline Powheg + Pythia6 $t\bar{t}$ simulation sample overestimates the efficiencies of the lepton isolation requirements by up to 1% for electrons with $p_{\text {T}}$ in the range 40–80 GeV, and by up to 2% for muons at low $p_{\text {T}}$, decreasing rapidly to less than 0.5% for 40 GeV. The values of $G^i_{e\mu }$ from the baseline simulation were corrected for these $p_{\text {T}}$-dependent shifts using a reweighting technique. The corresponding uncertainties are dominated by those on the misidentified lepton subtraction (including a comparison of the same-sign and $|d_0|/\sigma _{d_0}$-based methods) and amount to typically 0.5–1% for electrons and 0.2–0.5% for muons. The effect on the normalised cross-sections is about half that on the absolute measurements, taking into account systematic correlations across lepton $p_{\text {T}}$ and $|\eta |$ bins.
- **Lepton trigger:** The efficiencies of the single-lepton triggers were measured in data using $Z\rightarrow ee/\mu \mu$ events [87]. Since only one lepton trigger was required to accept the $e\mu$ event, the trigger efficiency with respect to the offline event selection is about 99%, with a residual uncertainty of less than 0.2%.

The lepton-related uncertainties are shown by the blue dot-dashed lines in Fig. 6, and the largest uncertainties typically come from the electron energy scale and electron isolation uncertainties.

### Jet measurement and *b*-tagging

Uncertainties in the selection and *b*-tagging of jets affect the background estimates $N_1^{i,\mathrm {bkg}}$ and $N_2^{i,\mathrm {bkg}}$, and to a lesser extent, the correlation $C^i_b$. The jet uncertainties also have a very small effect on $G^i_{e\mu }$, through the requirement that leptons be separated from selected jets by $\Delta R>0.4$.

- **Jet-related uncertainties:** The jet energy scale was varied according to the uncertainties derived from simulation and in situ calibration measurements [81], using a model with 22 orthogonal uncertainty components describing the evolution with jet $p_{\text {T}}$ and $|\eta |$. The effects of residual uncertainties in the modelling of the jet energy resolution [88] were assessed by smearing jet energies in simulation. The jet reconstruction efficiency was measured in data using track-based jets, and the effect of residual uncertainties assessed in simulation by randomly discarding jets. The modelling of the pileup rejection requirement applied to jets was studied using $Z\rightarrow ee/\mu \mu$+jets events [82].
- ${\varvec{b}}$ **-tagging uncertainties:** The efficiencies for *b*-tagging jets in $t\bar{t}$ signal events were extracted from the data, but simulation was used to predict the numbers of *b*-tagged jets in the *Wt* single top and diboson backgrounds. The corresponding uncertainties were assessed using studies of *b*-jets containing muons, charm jets containing $D^{*+}$ mesons and inclusive jet events [83].

The jet- and *b*-tagging-related uncertainties are shown by the purple lines on Fig. 6, and are typically dominated by the effect of the jet energy scale on the level of *Wt* background.

### Background modelling

As well as the detector-related uncertainties discussed above, the background estimates depend on uncertainties in modelling the *Wt* and diboson processes taken from simulation, and uncertainties in the procedures used for estimating the *Z*+jets and misidentified lepton backgrounds from data.

- **Single top modelling:** Uncertainties in the modelling of the *Wt* background were assessed by comparing the predictions from the baseline Powheg + Pythia6 sample with those from MC@NLO + Herwig, and from two samples generated with AcerMC + Pythia6 utilising different tunes to vary the amount of additional radiation, in all cases normalising the total production cross-section to the approximate NNLO prediction based on Ref. [67]. The uncertainty in this prediction was evaluated to be 6.8%. The *Wt* background with two *b*-tagged jets is sensitive to the production of *Wt* with an additional *b*-jet, an NLO contribution which interferes with the $t\bar{t}$ final state. The corresponding uncertainty was assessed by comparing the predictions of Powheg + Pythia6 with the diagram removal and diagram subtraction schemes for handling this interference [65, 66]. The latter predicts up to 25% less *Wt* background in the one *b*-tagged and 60% less in the two *b*-tagged channels at the extreme high ends of the lepton $p_{\text {T}}$ and dilepton $p_{\mathrm T}^{e\mu }$, $m^{e\mu }$, $p_{\mathrm T}^{e}+p_{\mathrm T}^{\mu }$ and $E^{e}+E^{\mu }$ distributions, but only 1–2% and 20% differences for one and two *b*-tagged *Wt* events across the $|\eta ^{\ell }|$, $|y^{e\mu }|$ and $\Delta \phi ^{e\mu }$ distributions, similar to the differences seen for the inclusive analysis [13]. The uncertainties due to the limited size of the *Wt* simulation samples are negligible in comparison to the modelling uncertainties.
- **Diboson modelling:** The uncertainties in modelling the diboson background events (mainly *WW*) with one and two additional *b*-tagged jets were assessed by comparing the predictions from Alpgen + Herwig with those of Sherpa 1.4.3 [73] including the effects of massive *b* and *c* quarks. The resulting uncertainties in the diboson background are typically in the range 20–30%, substantially larger than the differences between recent predictions for the inclusive diboson cross-sections at NNLO in QCD [89] and the NLO predictions from MCFM used to normalise the simulated samples. The background from SM Higgs production with $H\rightarrow WW$ and $H\rightarrow \tau \tau$ is smaller than the uncertainties assigned for diboson modelling, and was neglected.
- **Z+jets extrapolation:** The backgrounds from $Z\rightarrow \tau \tau \rightarrow e\mu$ accompanied by one or two *b*-tagged jets were extrapolated from the analogous $Z\rightarrow ee/\mu \mu$ event rates, with uncertainties of 20% for one and 30% for two additional *b*-tagged jets, as discussed in Sect. 4.2.
- **Misidentified leptons:** Uncertainties in the numbers of events with misidentified leptons arise from the statistical uncertainties in the corresponding same-sign samples, together with systematic uncertainties in the opposite-to-same-sign ratios $R^i_j$ and the estimated contributions of prompt same-sign events. The total uncertainties in the measured cross-sections are typically 0.2–0.5%, except at the extreme ends of distributions where the same-sign data statistical uncertainties are larger.

The background uncertainties are shown by the solid red lines on Fig. 6, and are dominated by *Wt* modelling uncertainties, in particular from the *Wt*-$t\bar{t}$ interference at the high ends of some distributions.

### Luminosity and beam energy

Uncertainties in the integrated luminosity and LHC beam energy give rise to additional uncertainties in the differential cross-section results.

- **Luminosity:** The uncertainty in the integrated luminosity is 1.9%, derived from beam-separation scans performed in November 2012 [90]. The corresponding uncertainty in the absolute cross-section measurements is slightly larger, typically about 2.1%, as the *Wt* and diboson backgrounds were evaluated from simulation, thus becoming sensitive to the assumed integrated luminosity. The sensitivity varies with the background fractions, leaving a residual uncertainty of typically less than 0.1% in the normalised cross-section results.
- **Beam energy:** The LHC beam energy during the 2012 *pp* run was determined to be within 0.1% of the nominal value of 4 TeV per beam, based on the LHC magnetic model together with measurements of the revolution frequency difference of proton and lead-ion beams [91]. Following the approach used in Ref. [13] with an earlier less precise determination of the LHC beam energy [92], an additional uncertainty corresponding to the change in cross-sections for a 0.1% change in $\sqrt{s}$ was applied to the final results, allowing them to be interpreted as measurements at exactly $\sqrt{s}=8$ TeV. The changes in each differential cross-section bin were calculated by scaling the differences seen in Powheg + Pythia6 samples generated at $\sqrt{s}=8$ TeV and $\sqrt{s}=7$ TeV. The resulting values were cross-checked with an explicit NLO fixed-order calculation using Sherpa 2.1 [73], making use of the Applgrid framework [93] to reweight an $\sqrt{s}=8$ TeV prediction so as to change the $\sqrt{s}$ value by $\pm 0.66$% which was then rescaled to correspond to a $\sqrt{s}$ change of 0.1%. The changes in the absolute cross-sections are in the range 0.2–0.4%, and largely cancel in the normalised cross-sections.

These uncertainties are not shown separately in Fig. 6, but are included in the total uncertainties shown by the black lines, and given in Tables 3, 4, 5 and 6.

## Results

The absolute differential cross-sections were determined by solving Eq. (1) separately for each bin *i* of each lepton and dilepton differential distribution, taking the effects of systematic uncertainties into account as discussed in Sect. 5. The normalised differential cross-sections were determined from the absolute results using Eq. (2). The values of $\epsilon ^i_{b}$, i.e. the product of jet acceptance, reconstruction and *b*-tagging probabilities in each bin, were determined to be in the range 0.5–0.6, in agreement with the simulation prediction for each bin. The results were found to be stable when changing the minimum jet $p_{\text {T}}$ requirement from 25 GeV up to 55 GeV, and when using *b*-tagging working points corresponding to *b*-jet efficiencies of 60–80%. The electron and muon $p_{\text {T}}$ and $|\eta |$ distributions were also measured separately, instead of combining them into lepton distributions with two entries per event, and found to be compatible. The bin-by-bin comparison of the electron and muon $p_{\text {T}}$ ($|\eta |$) distributions has a $\chi ^2$ per degree of freedom of 10.9/9 (12.5/8), in both cases taking into account statistical and uncorrelated systematic uncertainties.

**Table 3:** Absolute and normalised differential cross-sections as functions of $p_{\mathrm T}^{\ell }$ (top) and $|\eta ^{\ell }|$ (bottom). The columns show the bin ranges, measured cross-section and total uncertainty, relative statistical uncertainty, relative systematic uncertainties in various categories (see text), total relative uncertainty, and differential cross-section corrected to remove contributions via $W\rightarrow \tau \rightarrow e/\mu$ decays. Relative uncertainties smaller than 0.05% are indicated by ‘0.0’. The last bin includes overflows where indicated by the ‘+’ sign

Absolute bin (GeV)	$\mathrm {d}\sigma /\mathrm {d}{p_{\mathrm T}^{\ell }}$ (fb/GeV)	Stat. (%)	$t\bar{t}$ mod. (%)	Lept. (%)	Jet/*b* (%)	Bkg. (%)	$L/E_\mathrm {b}$ (%)	Total (%)	$\mathrm {d}\sigma /\mathrm {d}{p_{\mathrm T}^{\ell }}$ (no $\tau$) (fb/GeV)
25–30	$154.8\pm 5.7$	1.6	1.3	1.8	0.8	1.2	2.1	3.7	$127.2\pm 4.8$
30–40	$146.1\pm 4.9$	1.1	1.2	1.5	0.8	1.0	2.1	3.3	$124.6\pm 4.2$
40–50	$118.8\pm 3.7$	1.2	1.1	1.0	1.0	0.9	2.1	3.1	$104.3\pm 3.3$
50–60	$93.5\pm 2.9$	1.4	1.0	1.0	0.8	0.9	2.1	3.1	$83.4\pm 2.6$
60–80	$60.0\pm 1.8$	1.2	0.9	0.9	0.6	0.9	2.1	3.0	$54.1\pm 1.6$
80–100	$32.4\pm 1.1$	1.6	0.8	1.1	1.4	1.1	2.1	3.5	$29.4\pm 1.0$
100–120	$16.23\pm 0.64$	2.3	0.9	1.1	1.1	1.5	2.2	3.9	$14.75\pm 0.58$
120–150	$7.61\pm 0.35$	2.7	1.1	1.4	1.5	1.9	2.2	4.6	$6.91\pm 0.32$
150–200	$2.41\pm 0.15$	3.9	1.6	1.7	1.6	3.2	2.2	6.2	$2.17\pm 0.13$
200–300+	$0.49\pm 0.06$	6.7	3.5	2.3	2.9	7.5	2.4	11.5	$0.44\pm 0.05$

Normalised bin (GeV)	$\frac{1}{\sigma }\mathrm {d}\sigma /\mathrm {d}{p_{\mathrm T}^{\ell }}$ ($10^{-2}/$GeV)	Stat. (%)	$t\bar{t}$ mod. (%)	Lept. (%)	Jet/*b* (%)	Bkg. (%)	$L/E_\mathrm {b}$ (%)	Total (%)	$\frac{1}{\sigma }\mathrm {d}\sigma /\mathrm {d}{p_{\mathrm T}^{\ell }}$ (no $\tau$) ($10^{-2}/$GeV)
25–30	$2.235\pm 0.045$	1.5	0.4	0.9	0.6	0.8	0.0	2.0	$2.090\pm 0.042$
30–40	$2.108\pm 0.029$	1.0	0.3	0.7	0.3	0.5	0.0	1.4	$2.048\pm 0.029$
40–50	$1.714\pm 0.023$	1.1	0.2	0.5	0.4	0.4	0.0	1.3	$1.714\pm 0.023$
50–60	$1.350\pm 0.019$	1.3	0.2	0.4	0.3	0.3	0.0	1.4	$1.370\pm 0.020$
60–80	$0.866\pm 0.011$	1.1	0.3	0.4	0.4	0.3	0.0	1.3	$0.890\pm 0.011$
80–100	$0.4673\pm 0.0093$	1.5	0.4	0.7	0.8	0.5	0.0	2.0	$0.4831\pm 0.0096$
100–120	$0.2343\pm 0.0063$	2.3	0.5	0.8	0.6	1.0	0.0	2.7	$0.2424\pm 0.0065$
120–150	$0.1098\pm 0.0040$	2.7	0.6	1.2	1.3	1.5	0.1	3.6	$0.1135\pm 0.0041$
150–200	$0.0348\pm 0.0018$	3.9	0.9	1.6	1.2	2.8	0.1	5.3	$0.0357\pm 0.0019$
200–300+	$0.0070\pm 0.0007$	6.7	2.7	2.3	2.3	7.2	0.3	10.7	$0.0072\pm 0.0008$

Absolute bin (unit $\eta$)	$\mathrm {d}\sigma /\mathrm {d}{|\eta ^{\ell }|}$ (fb/unit $\eta$)	Stat. (%)	$t\bar{t}$ mod. (%)	Lept. (%)	Jet/*b* (%)	Bkg. (%)	$L/E_\mathrm {b}$ (%)	Total (%)	$\mathrm {d}\sigma /\mathrm {d}{|\eta ^{\ell }|}$ (no $\tau$) (fb/unit $\eta$)
0.00–0.25	$4590\pm 140$	1.2	1.0	1.0	0.9	0.9	2.1	3.1	$4030\pm 130$
0.25–0.50	$4440\pm 140$	1.2	1.0	1.0	0.9	0.9	2.1	3.1	$3900\pm 120$
0.50–0.75	$4230\pm 130$	1.2	1.0	1.0	0.9	0.9	2.1	3.1	$3710\pm 120$
0.75–1.00	$3660\pm 110$	1.3	1.0	1.0	0.8	1.0	2.1	3.1	$3210\pm 100$
1.00–1.25	$3100\pm 100$	1.5	1.0	1.0	0.9	1.0	2.1	3.3	$2722\pm 89$
1.25–1.50	$2470\pm 87$	1.9	1.1	1.0	0.9	1.0	2.1	3.5	$2173\pm 77$
1.50–1.75	$2035\pm 73$	1.9	1.1	1.4	0.7	1.0	2.1	3.6	$1793\pm 65$
1.75–2.00	$1431\pm 57$	2.4	1.2	1.4	0.6	1.4	2.1	4.0	$1263\pm 50$
2.00–2.50	$844\pm 34$	2.2	1.3	1.4	0.7	1.4	2.1	4.0	$749\pm 30$

Normalised bin (unit $\eta$)	$\frac{1}{\sigma }\mathrm {d}\sigma /\mathrm {d}{|\eta ^{\ell }|}$ ($10^{-1}/$unit $\eta$)	Stat. (%)	$t\bar{t}$ mod. (%)	Lept. (%)	Jet/*b* (%)	Bkg. (%)	$L/E_\mathrm {b}$ (%)	Total (%)	$\frac{1}{\sigma }\mathrm {d}\sigma /\mathrm {d}{|\eta ^{\ell }|}$ (no $\tau$) ($10^{-1}/$unit $\eta$)
0.00–0.25	$6.646\pm 0.083$	1.1	0.4	0.2	0.1	0.3	0.0	1.2	$6.632\pm 0.083$
0.25–0.50	$6.428\pm 0.076$	1.1	0.3	0.2	0.1	0.2	0.0	1.2	$6.416\pm 0.076$
0.50–0.75	$6.117\pm 0.074$	1.1	0.2	0.2	0.1	0.2	0.0	1.2	$6.103\pm 0.074$
0.75–1.00	$5.297\pm 0.070$	1.3	0.2	0.2	0.2	0.2	0.0	1.3	$5.286\pm 0.070$
1.00–1.25	$4.482\pm 0.066$	1.4	0.3	0.2	0.2	0.3	0.0	1.5	$4.484\pm 0.066$
1.25–1.50	$3.574\pm 0.068$	1.8	0.4	0.2	0.2	0.3	0.0	1.9	$3.579\pm 0.068$
1.50–1.75	$2.944\pm 0.060$	1.9	0.5	0.6	0.2	0.4	0.0	2.0	$2.954\pm 0.061$
1.75–2.00	$2.070\pm 0.054$	2.3	0.6	0.6	0.4	0.8	0.0	2.6	$2.080\pm 0.054$
2.00–2.50	$1.221\pm 0.031$	2.1	0.7	0.6	0.4	1.0	0.0	2.5	$1.233\pm 0.031$

**Table 4:** Absolute and normalised differential cross-sections as functions of $p_{\mathrm T}^{e\mu }$ (top) and $m^{e\mu }$ (bottom). The columns show the bin ranges, measured cross-section and total uncertainty, relative statistical uncertainty, relative systematic uncertainties in various categories (see text), total relative uncertainty, and differential cross-section corrected to remove contributions via $W\rightarrow \tau \rightarrow e/\mu$ decays. Relative uncertainties smaller than 0.05% are indicated by ‘0.0’. The last bin includes overflows where indicated by the ‘+’ sign

Absolute bin (GeV)	$\mathrm {d}\sigma /\mathrm {d}{p_{\mathrm T}^{e\mu }}$ (fb/GeV)	Stat. (%)	$t\bar{t}$ mod. (%)	Lept. (%)	Jet/*b* (%)	Bkg. (%)	$L/E_\mathrm {b}$ (%)	Total (%)	$\mathrm {d}\sigma /\mathrm {d}{p_{\mathrm T}^{e\mu }}$ (no $\tau$) (fb/GeV)
0–20	$11.50\pm 0.49$	2.8	1.2	1.2	1.1	1.2	2.1	4.2	$9.62\pm 0.41$
20–40	$26.72\pm 0.94$	1.9	1.0	1.2	1.0	1.0	2.1	3.5	$22.62\pm 0.81$
40–60	$35.9\pm 1.2$	1.6	0.9	1.2	1.1	1.0	2.1	3.4	$30.6\pm 1.0$
60–80	$39.0\pm 1.3$	1.5	0.9	1.1	0.9	0.9	2.1	3.2	$34.4\pm 1.1$
80–100	$29.19\pm 0.96$	1.7	1.0	1.0	0.8	1.0	2.1	3.3	$26.48\pm 0.88$
100–120	$16.38\pm 0.65$	2.3	1.2	1.3	1.3	1.1	2.1	3.9	$15.11\pm 0.60$
120–150	$6.53\pm 0.30$	2.9	1.4	1.5	1.2	1.6	2.2	4.6	$6.06\pm 0.28$
150–200	$1.39\pm 0.11$	5.0	2.2	2.1	2.2	4.2	2.3	7.9	$1.27\pm 0.10$
200–300+	$0.23\pm 0.04$	9.3	4.2	2.9	4.1	13.2	2.5	17.6	$0.20\pm 0.04$

Normalised bin (GeV)	$\frac{1}{\sigma }\mathrm {d}\sigma /\mathrm {d}{p_{\mathrm T}^{e\mu }}$ ($10^{-2}/$GeV)	Stat. (%)	$t\bar{t}$ mod. (%)	Lept. (%)	Jet/*b* (%)	Bkg. (%)	$L/E_\mathrm {b}$ (%)	Total (%)	$\frac{1}{\sigma }\mathrm {d}\sigma /\mathrm {d}{p_{\mathrm T}^{e\mu }}$ (no $\tau$) ($10^{-2}/$GeV)
0–20	$0.332\pm 0.010$	2.7	0.5	0.5	0.7	0.8	0.0	3.0	$0.316\pm 0.010$
20–40	$0.772\pm 0.015$	1.7	0.4	0.5	0.5	0.6	0.0	2.0	$0.743\pm 0.015$
40–60	$1.036\pm 0.017$	1.4	0.3	0.4	0.5	0.5	0.0	1.6	$1.006\pm 0.017$
60–80	$1.127\pm 0.016$	1.3	0.3	0.3	0.4	0.3	0.0	1.5	$1.130\pm 0.016$
80–100	$0.843\pm 0.015$	1.5	0.3	0.3	0.6	0.4	0.0	1.8	$0.870\pm 0.015$
100–120	$0.473\pm 0.012$	2.2	0.4	0.8	0.8	0.5	0.0	2.5	$0.497\pm 0.013$
120–150	$0.1886\pm 0.0066$	2.8	0.6	1.2	1.0	1.2	0.0	3.5	$0.1993\pm 0.0069$
150–200	$0.0402\pm 0.0028$	4.9	1.4	1.9	1.9	3.9	0.2	7.0	$0.0419\pm 0.0029$
200–300+	$0.0066\pm 0.0011$	9.2	3.3	2.7	3.6	13.0	0.4	16.9	$0.0067\pm 0.0011$

Absolute bin (GeV)	$\mathrm {d}\sigma /\mathrm {d}{m^{e\mu }}$ (fb/GeV)	Stat. (%)	$t\bar{t}$ mod. (%)	Lept. (%)	Jet/*b* (%)	Bkg. (%)	$L/E_\mathrm {b}$ (%)	Total (%)	$\mathrm {d}\sigma /\mathrm {d}{m^{e\mu }}$ (no $\tau$) (fb/GeV)
0–20	$3.37\pm 0.25$	6.3	2.0	1.9	1.3	1.3	2.1	7.4	$2.97\pm 0.22$
20–40	$10.94\pm 0.47$	2.8	1.5	1.4	0.9	1.2	2.1	4.3	$9.61\pm 0.41$
40–60	$17.66\pm 0.70$	2.2	1.4	1.5	0.7	1.3	2.1	4.0	$15.29\pm 0.61$
60–80	$23.98\pm 0.89$	1.9	1.3	1.4	0.8	1.2	2.1	3.7	$20.54\pm 0.76$
80–100	$26.00\pm 0.90$	1.8	1.2	1.1	0.8	0.9	2.1	3.4	$22.42\pm 0.78$
100–120	$23.03\pm 0.83$	2.0	1.1	1.0	1.2	0.9	2.1	3.6	$20.07\pm 0.73$
120–150	$16.71\pm 0.57$	1.9	1.0	0.9	1.0	1.0	2.1	3.4	$14.72\pm 0.51$
150–200	$9.38\pm 0.34$	1.9	0.8	0.9	1.3	1.2	2.1	3.6	$8.41\pm 0.30$
200–250	$4.09\pm 0.18$	3.0	0.8	1.1	1.1	1.6	2.1	4.4	$3.73\pm 0.16$
250–300	$1.95\pm 0.11$	4.4	1.0	1.2	1.4	2.1	2.2	5.7	$1.80\pm 0.10$
300–400	$0.66\pm 0.05$	5.2	1.3	1.5	2.0	3.1	2.2	7.1	$0.62\pm 0.04$
400–500+	$0.26\pm 0.03$	8.0	2.2	2.1	2.1	4.8	2.2	10.3	$0.25\pm 0.03$

Normalised bin (GeV)	$\frac{1}{\sigma }\mathrm {d}\sigma /\mathrm {d}{m^{e\mu }}$ ($10^{-3}/$GeV)	Stat. (%)	$t\bar{t}$ mod. (%)	Lept. (%)	Jet/*b* (%)	Bkg. (%)	$L/E_\mathrm {b}$ (%)	Total	$\frac{1}{\sigma }\mathrm {d}\sigma /\mathrm {d}{m^{e\mu }}$ (no $\tau$)
			(%)	(%)	(%)	(%)	(%)	(%)	[$10^{-3}/$GeV]
0–20	$0.973\pm 0.066$	6.2	1.4	1.2	1.4	1.3	0.0	6.7	$0.977\pm 0.066$
20–40	$3.157\pm 0.095$	2.7	0.7	0.5	0.4	0.9	0.0	3.0	$3.156\pm 0.095$
40–60	$5.10\pm 0.13$	2.1	0.5	0.7	0.4	1.0	0.0	2.5	$5.02\pm 0.13$
60–80	$6.92\pm 0.14$	1.8	0.4	0.6	0.3	0.7	0.0	2.1	$6.75\pm 0.14$
80–100	$7.51\pm 0.14$	1.7	0.3	0.3	0.6	0.4	0.0	1.8	$7.37\pm 0.14$
100–120	$6.65\pm 0.14$	1.8	0.3	0.3	0.7	0.3	0.0	2.0	$6.60\pm 0.14$
120–150	$4.823\pm 0.092$	1.7	0.3	0.3	0.5	0.3	0.0	1.9	$4.839\pm 0.092$
150–200	$2.707\pm 0.058$	1.8	0.4	0.5	0.7	0.6	0.0	2.1	$2.763\pm 0.059$
200–250	$1.180\pm 0.041$	2.9	0.7	0.8	1.0	1.2	0.0	3.4	$1.224\pm 0.042$
250–300	$0.563\pm 0.029$	4.4	1.0	1.0	1.4	1.7	0.0	5.1	$0.590\pm 0.030$
300–400	$0.191\pm 0.012$	5.2	1.8	1.4	1.6	2.8	0.1	6.5	$0.203\pm 0.013$
400–500+	$0.0763\pm 0.0076$	8.0	2.6	1.9	2.0	4.5	0.1	9.9	$0.0820\pm 0.0081$

**Table 5:** Absolute and normalised differential cross-sections as functions of $|y^{e\mu }|$ (top) and $\Delta \phi ^{e\mu }$ (bottom). The columns show the bin ranges, measured cross-section and total uncertainty, relative statistical uncertainty, relative systematic uncertainties in various categories (see text), total relative uncertainty, and differential cross-section corrected to remove contributions via $W\rightarrow \tau \rightarrow e/\mu$ decays. Relative uncertainties smaller than 0.05% are indicated by ‘0.0’. The bin boundaries for $\Delta \phi ^{e\mu }$ correspond to exact multiples of $\pi /10$ but are quoted to two decimal places

Absolute bin (unit *y*)	$\mathrm {d}\sigma /\mathrm {d}{|y^{e\mu }|}$ (fb/unit *y*)	Stat. (%)	$t\bar{t}$ mod. (%)	Lept. (%)	Jet/*b* (%)	Bkg. (%)	$L/E_\mathrm {b}$ (%)	Total (%)	$\mathrm {d}\sigma /\mathrm {d}{|y^{e\mu }|}$ (no $\tau$) (fb/unit *y*)
0.00–0.25	$3007\pm 95$	1.5	0.9	1.0	1.0	0.8	2.1	3.2	$2639\pm 84$
0.25–0.50	$2681\pm 86$	1.5	0.9	1.0	0.8	0.9	2.1	3.2	$2353\pm 76$
0.50–0.75	$2419\pm 80$	1.6	1.0	1.0	1.0	1.0	2.1	3.3	$2123\pm 71$
0.75–1.00	$2026\pm 71$	1.9	1.1	1.1	0.8	1.1	2.1	3.5	$1780\pm 63$
1.00–1.25	$1536\pm 57$	2.2	1.2	1.1	0.8	1.0	2.1	3.7	$1351\pm 50$
1.25–1.50	$1038\pm 43$	2.7	1.3	1.3	0.7	1.2	2.1	4.2	$912\pm 38$
1.50–1.75	$637\pm 33$	3.7	1.6	1.6	1.3	1.3	2.2	5.2	$561\pm 29$
1.75–2.00	$321\pm 23$	5.7	2.0	1.8	1.2	2.1	2.2	7.1	$283\pm 20$
2.00–2.50	$69.1\pm 7.7$	8.8	2.7	2.3	2.3	4.7	2.2	11.1	$61.3\pm 6.8$

Normalised bin (unit *y*)	$\frac{1}{\sigma }\mathrm {d}\sigma /\mathrm {d}{|y^{e\mu }|}$ ($10^{-1}/$unit *y*)	Stat. (%)	$t\bar{t}$ mod. (%)	Lept. (%)	Jet/*b* (%)	Bkg. (%)	$L/E_\mathrm {b}$ (%)	Total (%)	$\frac{1}{\sigma }\mathrm {d}\sigma /\mathrm {d}{|y^{e\mu }|}$ (no $\tau$) ($10^{-1}/$unit *y*)
0.00–0.25	$8.71\pm 0.13$	1.3	0.3	0.2	0.3	0.3	0.0	1.4	$8.71\pm 0.13$
0.25–0.50	$7.77\pm 0.12$	1.4	0.3	0.2	0.3	0.3	0.0	1.5	$7.76\pm 0.12$
0.50–0.75	$7.01\pm 0.11$	1.5	0.3	0.2	0.3	0.3	0.0	1.6	$7.00\pm 0.11$
0.75–1.00	$5.87\pm 0.11$	1.7	0.4	0.2	0.3	0.4	0.0	1.9	$5.87\pm 0.11$
1.00–1.25	$4.451\pm 0.099$	2.1	0.5	0.2	0.4	0.3	0.0	2.2	$4.458\pm 0.099$
1.25–1.50	$3.009\pm 0.085$	2.6	0.6	0.4	0.6	0.6	0.0	2.8	$3.009\pm 0.085$
1.50–1.75	$1.846\pm 0.073$	3.6	0.8	0.7	0.8	0.8	0.0	4.0	$1.850\pm 0.073$
1.75–2.00	$0.930\pm 0.057$	5.6	1.3	1.0	0.9	1.8	0.1	6.2	$0.935\pm 0.058$
2.00–2.50	$0.200\pm 0.021$	8.8	2.1	1.7	2.5	4.4	0.1	10.5	$0.202\pm 0.021$

Absolute bin (rad)	$\mathrm {d}\sigma /\mathrm {d}{\Delta \phi ^{e\mu }}$ (fb/rad)	Stat. (%)	$t\bar{t}$ mod. (%)	Lept. (%)	Jet/*b* (%)	Bkg. (%)	$L/E_\mathrm {b}$ (%)	Total (%)	$\mathrm {d}\sigma /\mathrm {d}{\Delta \phi ^{e\mu }}$ (no $\tau$) (fb/rad)
0.00–0.31	$696\pm 30$	2.9	1.4	1.3	0.7	0.9	2.1	4.2	$630\pm 27$
0.31–0.63	$735\pm 29$	2.7	1.3	1.3	0.6	0.9	2.1	4.0	$664\pm 26$
0.63–0.94	$780\pm 31$	2.6	1.3	1.2	0.7	0.9	2.1	3.9	$704\pm 28$
0.94–1.26	$850\pm 33$	2.5	1.2	1.2	0.7	0.9	2.1	3.9	$763\pm 30$
1.26–1.57	$947\pm 36$	2.3	1.1	1.2	0.8	1.0	2.1	3.8	$844\pm 32$
1.57–1.88	$1103\pm 41$	2.2	1.1	1.1	1.1	0.9	2.1	3.7	$977\pm 37$
1.88–2.20	$1235\pm 43$	2.1	1.0	1.0	0.7	0.9	2.1	3.5	$1084\pm 38$
2.20–2.51	$1410\pm 50$	2.0	1.0	1.0	1.1	1.0	2.1	3.5	$1226\pm 44$
2.51–2.83	$1575\pm 56$	2.0	0.9	0.9	1.1	1.1	2.1	3.6	$1353\pm 49$
2.83–3.14	$1696\pm 58$	1.9	0.9	0.9	0.9	1.2	2.1	3.4	$1449\pm 51$

Normalised bin (rad)	$\frac{1}{\sigma }\mathrm {d}\sigma /\mathrm {d}{\Delta \phi ^{e\mu }}$ ($10^{-1}/$rad)	Stat. (%)	$t\bar{t}$ mod. (%)	Lept. (%)	Jet/*b* (%)	Bkg. (%)	$L/E_\mathrm {b}$ (%)	Total (%)	$\frac{1}{\sigma }\mathrm {d}\sigma /\mathrm {d}{\Delta \phi ^{e\mu }}$ (no $\tau$) ($10^{-1}/$rad)
0.00–0.31	$2.010\pm 0.060$	2.8	0.6	0.4	0.5	0.4	0.0	3.0	$2.068\pm 0.062$
0.31–0.63	$2.121\pm 0.057$	2.6	0.5	0.3	0.4	0.4	0.0	2.7	$2.179\pm 0.058$
0.63–0.94	$2.252\pm 0.059$	2.5	0.5	0.3	0.2	0.4	0.0	2.6	$2.311\pm 0.060$
0.94–1.26	$2.454\pm 0.064$	2.4	0.5	0.2	0.6	0.4	0.0	2.6	$2.506\pm 0.065$
1.26–1.57	$2.732\pm 0.064$	2.2	0.4	0.2	0.2	0.4	0.0	2.3	$2.773\pm 0.065$
1.57–1.88	$3.185\pm 0.070$	2.1	0.4	0.1	0.3	0.3	0.0	2.2	$3.207\pm 0.071$
1.88–2.20	$3.566\pm 0.073$	2.0	0.4	0.1	0.2	0.3	0.0	2.0	$3.559\pm 0.072$
2.20–2.51	$4.069\pm 0.079$	1.9	0.4	0.1	0.3	0.3	0.0	1.9	$4.028\pm 0.078$
2.51–2.83	$4.546\pm 0.088$	1.8	0.4	0.2	0.3	0.4	0.0	1.9	$4.443\pm 0.086$
2.83–3.14	$4.897\pm 0.090$	1.7	0.4	0.3	0.1	0.5	0.0	1.8	$4.757\pm 0.088$

**Table 6:** Absolute and normalised differential cross-sections as functions of $p_{\mathrm T}^{e}+p_{\mathrm T}^{\mu }$ (top) and $E^{e}+E^{\mu }$ (bottom). The columns show the bin ranges, measured cross-section and total uncertainty, relative statistical uncertainty, relative systematic uncertainties in various categories (see text), total relative uncertainty, and differential cross-section corrected to remove contributions via $W\rightarrow \tau \rightarrow e/\mu$ decays. Relative uncertainties smaller than 0.05% are indicated by ‘0.0’. The last bin includes overflows where indicated by the ‘+’ sign

Absolute bin (GeV)	$\mathrm {d}\sigma /\mathrm {d}({p_{\mathrm T}^{e}+p_{\mathrm T}^{\mu }})$ (fb/GeV)	Stat. (%)	$t\bar{t}$ mod. (%)	Lept. (%)	Jet/*b* (%)	Bkg. (%)	$L/E_\mathrm {b}$ (%)	Total (%)	$\mathrm {d}\sigma /\mathrm {d}({p_{\mathrm T}^{e}+p_{\mathrm T}^{\mu }})$ (no $\tau$) (fb/GeV)
50–80	$23.02\pm 0.89$	1.7	1.2	1.8	0.7	1.5	2.1	3.8	$18.90\pm 0.73$
80–100	$38.0\pm 1.2$	1.5	1.0	1.2	0.8	0.9	2.1	3.3	$33.0\pm 1.1$
100–120	$34.3\pm 1.1$	1.6	1.0	1.0	0.9	0.9	2.1	3.2	$30.5\pm 1.0$
120–150	$21.00\pm 0.69$	1.7	0.9	1.0	0.8	1.0	2.1	3.3	$18.95\pm 0.63$
150–200	$9.11\pm 0.34$	1.9	1.0	1.1	1.2	1.5	2.2	3.8	$8.27\pm 0.31$
200–250	$3.03\pm 0.16$	3.5	1.3	1.5	1.4	2.4	2.2	5.3	$2.78\pm 0.15$
250–300	$1.08\pm 0.09$	5.7	1.9	2.0	2.9	3.5	2.2	8.1	$0.99\pm 0.08$
300–400+	$0.38\pm 0.04$	7.0	3.3	2.3	3.3	6.5	2.3	11.1	$0.35\pm 0.04$

Normalised bin (GeV)	$\frac{1}{\sigma }\mathrm {d}\sigma /\mathrm {d}({p_{\mathrm T}^{e}+p_{\mathrm T}^{\mu }})$ ($10^{-2}/$GeV)	Stat. (%)	$t\bar{t}$ mod. (%)	Lept. (%)	Jet/*b* (%)	Bkg. (%)	$L/E_\mathrm {b}$ (%)	Total (%)	$\frac{1}{\sigma }\mathrm {d}\sigma /\mathrm {d}({p_{\mathrm T}^{e}+p_{\mathrm T}^{\mu }})$ (no $\tau$) ($10^{-2}/$GeV)
50–80	$0.664\pm 0.015$	1.5	0.4	1.0	0.3	1.2	0.0	2.2	$0.621\pm 0.014$
80–100	$1.097\pm 0.017$	1.3	0.3	0.5	0.4	0.6	0.0	1.6	$1.085\pm 0.018$
100–120	$0.990\pm 0.016$	1.4	0.3	0.3	0.4	0.4	0.0	1.6	$1.003\pm 0.016$
120–150	$0.606\pm 0.010$	1.5	0.3	0.5	0.3	0.4	0.0	1.7	$0.623\pm 0.010$
150–200	$0.2627\pm 0.0062$	1.8	0.4	0.9	0.6	1.0	0.0	2.4	$0.2716\pm 0.0063$
200–250	$0.0875\pm 0.0039$	3.4	0.7	1.4	1.4	2.1	0.1	4.5	$0.0912\pm 0.0041$
250–300	$0.0311\pm 0.0023$	5.6	1.3	2.0	2.5	3.2	0.1	7.3	$0.0326\pm 0.0024$
300–400+	$0.0110\pm 0.0011$	7.0	2.5	2.4	2.9	6.2	0.2	10.4	$0.0116\pm 0.0012$

Absolute bin (GeV)	$\mathrm {d}\sigma /\mathrm {d}({E^{e}+E^{\mu }})$ (fb/GeV)	Stat. (%)	$t\bar{t}$ mod. (%)	Lept. (%)	Jet/*b* (%)	Bkg. (%)	$L/E_\mathrm {b}$ (%)	Total (%)	$\mathrm {d}\sigma /\mathrm {d}({E^{e}+E^{\mu }})$ (no $\tau$) (fb/GeV)
50–80	$4.05\pm 0.22$	3.8	1.3	2.2	1.1	1.2	2.1	5.3	$3.21\pm 0.17$
80–100	$13.68\pm 0.57$	2.5	1.2	1.6	1.2	1.1	2.1	4.2	$11.38\pm 0.48$
100–120	$18.36\pm 0.67$	2.1	1.1	1.3	0.8	0.9	2.1	3.6	$15.67\pm 0.57$
120–150	$19.10\pm 0.64$	1.7	1.1	1.1	0.8	0.9	2.1	3.3	$16.63\pm 0.56$
150–200	$15.79\pm 0.51$	1.5	1.0	0.9	1.0	0.9	2.1	3.2	$13.92\pm 0.45$
200–250	$10.04\pm 0.35$	1.8	1.0	0.9	1.1	1.1	2.1	3.5	$8.97\pm 0.31$
250–300	$6.24\pm 0.25$	2.4	1.0	1.0	1.2	1.4	2.1	4.0	$5.61\pm 0.22$
300–400	$3.04\pm 0.13$	2.5	1.1	1.3	0.9	1.7	2.2	4.2	$2.75\pm 0.12$
400–500	$1.20\pm 0.07$	4.3	1.5	2.0	1.3	2.4	2.2	6.1	$1.10\pm 0.07$
500–700+	$0.48\pm 0.04$	4.8	2.3	2.3	1.6	3.8	2.2	7.4	$0.44\pm 0.03$

Normalised bin (GeV)	$\frac{1}{\sigma }\mathrm {d}\sigma /\mathrm {d}({E^{e}+E^{\mu }})$ ($10^{-3}/$GeV)	Stat. (%)	$t\bar{t}$ mod. (%)	Lept. (%)	Jet/*b* (%)	Bkg. (%)	$L/E_\mathrm {b}$ (%)	Total (%)	$\frac{1}{\sigma }\mathrm {d}\sigma /\mathrm {d}({E^{e}+E^{\mu }})$ (no $\tau$) ($10^{-3}/$GeV)
50–80	$1.172\pm 0.050$	3.8	0.8	1.4	0.7	1.1	0.1	4.3	$1.058\pm 0.046$
80–100	$3.95\pm 0.11$	2.4	0.5	0.8	0.7	0.9	0.0	2.8	$3.75\pm 0.11$
100–120	$5.31\pm 0.12$	2.0	0.4	0.6	0.6	0.7	0.0	2.3	$5.16\pm 0.12$
120–150	$5.521\pm 0.099$	1.6	0.3	0.4	0.4	0.5	0.0	1.8	$5.478\pm 0.099$
150–200	$4.564\pm 0.067$	1.3	0.3	0.3	0.4	0.3	0.0	1.5	$4.585\pm 0.067$
200–250	$2.904\pm 0.055$	1.7	0.4	0.4	0.5	0.4	0.0	1.9	$2.955\pm 0.056$
250–300	$1.803\pm 0.048$	2.3	0.5	0.5	0.9	0.7	0.0	2.6	$1.849\pm 0.049$
300–400	$0.878\pm 0.026$	2.4	0.5	0.8	0.7	1.1	0.0	2.9	$0.907\pm 0.026$
400–500	$0.348\pm 0.018$	4.2	1.0	1.7	1.2	2.0	0.1	5.2	$0.362\pm 0.019$
500–700+	$0.1393\pm 0.0091$	4.7	1.8	1.9	1.2	3.5	0.1	6.5	$0.1463\pm 0.0095$

### Fiducial cross-section measurements

The measured absolute and normalised fiducial differential cross-sections are shown in Table 3 ($p_{\mathrm T}^{\ell }$ and $|\eta ^{\ell }|$), Table 4 ($p_{\mathrm T}^{e\mu }$ and $m^{e\mu }$), Table 5 ($|y^{e\mu }|$ and $\Delta \phi ^{e\mu }$) and Table 6 ($p_{\mathrm T}^{e}+p_{\mathrm T}^{\mu }$ and $E^{e}+E^{\mu }$). Each table shows the measured cross-section values and uncertainties, together with a breakdown of the total uncertainties into components due to data statistics (‘Stat.’), $t\bar{t}$ modelling uncertainties (‘$t\bar{t}$ mod.’), lepton-related uncertainties (‘Lept’), jet and *b*-tagging uncertainties (‘Jet/*b*’), background uncertainties (‘Bkg.’) and luminosity/beam energy uncertainties (‘$L/E_\mathrm {b}$’), corresponding to the breakdown in Sects. 5.1–5.5. The rightmost columns show the cross-sections corrected to remove the contributions where one or both leptons result from $W\rightarrow \tau \rightarrow e/\mu$ decays using Eq. (3). As can also be seen from Fig. 6, the total uncertainties on the normalised differential cross-sections range from 1.2% to around 10%, typically smaller than those for the measurements as a function of the $t\bar{t}$ system kinematics in Ref. [21]. The largest uncertainties are generally statistical (from 1.1% to about 10%), with the background uncertainties also becoming large at high values of some kinematic variables. Other systematic uncertainties due to $t\bar{t}$ modelling, leptons and jets are significantly smaller than the statistical uncertainties, benefiting from cancellations between bins. The cancellations are particularly important when leptons with similar $p_{\text {T}}$ contribute to all bins, as is the case for $\Delta \phi ^{e\mu }$ and the bulk of the $|\eta ^{\ell }|$ and $|y^{e\mu }|$ distributions. The uncertainties in the absolute cross-sections are substantially larger, with the systematic uncertainties due to $t\bar{t}$ modelling and leptons becoming comparable to the statistical uncertainties. The absolute cross-sections also have an uncertainty of 2.1–2.5% from the integrated luminosity measurement, depending on the background level in each bin.

The integrals of the differential cross-sections across all bins of a given distribution ($\sigma ^{t\bar{t}}_{\mathrm {fid}}$ in Eq. (2)) agree in all cases within 0.4% of the integrated fiducial cross-sections of $3.455\pm 0.025$ pb (or $3.043\pm 0.022$ pb excluding $\tau$ contributions) measured within the same fiducial region in Ref. [13, 14]. The quoted uncertainties are statistical.^5^

**Fig. 7:**
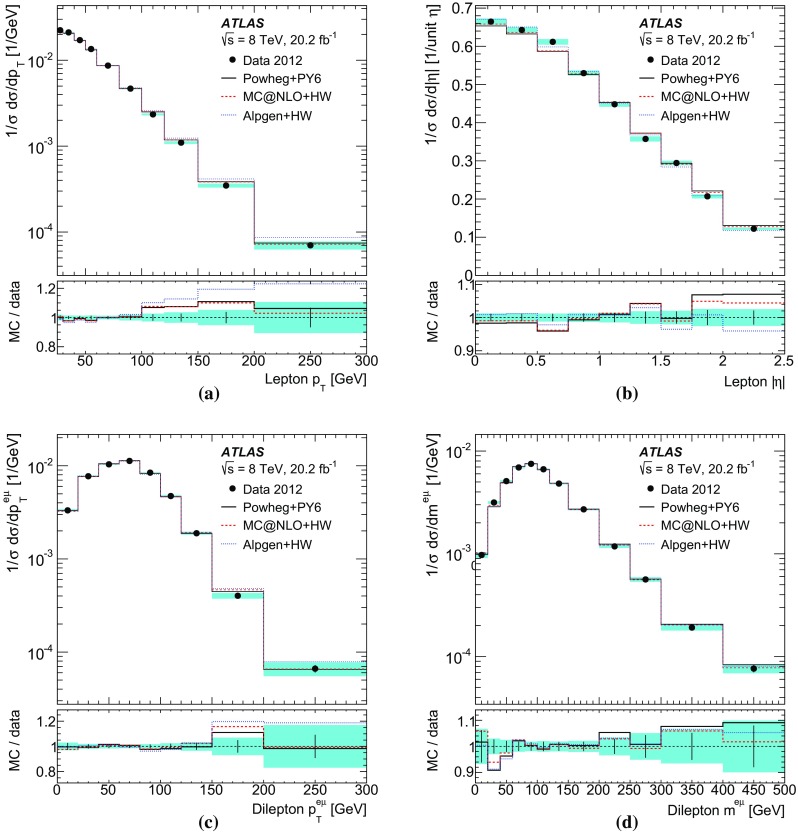
Normalised differential cross-sections as a function of **a** $p_{\mathrm T}^{\ell }$, **b** $|\eta ^{\ell }|$, **c** $p_{\mathrm T}^{e\mu }$ and **d** $m^{e\mu }$. The measured values are shown by the black points with error bars corresponding to the data statistical uncertainties and cyan bands corresponding to the total uncertainties in each bin, and include the contributions via $W\rightarrow \tau \rightarrow e/\mu$ decays. The results are compared to the predictions from the Powheg + Pythia6, MC@NLO + Herwig and Alpgen + Herwig $t\bar{t}$ simulation samples. The lower plots show the ratios of predictions to data, with the error bars indicating the data statistical uncertainties and the cyan bands indicating the total uncertainties in the measurements

**Fig. 8:**
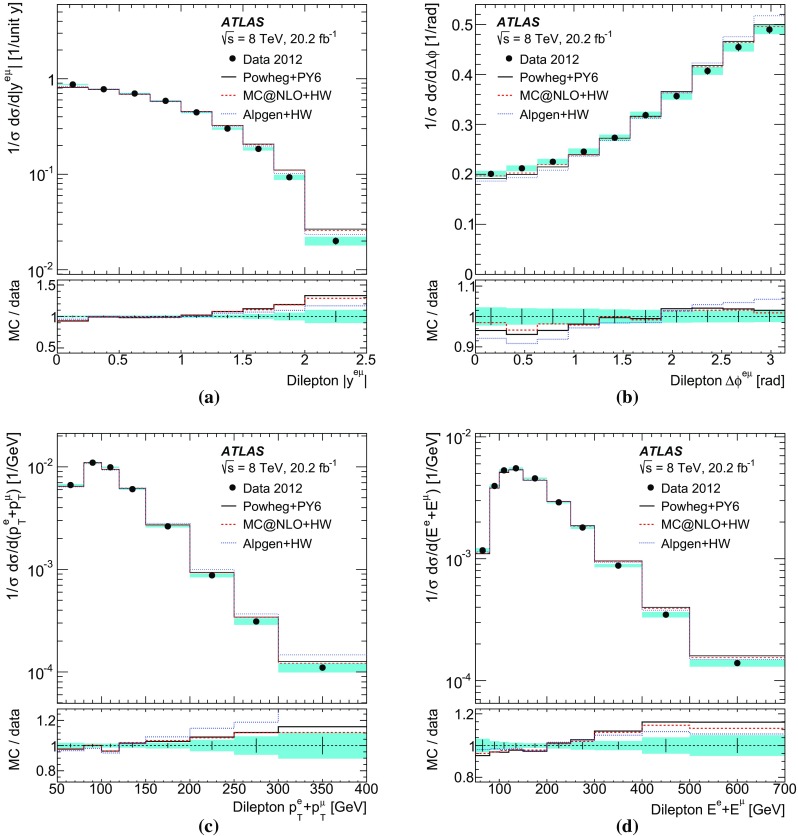
Normalised differential cross-sections as a function of **a** $|y^{e\mu }|$, **b** $\Delta \phi ^{e\mu }$, **c** $p_{\mathrm T}^{e}+p_{\mathrm T}^{\mu }$ and **d** $E^{e}+E^{\mu }$. The measured values are shown by the black points with error bars corresponding to the data statistical uncertainties and cyan bands corresponding to the total uncertainties in each bin, and include the contributions via $W\rightarrow \tau \rightarrow e/\mu$ decays. The results are compared to the predictions from the Powheg + Pythia6, MC@NLO + Herwig and Alpgen + Herwig $t\bar{t}$ simulation samples. The lower plots show the ratios of predictions to data, with the error bars indicating the data statistical uncertainties and the cyan bands indicating the total uncertainties in the measurements

The normalised differential cross-sections are shown graphically in Figs. 7 and 8; in these and later figures, the data points are plotted at the centre of each bin. The measured cross-sections are compared to the particle-level predictions from the Powheg + Pythia6, MC@NLO + Herwig and Alpgen + Herwig $t\bar{t}$ samples within the fiducial volume of the measurement, including the contributions from $W\rightarrow \tau \rightarrow e/\mu$ decays. Similar trends in the description of the measured distributions by the predictions can be seen as for the reconstructed distributions for events with at least one *b*-tagged jet in Figs. 1 and 2.

**Fig. 9:**
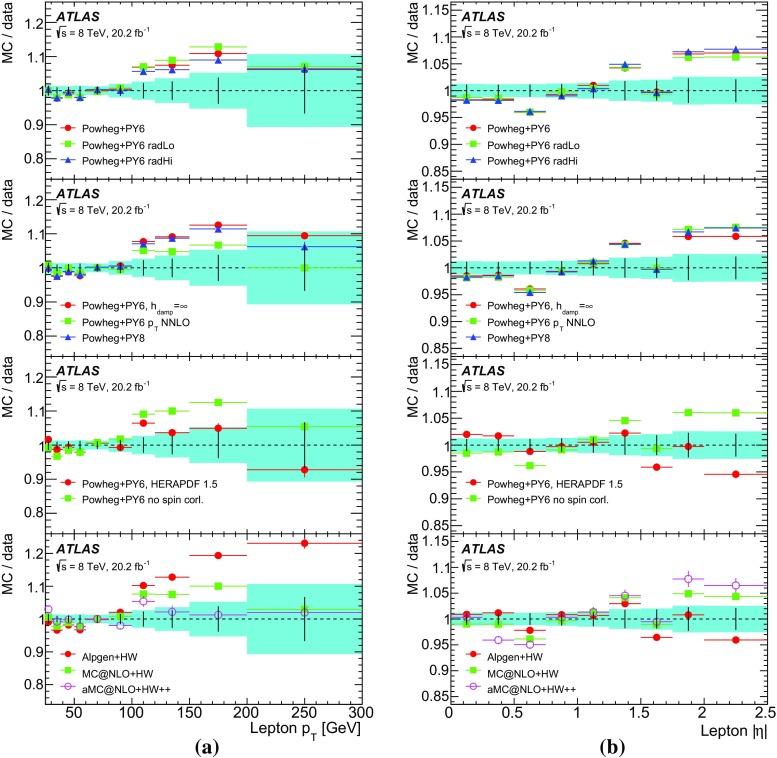
Ratios of predictions of normalised differential cross-sections to data as a function of **a** $p_{\mathrm T}^{\ell }$ and **b** $|\eta ^{\ell }|$. The data statistical uncertainties are shown by the black error bars around a ratio of unity, and the total uncertainties are shown by the cyan bands. The $t\bar{t}$ predictions are shown in four groups from top to bottom, with error bars indicating the uncertainties due to the limited size of the simulated samples

**Fig. 10:**
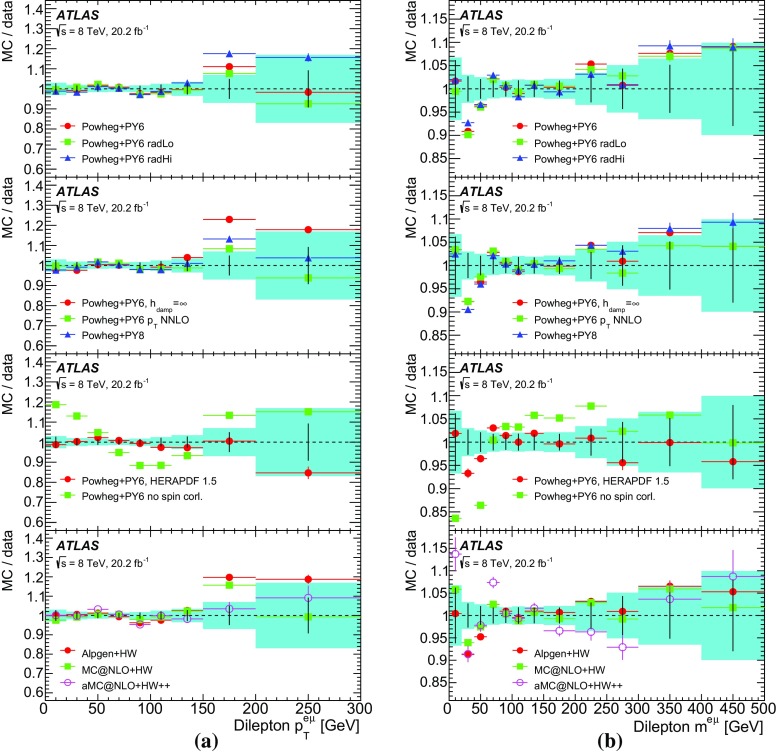
Ratios of predictions of normalised differential cross-sections to data as a function of **a** $p_{\mathrm T}^{e\mu }$ and **b** $m^{e\mu }$. The data statistical uncertainties are shown by the black error bars around a ratio of unity, and the total uncertainties are shown by the cyan bands. The $t\bar{t}$ predictions are shown in four groups from top to bottom, with error bars indicating the uncertainties due to the limited size of the simulated samples

**Fig. 11:**
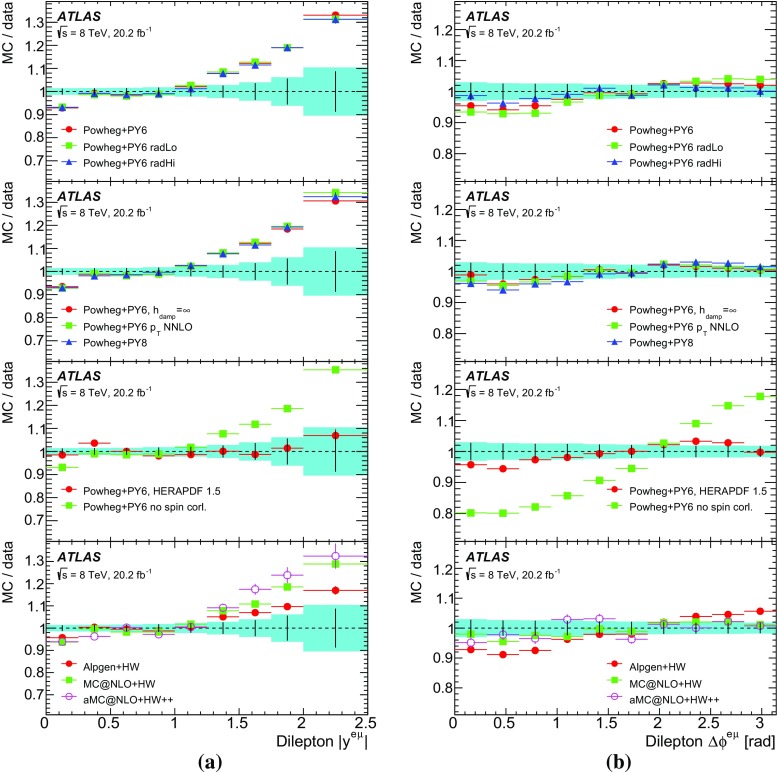
Ratios of predictions of normalised differential cross-sections to data as a function of **a** $|y^{e\mu }|$ and **b** $\Delta \phi ^{e\mu }$. The data statistical uncertainties are shown by the black error bars around a ratio of unity, and the total uncertainties are shown by the cyan bands. The $t\bar{t}$ predictions are shown in four groups from top to bottom, with error bars indicating the uncertainties due to the limited size of the simulated samples

**Fig. 12:**
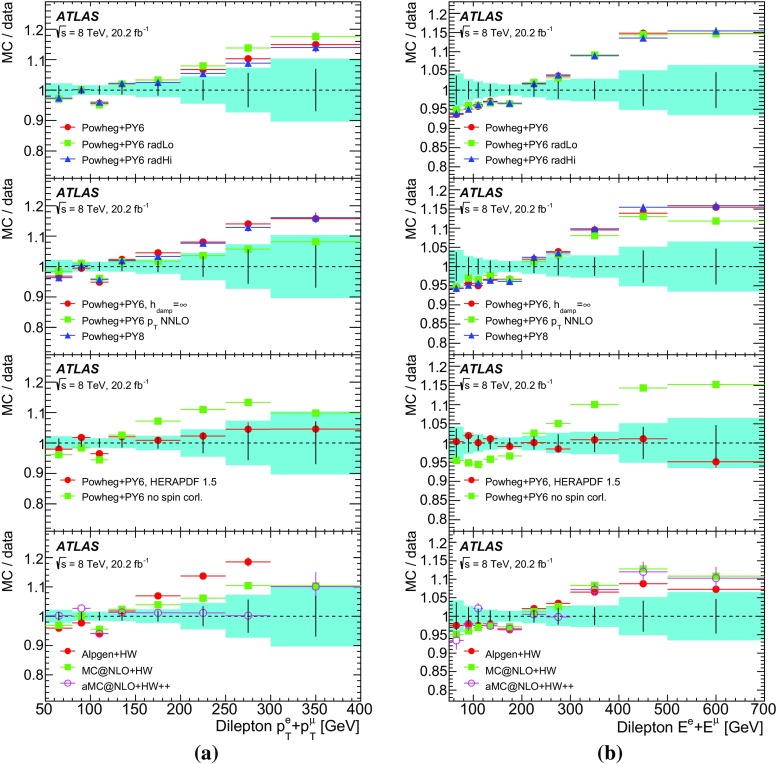
Ratios of predictions of normalised differential cross-sections to data as a function of **a** $p_{\mathrm T}^{e}+p_{\mathrm T}^{\mu }$ and **b** $E^{e}+E^{\mu }$. The data statistical uncertainties are shown by the black error bars around a ratio of unity, and the total uncertainties are shown by the cyan bands. The $t\bar{t}$ predictions are shown in four groups from top to bottom, with error bars indicating the uncertainties due to the limited size of the simulated samples

### Comparison with event generator predictions

The measured normalised differential cross-sections are compared to a larger set of predictions from different $t\bar{t}$ Monte Carlo event generator configurations in Figs. 9, 10, 11 and 12. The figures show the ratios of each prediction to the data as a function of the differential variables, organised into four groups of samples as summarised in Table 7. These event generator setups and tunes were used in ATLAS top physics analyses at $\sqrt{s}=7$ TeV and $\sqrt{s}=8$ TeV, or have been studied in preparation for analyses at $\sqrt{s}=13$ TeV [55, 94, 95].

- **The first group** shows the baseline Powheg + Pythia6 $t\bar{t}$ sample with $h_{\mathrm {damp}}$=$m_t$ (which is also shown in Figs. 7 and 8), together with the two tunes giving more or less parton shower radiation – the Perugia 2012 radHi and radLo tunes [54] coupled to scale and $h_{\mathrm {damp}}$ parameter variations as discussed in Sect. 2.
- **The second group** shows a Powheg + Pythia6 sample with ${h_{\mathrm {damp}}}=\infty$ (i.e. no damping of the first emission), the baseline Powheg + Pythia6 sample with the top quark $p_{\text {T}}$ spectrum reweighted to the NNLO prediction of Ref. [25], and a sample generated with Powheg and ${h_{\mathrm {damp}}}={m_t}$ interfaced to Pythia8 (version 8.186) [46] with the A14 tune [96] and the CTEQ6L1 PDF set for the parton shower, hadronisation and underlying event modelling as described in Ref. [94].
- **The third group** shows a Powheg + Pythia6 sample with ${h_{\mathrm {damp}}}={m_t}$ generated with the HERAPDF 1.5 PDF set [84, 85] instead of CT10,^6^ and a Powheg + Pythia6 sample with ${h_{\mathrm {damp}}}=\infty$ and no simulation of spin correlations between the top and antitop quarks.
- **The fourth group** shows alternative matrix-element event generators – the Alpgen + Herwig and MC@NLO + Herwig samples described in Sect. 2 and shown in Figs. 7 and 8, together with a sample generated using MadGraph5_aMC@NLO 2.2.1 [98] (referred to as aMC@NLO below) and CT10 PDFs, interfaced to Herwig++ [99] with the UE-EE-5 Herwig++ author tune.

The compatibility of each prediction with each measured normalised distribution was assessed quantitatively using a $\chi ^2$ test, calculated as:

5 $$\begin{aligned} \chi ^2 = {\varvec{\Delta }}_{(n-1)}^T {\mathbf S}^{-1}_{(n-1)} {\varvec{\Delta }}_{(n-1)}\,, \end{aligned}$$

where ${\varvec{\Delta }}_{(n-1)}$ is the vector of differences between the measured and predicted normalised differential cross-section in each of the *n* bins, excluding the last one, and ${\mathbf S}_{(n-1)}$ is the corresponding covariance matrix, including both the experimental uncertainties in the measurement and the statistical uncertainties in the predictions. Bin-to-bin correlations in both the statistical (from the normalisation condition) and systematic uncertainties were taken into account via off-diagonal entries. The last bin of each distribution was excluded due to the normalisation condition, rendering the covariance matrix ${\mathbf S}_{(n-1)}$ invertible.^7^ The resulting $\chi ^2$ values, number of degrees of freedom $(n-1)$ and corresponding $\chi ^2$ probability *p*-values are shown for each distribution and prediction in Table 8.

As can be seen from Fig. 9, in the single-lepton $p_{\mathrm T}^{\ell }$ distribution, the data are softer than the predictions from Powheg with CT10 PDFs, interfaced to either Pythia6 or Pythia8. The Powheg-based predictions do not depend strongly on the choice of parton shower/hadronisation model or tune parameters controlling the amount of radiation. However, the agreement with data is improved when using HERAPDF 1.5 or reweighting to the NNLO top quark $p_{\text {T}}$ prediction from Ref. [25]. The predictions from the samples with alternative matrix-element event generators, i.e. MC@NLO + Herwig and Alpgen + Herwig, are also harder than the data, though aMC@NLO + Herwig++ describes the data well. The $p_{\mathrm T}^{e}+p_{\mathrm T}^{\mu }$ and $E^{e}+E^{\mu }$ distributions (Fig. 12) show some similar features to $p_{\mathrm T}^{\ell }$, being softer than the predictions from the Powheg + Pythia6 samples with CT10, and better described with HERAPDF 1.5, and by aMC@NLO + Herwig++.

The predictions for the single lepton $|\eta ^{\ell }|$ and dilepton $|y^{e\mu }|$ distributions (Figs. 9, 10, 11) are insensitive to the choice of parton shower/hadronisation model and tune, and are also insensitive to the top quark $p_{\text {T}}$ reweighting. The data distributions are more central than the predictions of all the NLO event generators (Powheg, MC@NLO and aMC@NLO) with CT10 PDFs, but are better described by Powheg with HERAPDF 1.5, and to a lesser extent also by Alpgen + Herwig, which uses the leading-order CTEQ6L1 PDF. These distributions, whose experimental measurements are limited by statistical uncertainties over the full kinematic range, are thus particularly suitable for constraining PDFs, as explored further in Sect. 7.

The dilepton $p_{\mathrm T}^{e\mu }$ and $m^{e\mu }$ distributions (Fig. 10) are generally well described by all the NLO event generators, except for aMC@NLO which does not model the data well at low $m^{e\mu }$. The $p_{\mathrm T}^{e\mu }$ distribution is sensitive to the amount of parton radiation, and is better described by the radLo than the radHi Powheg + Pythia6 sample, and by ${h_{\mathrm {damp}}}={m_t}$ than ${h_{\mathrm {damp}}}=\infty$. Both distributions are sensitive to the modelling of $t\bar{t}$ spin correlations, and are not well-modelled by the Powheg + Pythia6 sample without spin correlations.

The $\Delta \phi ^{e\mu }$ distribution (Fig. 11) is particularly sensitive to spin correlations, and has been previously used to exclude $t\bar{t}$ simulation models without spin correlation and the pair-production of supersymmetric top squarks with masses close to $m_t$, via template fits to reconstruction-level distributions [100, 101]. The particle-level $\Delta \phi ^{e\mu }$ measurements shown here also exclude the prediction without spin correlations and the LO implementation of spin correlations in the Alpgen + Herwig sample. The $\Delta \phi ^{e\mu }$ distribution is also sensitive to radiation, this time favouring the radHi Powheg + Pythia6 sample.

**Table 7:** Summary of particle-level simulation samples used in the comparison to the corrected data distributions in Sect. 6.2, giving the matrix-element event generator, PDF set, parton shower and associated tune parameter set. The four groups shown correspond to the four panels for each measured distribution shown in Figs. 9, 10, 11 and 12

	Matrix-element	PDF	Parton shower	Tune	Comments
1	Powheg	CT10	Pythia6	P2011C	${h_{\mathrm {damp}}}={m_t}$
	Powheg	CT10	Pythia6	P2012 radHi	${h_{\mathrm {damp}}}=2{m_t}$, $\frac{1}{2}\mu _{F,R}$
	Powheg	CT10	Pythia6	P2012 radLo	${h_{\mathrm {damp}}}={m_t}$, $2\mu _{F,R}$
2	Powheg	CT10	Pythia6	P2011C	${h_{\mathrm {damp}}}=\infty$
	Powheg	CT10	Pythia6	P2011C	${h_{\mathrm {damp}}}={m_t}$, NNLO top $p_{\text {T}}$
	Powheg	CT10	Pythia8	A14	${h_{\mathrm {damp}}}={m_t}$
3	Powheg	HERAPDF 1.5	Pythia6	P2011C	${h_{\mathrm {damp}}}={m_t}$
	Powheg	CT10	Pythia6	P2011C	${h_{\mathrm {damp}}}=\infty$, no spin corl.
4	Alpgen	CTEQ6L1	Herwig+Jimmy	AUET2	incl. $t\bar{t}$ $b\bar{b}$, $t\bar{t}$ $c\bar{c}$
	MC@NLO	CT10	Herwig+Jimmy	AUET2	
	MG5_aMC@NLO	CT10	Herwig++	UE-EE-5	

**Table 8:** The $\chi ^2$ values (top) and associated probabilities (bottom) for comparison of measured normalised differential fiducial cross-sections with various $t\bar{t}$ simulation samples. Probabilities smaller than $10^{-10}$ are shown as zero

Generator	$p_{\mathrm T}^{\ell }$	$|\eta ^{\ell }|$	$p_{\mathrm T}^{e\mu }$	$m^{e\mu }$	$|y^{e\mu }|$	$\Delta \phi ^{e\mu }$	$p_{\mathrm T}^{e}+p_{\mathrm T}^{\mu }$	$E^{e}+E^{\mu }$
$N_{\mathrm dof}$	9	8	8	11	8	9	7	9
Powheg + PY6	13.6	26.3	7.3	14.6	46.6	14.0	11.3	22.7
Powheg + PY6 radLo	15.9	22.9	7.6	14.6	45.6	25.9	14.0	22.0
Powheg + PY6 radHi	10.0	28.2	11.0	12.6	42.0	4.5	9.1	21.4
Powheg + PY6 ${h_{\mathrm {damp}}}=\infty$	17.2	22.5	14.5	12.9	42.8	5.0	15.6	23.4
Powheg + PY6 $p_{\text {T}}$ NNLO	8.3	28.5	6.3	12.1	49.2	7.6	7.6	17.4
Powheg + PY8 ${h_{\mathrm {damp}}}=\infty$	15.1	28.9	8.3	14.4	44.3	13.0	12.7	25.8
Powheg + PY6 HERAPDF 1.5	11.4	11.8	3.6	11.1	6.7	10.3	7.0	1.9
Powheg + PY6 no spin corl.	21.8	23.2	152	100	45.3	279	22.4	27.6
Alpgen + HW	31.2	11.6	15.5	13.7	15.3	36.0	27.4	12.7
MC@NLO + HW	15.7	18.8	9.4	9.3	39.4	7.1	11.8	16.2
aMC@NLO + HW $^{++}$	7.8	29.2	7.6	24.5	46.6	8.2	12.0	13.8
Powheg + PY6	0.14	9 $10^{-4}$	0.51	0.20	2 $10^{-7}$	0.12	0.13	7 $10^{-3}$
Powheg + PY6 radLo	0.070	3 $10^{-3}$	0.48	0.20	3 $10^{-7}$	2 $10^{-3}$	0.052	9 $10^{-3}$
Powheg + PY6 radHi	0.35	4 $10^{-4}$	0.20	0.32	1 $10^{-6}$	0.87	0.24	0.011
Powheg + PY6 ${h_{\mathrm {damp}}}=\infty$	0.045	4 $10^{-3}$	0.069	0.30	1 $10^{-6}$	0.83	0.029	5 $10^{-3}$
Powheg + PY6 $p_{\text {T}}$ NNLO	0.51	4 $10^{-4}$	0.62	0.36	6 $10^{-8}$	0.57	0.36	0.043
Powheg + PY8 ${h_{\mathrm {damp}}}=\infty$	0.089	3 $10^{-4}$	0.41	0.21	5 $10^{-7}$	0.16	0.080	2 $10^{-3}$
Powheg + PY6 HERAPDF 1.5	0.25	0.16	0.89	0.44	0.57	0.32	0.43	0.99
Powheg + PY6 no spin corl.	0.010	3 $10^{-3}$	0	0	3 $10^{-7}$	0	2 $10^{-3}$	1 $10^{-3}$
Alpgen + HW	3 $10^{-4}$	0.17	0.051	0.25	0.054	4 $10^{-5}$	3 $10^{-4}$	0.17
MC@NLO + HW	0.073	0.016	0.31	0.60	4 $10^{-6}$	0.62	0.11	0.063
aMC@NLO + HW $^{++}$	0.56	3 $10^{-4}$	0.47	0.011	2 $10^{-7}$	0.52	0.10	0.13

The $\chi ^2$ formalism of Eq. (5) was extended to consider several normalised distributions simultaneously, by forming vectors $\Delta _i$ where the index runs over the bins of several distributions, excluding the last bin in each one to account for the normalisation condition. The covariance matrix **S** was extended with off-block-diagonal components encoding the correlations between bins of different measured distributions. The statistical correlations between distributions were evaluated using pseudo-experiments generated by sampling from the large simulated $t\bar{t}$ sample discussed in Sect. 4.3. The individual sources of systematic uncertainty were assumed to be fully correlated across the different distributions. Five sets of combined distributions were considered: the combination of $p_{\mathrm T}^{\ell }$ and $p_{\mathrm T}^{e\mu }$, combining all the information from single and dilepton $p_{\text {T}}$; the combination of $p_{\mathrm T}^{e\mu }$, $m^{e\mu }$ and $p_{\mathrm T}^{e}+p_{\mathrm T}^{\mu }$, including all the dilepton kinematic distributions except rapidity; the combination of $|\eta ^{\ell }|$ and $|y^{e\mu }|$, combining the single and dilepton rapidity information; the combination of $|\eta ^{\ell }|$, $|y^{e\mu }|$ and $E^{e}+E^{\mu }$, combining all the distributions with longitudinal information; and the combination of all eight measured distributions, denoted ‘All’. The resulting $\chi ^2$ values, numbers of degrees of freedom and *p*-values are shown for each combination and prediction in Table 9.

The results for the combinations of distributions reflect the observations for the individual distributions. The best modelling of the first two combinations (involving $p_{\mathrm T}^{\ell }$, $p_{\mathrm T}^{e\mu }$, $m^{e\mu }$ and $p_{\mathrm T}^{e}+p_{\mathrm T}^{\mu }$) is given by Powheg + Pythia6 with either HERAPDF 1.5 or with CT10 plus reweighting of the top quark $p_{\text {T}}$ distribution to the NNLO prediction; the radHi variation of Powheg + Pythia6 also does well. The combinations involving $|\eta ^{\ell }|$ and $|y^{e\mu }|$ and the combination of all eight distributions are only well-described by Powheg +Pythia6 with HERAPDF 1.5, and marginally well described by the radHi variation. All other event generator setups (in particular the LO multileg event generator Alpgen) fail to describe some of the distributions, but this could potentially be improved by appropriate parameter tuning and switching to a different PDF set. These results highlight the sensitivity of the differential distributions to the choice of PDF, in particular that of the gluon, as discussed further in Sect. 7. They also indicate that NNLO corrections may be important in describing the kinematics of the decay leptons, as well as for the prediction of the top quark $p_{\text {T}}$ spectrum as discussed in Ref. [25].

**Table 9:** The $\chi ^2$ values (top) and associated probabilities (bottom) for comparison of combinations of measured normalised differential fiducial cross-sections with various $t\bar{t}$ simulation samples. Probabilities smaller than $10^{-10}$ are shown as zero

Generator	$p_{\mathrm T}^{\ell }$, $p_{\mathrm T}^{e}+p_{\mathrm T}^{\mu }$	$p_{\mathrm T}^{e\mu }$, $m^{e\mu }$, $p_{\mathrm T}^{e}+p_{\mathrm T}^{\mu }$	$|\eta ^{\ell }|$, $|y^{e\mu }|$	$|\eta ^{\ell }|$, $|y^{e\mu }|$, $E^{e}+E^{\mu }$	All
$N_{\mathrm dof}$	16	26	16	25	69
Powheg + PY6	20.7	38.2	57.6	70.0	120
Powheg + PY6 radLo	24.6	50.6	57.6	70.6	138
Powheg + PY6 radHi	16.4	29.7	52.3	62.8	98.7
Powheg + PY6 ${h_{\mathrm {damp}}}=\infty$	25.0	40.1	54.2	68.7	113
Powheg + PY6 $p_{\text {T}}$ NNLO	15.1	30.0	60.0	68.2	109
Powheg + PY8 ${h_{\mathrm {damp}}}=\infty$	23.6	37.3	56.8	71.3	121
Powheg + PY6 HERAPDF 1.5	20.1	29.6	22.5	24.5	68.6
Powheg + PY6 no spin corl.	30.2	284	58.3	77.4	462
Alpgen + HW	38.9	79.3	49.3	67.2	154
MC@NLO + HW	23.1	35.2	54.8	65.7	110
aMC@NLO + HW $^{++}$	19.1	45.2	63.1	70.2	128
Powheg + PY6	0.19	0.058	1 $10^{-6}$	4 $10^{-6}$	1 $10^{-4}$
Powheg + PY6 radLo	0.077	3 $10^{-3}$	1 $10^{-6}$	3 $10^{-6}$	2 $10^{-6}$
Powheg + PY6 radHi	0.43	0.28	1 $10^{-5}$	4 $10^{-5}$	0.011
Powheg + PY6 ${h_{\mathrm {damp}}}=\infty$	0.069	0.038	5 $10^{-6}$	6 $10^{-6}$	6 $10^{-4}$
Powheg + PY6 $p_{\text {T}}$ NNLO	0.51	0.27	5 $10^{-7}$	7 $10^{-6}$	2 $10^{-3}$
Powheg + PY8 ${h_{\mathrm {damp}}}=\infty$	0.100	0.071	2 $10^{-6}$	2 $10^{-6}$	1 $10^{-4}$
Powheg + PY6 HERAPDF 1.5	0.21	0.29	0.13	0.49	0.49
Powheg + PY6 no spin corl.	0.017	0	1 $10^{-6}$	3 $10^{-7}$	0
Alpgen + HW	1 $10^{-3}$	3 $10^{-7}$	3 $10^{-5}$	1 $10^{-5}$	2 $10^{-8}$
MC@NLO + HW	0.11	0.11	4 $10^{-6}$	2 $10^{-5}$	1 $10^{-3}$
aMC@NLO + HW $^{++}$	0.26	0.011	2 $10^{-7}$	4 $10^{-6}$	2 $10^{-5}$

### Comparison with fixed-order predictions

The comparisons described in Sect. 6.2 show that the predictions are strongly sensitive to the choice of PDF, and also to the QCD scale (whose variation approximates the effects of missing higher-order corrections) and other parameters related to the amount of radiation. In this section, these aspects are further explored using a set of predictions from the MCFM program (version 6.8) [30], combined with Applgrid (version 1.4.73) [93] to interface to various PDF sets available in LHAPDF (version 6.1.5) [102]. Four recent NLO PDF sets were considered, namely CT14 [103], MMHT14 [104], NNPDF 3.0 [105] and HERAPDF 2.0 [97]. The data were also compared to HERAPDF 1.5 [85] for comparison with the results of Sect. 6.2; the results from these two PDF sets are similar.

MCFM provides an NLO fixed-order prediction of the $t\bar{t}$ process in the dilepton channel, including NLO QCD corrections in both production and decay in the on-shell approximation, and full NLO spin correlations [106]. Only the direct decays of $W\rightarrow e/\mu$ are included, so these predictions were compared to the measurements corrected to remove the leptonic $\tau$ decay contributions. The top quark mass $m_t$ was set to 172.5 GeV. Informed by the discussion in Ref. [107], the central values for the QCD renormalisation and factorisation scales were set to ${m_t}/2$, the lower than typical ($m_t$) scale choice being intended to account for the impact of resummed soft-gluon contributions not included in the fixed-order calculations. The MCFM predictions do not include quantum electrodynamics (QED) final state photon radiation, unlike the experimental measurements where the leptons are dressed with nearby photons as discussed in Sect. 4. Therefore, the MCFM predictions were corrected bin-by-bin using corrections derived from two $t\bar{t}$ samples generated with Pythia8 (version 8.205) [108] and the ATTBAR tune [109] with QED final-state radiation enabled and disabled. These corrections are typically 1–2% on the absolute and always smaller than 1% on the normalised differential cross-sections. No corrections were applied to the normalised $|\eta ^{\ell }|$ and $|y^{e\mu }|$ distributions, as the determined corrections were always smaller than 0.3% and consistent with unity within the simulation statistical uncertainties.

**Fig. 13:**
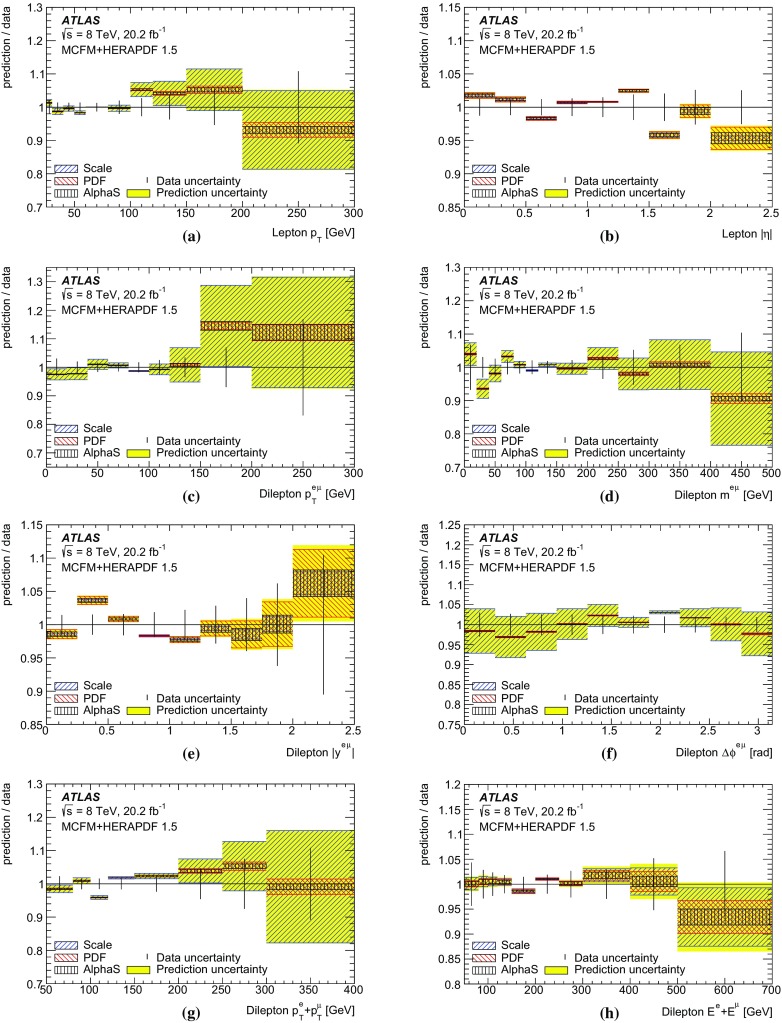
Ratios of MCFM + HERAPDF 1.5 fixed-order predictions of normalised differential cross-sections to data as a function of lepton and dilepton variables. Contributions via $W\rightarrow \tau \rightarrow e/\mu$ decays are not included, and the MCFM predictions have been corrected to include QED final-state radiation effects. The total data uncertainties are shown by the error bars around unity. The separate uncertainties in the predictions from QCD scales, PDFs and the strong coupling constant $\alpha _{\text {S}}$ are shown by the hatched bands, and the total uncertainties in the predictions are shown by the yellow band

The ratios of the MCFM normalised differential cross-section predictions with HERAPDF 1.5 (the PDF set found to best fit the data when comparing with Powheg + Pythia6 samples in Sect. 6.2) to data are shown in Fig. 13. The uncertainties in the predictions include effects from PDFs, QCD scales and the value of the strong coupling constant $\alpha _{\text {S}}$. For each individual component variation, the prediction was renormalised to unity before calculating the shift for each bin; the effects on the normalised cross-section predictions are typically significantly smaller than those on the absolute cross-sections. The PDF uncertainties for CT14 and MMHT were evaluated from the sum in quadrature of the symmetrised up/down variations from each individual eigenvector pair from the PDF error set. For the HERAPDF sets, each pair of eigenvector or model parameter variations was treated as an independent variation. For NNPDF 3.0, the 100 replica sets which represent the NNPDF uncertainty were used to define a full covariance matrix taking into account correlations between the bins of each distribution. The QCD scale uncertainties were evaluated by varying the renormalisation and factorisation scales $\mu _R$ and $\mu _F$ separately, and adding the variations in quadrature. Each scale was varied by factors of one-half and two from its central value (${m_t}/2$), and the resulting variations symmetrised. This procedure was used instead of taking an envelope including simultaneous variations of $\mu _F$ and $\mu _R$ in order to properly account for the correlations between bins of the normalised differential cross-section predictions. Finally, the $\alpha _{\text {S}}$ uncertainty was evaluated using the HERAPDF 1.5 PDF sets with $\alpha _{\text {S}}$ set to 0.116 and 0.120, rescaling the resulting uncertainty to $\Delta \alpha _{\text {S}} =\pm 0.0015$, in line with the corresponding PDF4LHC recommendation [110].

The compatibility of the predictions with the normalised cross-section data was tested quantitatively using the $\chi ^2$ of Eq. (5), updating the covariance matrix **S** to also include the theoretical uncertainties discussed above, including their bin-to-bin correlations via the off-diagonal terms. The resulting $\chi ^2$ and *p*-values are shown as the ‘MCFM + HERAPDF 1.5’ entries in Table 10 for individual distributions, and in Table 11 for the combinations of distributions. As can be seen from these tables and from Fig. 13, MCFM with the HERAPDF 1.5 PDF describes the data well, once all the theoretical uncertainties are taken into account. The predictions for $p_{\mathrm T}^{\ell }$, $p_{\mathrm T}^{e\mu }$, $m^{e\mu }$, $\Delta \phi ^{e\mu }$ and $p_{\mathrm T}^{e}+p_{\mathrm T}^{\mu }$ have large scale uncertainties, which largely cover any differences between the measurements and central predictions with scales $\mu _R=\mu _F={m_t}/2$. The $|\eta ^{\ell }|$ and $|y^{e\mu }|$ distributions have little scale dependence and are more sensitive to PDF variations, but are again well-described within the uncertainties of the HERAPDF 1.5 set. The $\alpha _{\text {S}}$-related uncertainties are small compared to the other two classes.

**Table 10:** The $\chi ^2$ values (top) and associated probabilities (bottom) for comparison of measured normalised differential fiducial cross-sections with the predictions of MCFM with various PDF sets. Contributions via $W\rightarrow \tau \rightarrow e/\mu$ decays are not included, and the MCFM predictions have been corrected to include QED final-state radiation effects. The results take into account the uncertainties in both the measurements and predictions

Generator	$p_{\mathrm T}^{\ell }$	$|\eta ^{\ell }|$	$p_{\mathrm T}^{e\mu }$	$m^{e\mu }$	$|y^{e\mu }|$	$\Delta \phi ^{e\mu }$	$p_{\mathrm T}^{e}+p_{\mathrm T}^{\mu }$	$E^{e}+E^{\mu }$
$N_{\mathrm dof}$	9	8	8	11	8	9	7	9
MCFM + CT14	11.5	14.1	7.2	11.2	13.0	7.2	11.4	11.2
MCFM + MMHT	11.3	12.8	7.2	11.2	12.6	7.1	11.2	9.6
MCFM + NNPDF 3.0	11.7	11.3	7.2	11.4	9.4	7.3	11.5	8.5
MCFM + HERAPDF 1.5	9.1	10.9	6.4	12.1	8.0	6.9	8.5	2.6
MCFM + HERAPDF 2.0	8.4	12.0	6.2	12.4	8.0	6.8	8.0	2.7
MCFM + CT14	0.24	0.080	0.51	0.43	0.11	0.62	0.12	0.27
MCFM + MMHT	0.26	0.12	0.51	0.42	0.13	0.62	0.13	0.38
MCFM + NNPDF 3.0	0.23	0.18	0.52	0.41	0.31	0.61	0.12	0.49
MCFM + HERAPDF 1.5	0.43	0.21	0.61	0.36	0.44	0.65	0.29	0.98
MCFM + HERAPDF 2.0	0.49	0.15	0.63	0.33	0.44	0.66	0.34	0.97

**Table 11:** The $\chi ^2$ values (top) and associated probabilities (bottom) for comparison of combinations of measured normalised differential fiducial cross-sections with the predictions of MCFM with various PDF sets. Contributions via $W\rightarrow \tau \rightarrow e/\mu$ decays are not included, and the MCFM predictions have been corrected to include QED final-state radiation effects. The results take into account the uncertainties in both the measurements and predictions

Generator	$p_{\mathrm T}^{\ell }$, $p_{\mathrm T}^{e}+p_{\mathrm T}^{\mu }$	$p_{\mathrm T}^{e\mu }$, $m^{e\mu }$, $p_{\mathrm T}^{e}+p_{\mathrm T}^{\mu }$	$|\eta ^{\ell }|$, $|y^{e\mu }|$	$|\eta ^{\ell }|$, $|y^{e\mu }|$, $E^{e}+E^{\mu }$	All
$N_{\mathrm dof}$	16	26	16	25	69
MCFM + CT14	19.5	29.6	24.2	32.4	73.0
MCFM + MMHT	19.3	29.6	23.4	30.7	72.0
MCFM + NNPDF 3.0	19.9	29.7	20.1	27.4	69.3
MCFM + HERAPDF 1.5	16.1	28.8	21.5	26.1	68.8
MCFM + HERAPDF 2.0	15.3	30.0	22.7	27.4	69.0
MCFM + CT14	0.24	0.28	0.086	0.15	0.35
MCFM + MMHT	0.25	0.28	0.10	0.20	0.38
MCFM + NNPDF 3.0	0.23	0.28	0.22	0.34	0.47
MCFM + HERAPDF 1.5	0.45	0.32	0.16	0.40	0.48
MCFM + HERAPDF 2.0	0.51	0.27	0.12	0.34	0.48

**Fig. 14:**
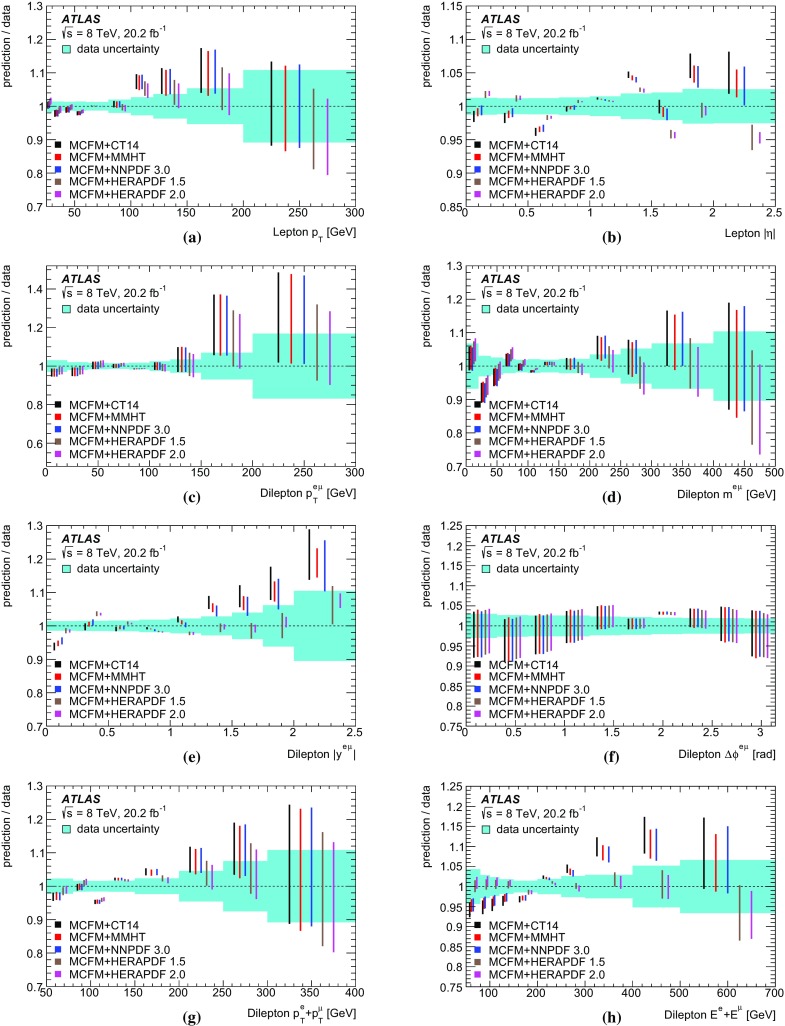
Ratios of MCFM fixed-order predictions of normalised differential cross-sections to data as a function of lepton and dilepton variables, using the CT14, MMHT, NNPDF 3.0, HERAPDF 1.5 and HERAPDF 2.0 PDF sets for the predictions. Contributions via $W\rightarrow \tau \rightarrow e/\mu$ decays are not included, and the MCFM predictions have been corrected to include QED final-state radiation effects. The total data uncertainties are shown by the cyan bands around unity, and the total uncertainty for each prediction (including QCD scales, PDFs, and the strong coupling constant $\alpha _{\text {S}}$) are shown by the vertical bars

The predictions for all five PDF sets (including PDF uncertainties, scaled to 68% CL for CT14, as well as scale and $\alpha _{\text {S}}$ uncertainties) are compared to the data in Fig. 14. The corresponding $\chi ^2$ and *p*-values, including the PDF, scale and $\alpha _{\text {S}}$ uncertanities on the predictions, are shown in Tables 10 and 11. The results for HERAPDF 1.5 and HERAPDF 2.0 are close to the data, whereas the CT14, MMHT and NNPDF 3.0 PDF sets describe the data slightly less well, particularly for $p_{\mathrm T}^{\ell }$, $|\eta ^{\ell }|$, $|y^{e\mu }|$ and $E^{e}+E^{\mu }$. These conclusions are similar to those found for HERAPDF 1.5 and CT10 with the Powheg + Pythia6 setup discussed in Sect. 6.2 above. However, the difference in $\chi ^2$ between the PDF sets is smaller for the fixed-order predictions, as the explicit inclusion of PDF and scale uncertainties in the predictions renders the differences between the central predictions of each PDF less significant. The PDF comparisons would benefit from the availability of predictions including NNLO QCD effects in both the top quark production and decay, which should substantially reduce the scale uncertainties.

## Constraints on the gluon parton distribution function

As a demonstration of the ability of the normalised differential cross-section measurements to constrain the gluon PDF, fits were performed to deep inelastic scattering (DIS) data from HERA I+II [97], with and without the addition of the constraints from $t\bar{t}$ dilepton $|\eta ^{\ell }|$, $|y^{e\mu }|$ and $E^{e}+E^{\mu }$ distributions. As shown in Fig. 13, these distributions are the most sensitive to PDF variations, whilst being less sensitive to QCD scale variations and the value of $m_t$. The fits are based on the predictions from MCFM and ApplGrid discussed in Sect. 6.3, allowing predictions for arbitrary PDF variations to be obtained much faster than if a full NLO plus parton shower event generator setup were to be used. The QCD scales were set to fixed values of $\mu _F=\mu _R={m_t}/2$. The fits were performed using the xFitter package [111, 112], which allows the PDF and other theoretical uncertainties to be included via asymmetric error propagation. In this formalism, the $\chi ^2$ for the compatibility of the measurements with the prediction is expressed by:

6 $$\begin{aligned} \chi ^2 = \sum _{i,j} \left( {\varsigma _i^{\mathrm {exp}}}-{\varsigma _i^{\mathrm {th}}}\right) \ S^{-1}_{\mathrm {exp},ij}({\varsigma _i^{\mathrm {th}}},\varsigma _j^{\mathrm {th}})\ \left( \varsigma _j^{\mathrm {exp}}-\varsigma _j^{\mathrm {th}}\right) ,\nonumber \\ \end{aligned}$$

where $\varsigma _i^{\mathrm {exp}}$ is the measured normalised differential cross-section in bin *i* (equivalent to $\varsigma ^i_{t\bar{t}}$ in Eq. (2)), $\varsigma _i^{\mathrm {th}}$ is the corresponding theoretical prediction, $S_{\mathrm {exp},ij}$ is the covariance matrix of experimental uncertainties including both statistical and systematic contributions, and correlations between bins, and the sums for *i* and *j* run over $n-1$ bins to account for the normalisation condition. Unlike in the formulation of Eq. (5), the covariance matrix is a function of the theoretical predictions, with the statistical uncertainties being rescaled according to the difference between the measured values and the predictions using a Poisson distribution, and the systematic uncertainties being scaled in proportion to the predictions.

Following the formalism outlined in Ref. [113], the covariance matrix was decomposed into a diagonal matrix $\mathbf D$ representing the uncorrelated parts of the uncertainties, and a set of coefficients $\gamma _{ij}^{\mathrm {exp}}$ giving the one standard deviation shift in the measurement *i* for source *j*, where *j* runs over the correlated part of the statistical uncertainties and each source of systematic uncertainty. Each source of experimental uncertainty was then associated with a ‘nuisance parameter’ $b_{j,{\mathrm {exp}}}$ parameterising the associated shift in units of standard deviation. The $\chi ^2$ becomes a function of the set of PDF parameters ${\mathbf p}$ defining the theoretical prediction $\varsigma _i^{\mathrm {th}}$ and the vector of experimental nuisance parameters ${\mathbf b}_{\mathrm {exp}}$, and is given by:

7 $$\begin{aligned} \chi ^2({{\mathbf p}},{\mathbf b}_{\mathrm {exp}})= & {} \sum _i\frac{\left( {\varsigma _i^{\mathrm {exp}}}+\sum _j{\gamma _{ij}^{\mathrm {exp}}}{b_{j,{\mathrm {exp}}}}-{\varsigma _i^{\mathrm {th}}}({{\mathbf p}})\right) ^2}{d_{ii}^2}\nonumber \\&+\sum _j{b_{j,{\mathrm {exp}}}}^2 + L \ , \end{aligned}$$

where $d_{ii}$ are the non-zero elements of the diagonal matrix $\mathbf D$, and the rescaling of the uncertainties leads to the logarithmic term *L*, arising from the likelihood transition to $\chi ^2$ as discussed in Refs. [113, 114]. The $\chi ^2$ was minimised as a function of the PDF parameters ${\mathbf p}$ and the nuisance parameters ${\mathbf b}_{\mathrm {exp}}$, and the value at the minimum provides a compatibility test of the data and prediction.

For the PDF fits, the perturbative order of the DGLAP evolution [115–117] was set to NLO, to match the order of the MCFM predictions. The gluon PDF *g*(*x*) was parameterised as a function of Bjorken-*x* as:

8 $$\begin{aligned} xg(x) = Ax^B(1-x)^C(1+Ex^2)\,e^{Fx}, \end{aligned}$$

which, compared to the standard parameterisation given in Eq. (27) of Ref. [97], removes the negative $A'$ term at low *x* and adds more flexibility at medium and high *x* through the additional terms with the parameters *E* and *F*. The standard parameterisations were used for the quark PDFs, giving a total of 14 free PDF parameters in the vector ${\mathbf p}$, after imposing momentum and valance sum rules, and the constraint that the $\bar{u}$ and $\bar{d}$ contributions are equal at low *x*. Other parameters in the PDF fit were set as described in Ref. [113].

**Fig. 15:**
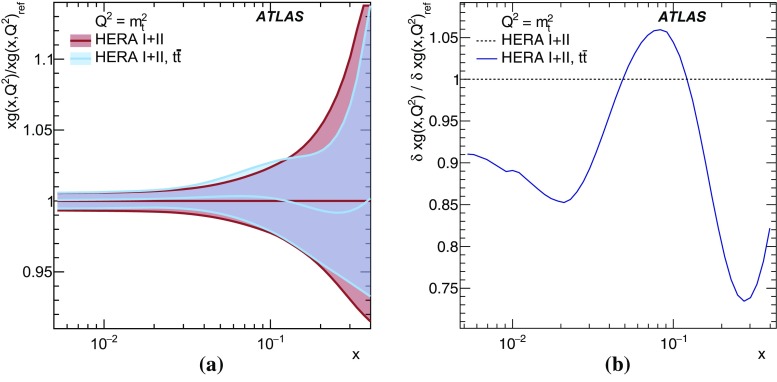
**a** Ratio of the gluon PDF determined from the fit using HERA I+II data plus the normalised differential cross-section distributions as a function of $|\eta ^{\ell }|$, $|y^{e\mu }|$ and $E^{e}+E^{\mu }$ in $t\bar{t}$ events, to the gluon PDF determined from the fit using HERA I+II data alone, as a function of Bjorken-*x*. The uncertainty bands are shown on the two PDFs as the blue and red shading. **b** Ratio of the relative uncertainty in the gluon PDF determined from the fit to HERA I+II plus $t\bar{t}$ data to that from HERA data alone. The PDFs are shown evolved to the scale $Q^2={m_t}^2$ in both cases

The minimised $\chi ^2$ values from the fits without and with the $t\bar{t}$ data are shown in Table 12, which gives the partial $\chi ^2$ for each dataset included in the fit (i.e. the contribution of that dataset to the total $\chi ^2$) and the total $\chi ^2$ for each fit. The partial $\chi ^2$ values indicate that the $t\bar{t}$ data are well-described by the PDF derived from the combined fit, and that the description of the HERA I+II data is not degraded by the inclusion of the $t\bar{t}$ data, i.e. there is no tension between the two datasets. The ratios of the fitted gluon PDF central values with and without the $t\bar{t}$ data included are shown in Fig. 15a, together with the corresponding uncertainties. The ratio of relative uncertainties in the PDFs with and without the $t\bar{t}$ data are shown in Fig. 15b. The inclusion of the $t\bar{t}$ data reduces the uncertainty by typically 10–25% over most of the relevant *x* range.

**Table 12:** Results of the PDF fit to HERA I+II data (left column), and to HERA I+II data plus the normalised differential $t\bar{t}$ cross-sections as a function of $|\eta ^{\ell }|$, $|y^{e\mu }|$ and $E^{e}+E^{\mu }$ (right column). The partial $\chi ^2$ and number of data points for the datasets used in each fit are given, together with the overall $\chi ^2$ and total number of degrees of freedom for each fit

Datasets fitted	HERA I+II	HERA I+II + $t\bar{t}$
Partial $\chi ^2$ / $N_{\mathrm {point}}$		
HERA I+II	1219 / 1056	1219 / 1056
$t\bar{t}$ ($|\eta ^{\ell }|$, $|y^{e\mu }|$, $E^{e}+E^{\mu }$)	–	27 / 25
Total $\chi ^2$ / $N_{\mathrm {dof}}$	1219 / 1042	1247 / 1067

**Fig. 16:**
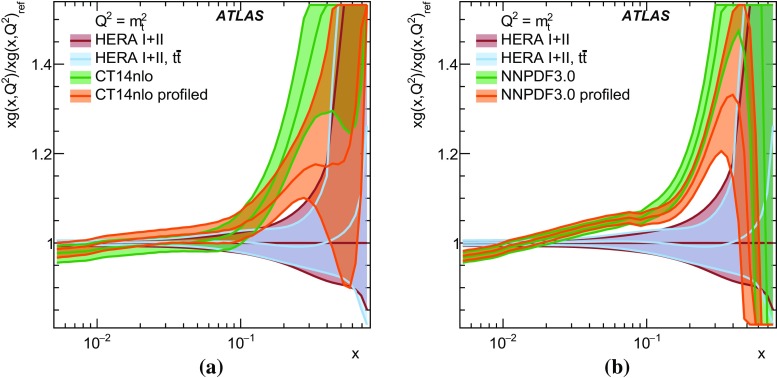
Ratios of various gluon PDFs and their uncertainty bands to the gluon PDF determined from HERA I+II data alone (red shading). The blue shaded band shows the gluon PDF from the fit to HERA I+II data plus the normalised differential cross-section distributions as a function of $|\eta ^{\ell }|$, $|y^{e\mu }|$ and $E^{e}+E^{\mu }$ in $t\bar{t}$ events. The green band shows the gluon PDF from the CT14 [103] PDF set in **a** and the NNPDF 3.0 [105] PDF set in **b**. The orange bands show the result of profiling these PDFs to the $t\bar{t}$ normalised differential cross-section data

The gluon PDF obtained from this procedure is compared to the gluon PDFs from the CT14 [103] and NNPDF 3.0 [105] global PDF sets in Fig. 16. These PDF sets, shown by the green bands, both have a larger high-*x* gluon than preferred by the HERA I+II data, with or without the addition of the $t\bar{t}$ data from this analysis. The impact of the $t\bar{t}$ data on the global PDF sets was investigated using a profiling procedure [113, 118, 119], extending the $\chi ^2$ definition of Eq. (7) to incorporate a vector ${\mathbf b}_{\mathrm {th}}$ of nuisance parameters $b_{k,{\mathrm {th}}}$ expressing the dependence of the theoretical prediction $\varsigma _i^{\mathrm {th}}$ on the uncertainties for a particular PDF set. In this formulation, the $\chi ^2$ definition becomes:

9 $$\begin{aligned} \chi ^2({\mathbf b}_{\mathrm {exp}},{{\mathbf b}_{\mathrm {th}}})= & {} \sum _i\frac{\left( {\varsigma _i^{\mathrm {exp}}}+\sum _j{\gamma _{ij}^{\mathrm {exp}}}{b_{j,{\mathrm {exp}}}}-{\varsigma _i^{\mathrm {th}}}-\sum _k{\gamma _{ik}^{\mathrm {th}}}{b_{k,{\mathrm {th}}}}\right) ^2}{d_{ii}^2}\nonumber \\&+\sum _j{b_{j,{\mathrm {exp}}}}^2+\sum _k{b_{k,{\mathrm {th}}}}^2 + L \ , \end{aligned}$$

where ${b_{k,{\mathrm {th}}}}=\pm 1$ corresponds to the $\pm 1$ standard deviation change of the PDF values according to the *k*th eigenvector of the PDF error set. The values and uncertainties of the nuisance parameters ${b_{k,{\mathrm {th}}}}$ after minimisation of the $\chi ^2$ of Eq. (9) give the profiled PDF with modified central values and uncertainties according to the effect of the $t\bar{t}$ differential cross-section distributions. These profiled PDFs are shown as the orange bands in Fig. 16. Both the CT14 and NNPDF 3.0 gluon PDFs are shifted downwards at high *x* (corresponding to a softer gluon distribution). The effect is larger in the case of CT14, which has larger uncertainties in the gluon PDF in this region.

## Extraction of the top quark mass

The normalised lepton $p_{\mathrm T}^{\ell }$ and dilepton $p_{\mathrm T}^{e\mu }$, $m^{e\mu }$, $p_{\mathrm T}^{e}+p_{\mathrm T}^{\mu }$ and $E^{e}+E^{\mu }$ differential distributions are sensitive to the value of the top quark mass, as already shown in Fig. 5a for $p_{\mathrm T}^{\ell }$ and Fig. 5b for $p_{\mathrm T}^{e\mu }$. Provided that other theoretical uncertainties in the predictions (as discussed in Sect. 6) can be kept under control, fitting these distributions offers a complementary way to measure $m_t$ compared to more traditional determinations from complete reconstruction of the top quark decay products [33–36]. Ref. [32] explores such an approach in detail, arguing that measurements from normalised lepton distributions are less sensitive to the modelling of perturbative and non-perturbative QCD, and are closer to the ideal of a measurement of the top quark pole mass $m_t^{\mathrm {pole}}$ than those employing a direct measurement of the top quark decay products. It also stresses the importance of using several different leptonic observables to probe for inadequacies in the theoretical descriptions of the distributions which may introduce biases in the extracted $m_t$ values. Experimentally, the double-tagging technique employed here results in measurements with little uncertainty from the hadronic components of the $t\bar{t}$ system, again reducing the exposure to QCD modelling compared to the measurements based on reconstructing the top quark decay products.

Several sets of top mass determinations are reported here, based either on predictions from the NLO matrix element event generator Powheg interfaced to Pythia6 and the CT10 PDFs as described in Sect. 2, or on fixed-order predictions with NLO descriptions of the $t\bar{t}$ production and top quark decay from the MCFM program with various PDF sets, as described in Sect. 6.3. In the first case, $m_t$ is extracted either by using a template fit parameterising the predictions as a function of $m_t$ and finding the value which minimises the $\chi ^2$ with respect to the measured data (described in Sect. 8.1), or by calculating moments of the distributions in data and comparing them to the corresponding moments of the predicted distributions for different values of $m_t$ (Sect. 8.2). In the template fit method, the comparisons between data and predictions are performed at particle level, in contrast to the template fits used for the ATLAS $m_t$ measurements based on reconstruction of the top quark decay products [120], where the comparisons are performed at detector level using the reconstructed distributions and fully-simulated Monte Carlo events. The template fit method uses the complete information from the measured distribution, taking into account the uncertainty in each bin, whereas the moments method, advocated in Ref. [32], allows different features of the distribution shapes to be emphasised via the comparisons of moments of different order. The results from these two methods are discussed and compared in Sect. 8.3.

In the mass determination from QCD fixed-order calculations, described in Sect. 8.4, $\chi ^2$ values are calculated for the comparison of data with predictions at different $m_t$ values using the formalism of Eq. (9), and the best-fit $m_t$ is found by polynomial interpolation. This approach is similar to the template fit discussed above; the use of moments was not pursued as it does not exploit the full information of each distribution and does not allow the reduction of uncertainties via constrained nuisance parameters. The $m_t$ value used in the fixed-order predictions corresponds to a well-defined renormalisation scheme, which is the pole mass ($m_t^{\mathrm {pole}}$) scheme within the MCFM implementation. Both the QCD scale uncertainties, representing the effects of missing higher-order corrections beyond NLO, and the PDF uncertainties, are included in the $\chi ^2$ formalism in a natural way. This formalism also allows $m_t$ to be determined using several distributions simultaneously, giving the most precise results from any of the techniques explored here. The results from this method are discussed in Sect. 8.5 and are used to define the final measurement of the top quark mass from the distributions measured in this paper.

**Table 13:** Changes in the top quark mass fitted in data from each lepton or dilepton distribution using the template fit method. The first row shows the shifts when changing the Powheg parameter $h_{\mathrm {damp}}$ from $\infty$ to $m_t$, a correction which is applied to the results quoted in Table 14. The second row shows additional shifts when reweighting the top quark $p_{\text {T}}$ in Powheg + Pythia6 to the NNLO prediction of Ref. [25]

Mass shift (GeV)	$p_{\mathrm T}^{\ell }$	$p_{\mathrm T}^{e\mu }$	$m^{e\mu }$	$p_{\mathrm T}^{e}+p_{\mathrm T}^{\mu }$	$E^{e}+E^{\mu }$
Powheg (${h_{\mathrm {damp}}}=\infty )\rightarrow ({h_{\mathrm {damp}}}={m_t})$	0.9	3.0	−1.3	0.9	0.5
Top $p_{\text {T}}$ NNLO reweighting	1.8	0.3	2.2	1.3	1.3

### Mass extraction using template fits

In the template fit method, the best fit top quark mass for each measured distribution was obtained by minimising the $\chi ^2$ for the comparison of that distribution with predictions at different values of $m_t$, defined analogously with Eq. (5):

10 $$\begin{aligned} \chi ^2({m_t}) = {\varvec{\Delta }}_{(n-1)}^T({m_t})\ {\mathbf S}^{-1}_{(n-1)}\ {\varvec{\Delta }}_{(n-1)}({m_t})\ , \end{aligned}$$

where ${\varvec{\Delta }}_{(n-1)}({m_t})$ represents the vector of differences between the measured normalised differential cross-section value and the prediction for a particular value of $m_t$. The latter were obtained from a set of seven particle-level $t\bar{t}$ samples generated using Powheg + Pythia6 with ${h_{\mathrm {damp}}}=\infty$ and the CT10 PDF set, for values of $m_t$ ranging from 165–180 GeV in 2.5 GeV steps. The variation of the cross-section in each bin was parameterised with a second-order polynomial in $m_t$, allowing predictions for arbitrary values in the considered range to be obtained by interpolation. An additional multiplicative correction was applied to the predictions in each bin, based on the ratio of predictions from Powheg + Pythia6 samples with ${h_{\mathrm {damp}}}={m_t}$ and ${h_{\mathrm {damp}}}=\infty$, in order to correspond to the baseline event generator choice with ${h_{\mathrm {damp}}}={m_t}$. As shown in Table 13, the effects of this correction range from $-1.3$ to 3.0 GeV depending on the distribution fitted, and were assumed to be independent of $m_t$. As the predictions include the simulation of leptons from $W\rightarrow \tau \rightarrow e/\mu$ decays, the comparisons are made with the experimental results including leptons from $\tau$ decays, as in Sect. 6.2.

The template fit method was tested with pseudo-experiments based on fully-simulated $t\bar{t}$ samples with $m_t$ values in the range 165–180 GeV plus non-$t\bar{t}$ backgrounds. The pseudo-data were processed through the complete analysis procedure starting from the observed event counts in each bin, using the methodology described in Sect. 4.3. The baseline Powheg + Pythia6 $t\bar{t}$ sample with ${m_t}=172.5$ GeV was used as reference for the calculation of $G^i_{e\mu }$, $C^i_b$, $N_1^{i,\mathrm {bkg}}$ and $N_2^{i,\mathrm {bkg}}$. No statistically significant biases were found for the fits based on the $p_{\mathrm T}^{\ell }$, $p_{\mathrm T}^{e\mu }$ and $m^{e\mu }$ distributions, but biases of up to 0.6 GeV for $p_{\mathrm T}^{e}+p_{\mathrm T}^{\mu }$ and 0.9 GeV for $E^{e}+E^{\mu }$ were found in pseudo-experiments with true $m_t$ values 5 GeV away from the 172.5 GeV reference, still small compared to the expected statistical uncertainties using these distributions. These biases were corrected in the fit results from data discussed in Sect. 8.3 below. The pseudo-experiments were also used to check the statistical uncertainties returned by the fit via the pull distributions, which were generally found to be within ± 5% of unity.

Both the data statistical uncertainty and experimental systematic uncertainties on the measurements of the differential distributions are included in the matrix ${\mathbf S}_{(n-1)}$ in Eq. (10). Further uncertainties in the extracted $m_t$ value arise from the choices of PDFs and event generator setup for the predictions. The PDF uncertainties were assessed from the variations in normalised $t\bar{t}$ differential cross-section distributions predicted by MC@NLO + Herwig reweighted using the error sets of the CT10, MSTW and NNPDF 2.3 PDF sets as described in Sect. 5.1. The event generator setup uncertainties were assessed as the quadrature sum of a $t\bar{t}$ generator uncertainty and a QCD radiation uncertainty. The former was obtained from the comparison of results using Powheg + Pythia6 (${h_{\mathrm {damp}}}={m_t}$) and MC@NLO + Herwig samples (thus varying both the matrix element and parton shower generator). The latter was defined as half the variation from fits using the Powheg + Pythia6 samples with radLo and radHi tunes discussed in Sect. 2. In all cases, the uncertainties were defined from the difference in $m_t$ values obtained when fitting the two samples as pseudo-data, using the full experimental covariance matrix from the data measurement and the standard templates obtained from the Powheg + Pythia6 samples as discussed above.

### Mass extraction using moments

Top quark mass information can also be derived from a measured distribution by calculating Mellin moments of the distribution, and comparing the values observed to a calibration curve obtained from predictions with different values of $m_t$ [32]. The *k*th order Mellin moment $\mu ^{(k)}$ for a distribution $D(x)\equiv \mathrm {d}\sigma /\mathrm {d}x$ as a function of a kinematic variable *x* is defined as:

11 $$\begin{aligned} {\mu ^{(k)}}=\frac{1}{\sigma _{\mathrm {fid}}} \int x^k D(x)\,\mathrm {d}x \,, \end{aligned}$$

where the integral is taken over the fiducial region, and the total fiducial cross-section $\sigma _{\mathrm {fid}}=\int D(x)\,\mathrm {d}x$. These moments can in principle be evaluated without binning the data, since for leptonic observables, the value *x* for each individual event is measured with high precision. However, for the purpose of this analysis, these moments were approximated by binned moments $\Theta ^{(k)}$ evaluated as:

12 $$\begin{aligned} {\Theta ^{(k)}}=\sum _i {\varsigma ^i_{t\bar{t}}}X_i\,, \ X_i=<x^k> \mathrm {in\ bin}\ i\,, \end{aligned}$$

where $\varsigma ^i_{t\bar{t}}$ is the fraction of the total fiducial $t\bar{t}$ cross-section in bin *i* (Eq. (2)) and $X_i$ is the mean value of *x* for all the events falling in bin *i*. The values of $X_i$, which act as weights for each bin *i* of each kinematic distribution when calculating the moment *k*, were evaluated using the baseline Powheg + Pythia6 sample and kept constant when evaluating moments for the data and all simulation samples. Calibration curves for the first, second and third moments $\Theta ^{(1)}$, $\Theta ^{(2)}$ and $\Theta ^{(3)}$ were derived using the same set of Powheg + Pythia6 samples with top quark masses in the range 165–180 GeV as used for the template analysis. The dependencies of ${\Theta ^{(k)}}$ on $m_t$ were found to be well-described by second-order polynomials ${\Theta ^{(k)}}({m_t})=P_2({m_t})$. A constant offset in each moment was used to correct to the calibration appropriate for ${h_{\mathrm {damp}}}={m_t}$ samples, and the polynomial inverted to obtain the $m_t$ value corresponding to a given measured *k*th moment $\Theta ^{(k)}$.

The extraction procedure was tested for bias with pseudo-experiments in the same way as for the template fit. The observed biases were of similar size to those in the template fit, and were corrected in the same way. Experimental systematic uncertainties were evaluated by calculating the moments from the normalised cross-section distribution with each bin shifted by one standard deviation of each systematic, and translating the resulting shift in $\Theta ^{(k)}$ to a shift in $m_t$. Uncertainties in the predictions due to the choice of PDFs, $t\bar{t}$ generator and radiation settings were assessed in the same way, i.e. from the shifts in $\Theta ^{(k)}$ predicted by each of the alternative samples.

### Results from the template and moment methods

**Fig. 17:**
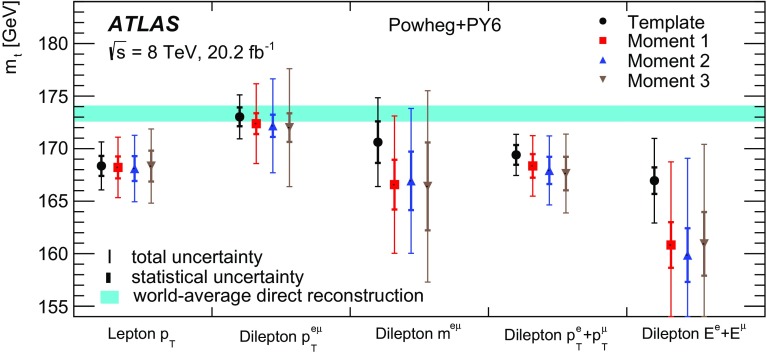
Measurements of the top quark mass using templates derived from Powheg + Pythia6 with the CT10 PDF set. The results from fitting templates of the single lepton $p_{\mathrm T}^{\ell }$ and dilepton $p_{\mathrm T}^{e\mu }$, $m^{e\mu }$, $p_{\mathrm T}^{e}+p_{\mathrm T}^{\mu }$ and $E^{e}+E^{\mu }$ distributions, and from the first, second and third moments of these distributions, are shown. For comparison, the world-average of mass measurements from reconstruction of the top quark decay products and its uncertainty [121] is shown by the cyan band

The results of applying the template and first, second and third moment methods to each of the $p_{\mathrm T}^{\ell }$, $p_{\mathrm T}^{e\mu }$, $m^{e\mu }$, $p_{\mathrm T}^{e}+p_{\mathrm T}^{\mu }$ and $E^{e}+E^{\mu }$ distributions using predictions from Powheg + Pythia6 and CT10 PDFs are shown in Table 14 and Fig. 17. The table shows the $\chi ^2$ at the best fit mass for each distribution, and the breakdown of uncertainties into statistical, experimental systematic and theoretical contributions, evaluated as discussed in Sect. 8.1. For the template fits, the data statistical uncertainty was evaluated from a $\chi ^2$ minimisation of Eq. (10) with only statistical uncertainties included in the covariance matrix $\mathbf S$. The experimental systematic uncertainty was evaluated as the quadrature difference between the total uncertainty (when including both statistical and experimental systematic uncertainties in $\mathbf S$), and the data statistical uncertainty. For the moments method, the statistical and experimental systematic uncertainties were evaluated directly on the moments $\Theta ^{(k)}$ as discussed in Sect. 8.2.

**Table 14:** Measurements of the top quark mass from individual template fits to the lepton $p_{\mathrm T}^{\ell }$ and dilepton $p_{\mathrm T}^{e\mu }$, $m^{e\mu }$, $p_{\mathrm T}^{e}+p_{\mathrm T}^{\mu }$ and $E^{e}+E^{\mu }$ distributions, and using the first, second and third moments of these distributions. The data are compared to predictions from Powheg + Pythia6 with the CT10 PDF set. The $\chi ^2$ value at the best-fit mass for each distribution (for the template fits only), the fitted mass with its total uncertainty, and the individual uncertainty contributions from data statistics, experimental systematics, and uncertainties in the predictions due to the choice of $t\bar{t}$ event generator and the modelling of QCD radiation are shown

Template	$p_{\mathrm T}^{\ell }$	$p_{\mathrm T}^{e\mu }$	$m^{e\mu }$	$p_{\mathrm T}^{e}+p_{\mathrm T}^{\mu }$	$E^{e}+E^{\mu }$
$\chi ^2/N_{\mathrm dof}$	8.1/8	7.5/7	13.9/10	8.0/6	12.5/8
$m_t$ (GeV)	$168.4\pm 2.3$	$173.0\pm 2.1$	$170.6\pm 4.2$	$169.4\pm 2.0$	$166.9\pm 4.0$
Data statistics	$\pm \,1.0$	$\pm \,0.9$	$\pm \,2.0$	$\pm \,0.9$	$\pm \,1.3$
Expt. systematic	$\pm \,1.6$	$\pm \,1.0$	$\pm \,3.1$	$\pm \,1.6$	$\pm \,1.5$
PDF uncertainty	$\pm \,1.0$	$\pm \,0.2$	$\pm \,1.6$	$\pm \,0.6$	$\pm \,3.4$
$t\bar{t}$ generator	$\pm \,0.4$	$\pm \,1.4$	$\pm \,1.4$	$\pm \,0.4$	$\pm \,1.1$
QCD radiation	$\pm \,0.7$	$\pm \,0.8$	$\pm \,0.5$	$\pm \,0.2$	$\pm \,0.2$

Moment 1	$p_{\mathrm T}^{\ell }$	$p_{\mathrm T}^{e\mu }$	$m^{e\mu }$	$p_{\mathrm T}^{e}+p_{\mathrm T}^{\mu }$	$E^{e}+E^{\mu }$
$m_t$ (GeV)	$168.2\pm 2.9$	$172.4\pm 3.8$	$166.6\pm 6.5$	$168.4\pm 2.9$	$160.8\pm 7.9$
Data statistics	$\pm \,1.0$	$\pm \,1.0$	$\pm \,2.4$	$\pm \,1.1$	$\pm \,2.2$
Expt. systematic	$\pm \,2.1$	$\pm \,1.6$	$\pm \,3.8$	$\pm \,2.1$	$\pm \,3.1$
PDF uncertainty	$\pm \,1.2$	$\pm \,0.3$	$\pm \,2.9$	$\pm \,1.1$	$\pm \,6.7$
$t\bar{t}$ generator	$\pm \,0.2$	$\pm \,1.3$	$\pm \,3.4$	$\pm \,0.2$	$\pm \,2.0$
QCD radiation	$\pm \,1.2$	$\pm \,3.0$	$\pm \,1.4$	$\pm \,1.1$	$\pm \,0.2$

Moment 2	$p_{\mathrm T}^{\ell }$	$p_{\mathrm T}^{e\mu }$	$m^{e\mu }$	$p_{\mathrm T}^{e}+p_{\mathrm T}^{\mu }$	$E^{e}+E^{\mu }$
$m_t$ (GeV)	$168.1\pm 3.2$	$172.2\pm 4.5$	$166.9\pm 6.9$	$167.9\pm 3.3$	$159.9\pm 9.2$
Data statistics	$\pm \,1.2$	$\pm \,1.1$	$\pm \,2.8$	$\pm \,1.3$	$\pm \,2.6$
Expt. systematic	$\pm \,2.3$	$\pm \,2.0$	$\pm \,4.3$	$\pm \,2.4$	$\pm \,3.4$
PDF uncertainty	$\pm \,1.3$	$\pm \,0.4$	$\pm \,3.3$	$\pm \,1.3$	$\pm \,7.8$
$t\bar{t}$ generator	$\pm \,0.4$	$\pm \,1.2$	$\pm \,3.2$	$\pm \,0.4$	$\pm \,2.4$
QCD radiation	$\pm \,1.2$	$\pm \,3.7$	$\pm \,0.7$	$\pm \,1.3$	$\pm \,0.2$

Moment 3	$p_{\mathrm T}^{\ell }$	$p_{\mathrm T}^{e\mu }$	$m^{e\mu }$	$p_{\mathrm T}^{e}+p_{\mathrm T}^{\mu }$	$E^{e}+E^{\mu }$
$m_t$ (GeV)	$168.3\pm 3.5$	$172.0\pm 5.6$	$166.4\pm 9.1$	$167.6\pm 3.8$	$160.9\pm 9.5$
Data statistics	$\pm \,1.5$	$\pm \,1.4$	$\pm \,4.2$	$\pm \,1.6$	$\pm \,3.0$
Expt. systematic	$\pm \,2.5$	$\pm \,2.6$	$\pm \,6.0$	$\pm \,2.7$	$\pm \,3.7$
PDF uncertainty	$\pm \,1.5$	$\pm \,0.6$	$\pm \,4.1$	$\pm \,1.4$	$\pm \,7.8$
$t\bar{t}$ generator	$\pm \,0.6$	$\pm \,1.1$	$\pm \,3.5$	$\pm \,0.7$	$\pm \,2.4$
QCD radiation	$\pm \,1.1$	$\pm \,4.6$	$\pm \,0.2$	$\pm \,1.4$	$\pm \,0.2$

**Fig. 18:**
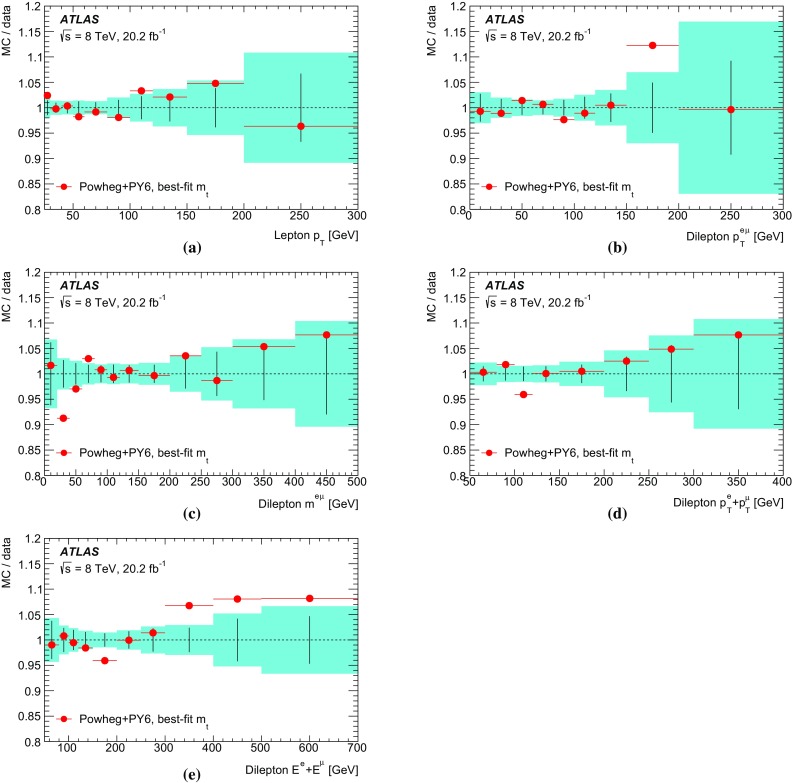
Ratios of predictions of normalised differential cross-sections to data as a function of **a** $p_{\mathrm T}^{\ell }$, **b** $p_{\mathrm T}^{e\mu }$, **c** $m^{e\mu }$, **d** $p_{\mathrm T}^{e}+p_{\mathrm T}^{\mu }$ and **e** $E^{e}+E^{\mu }$, with the prediction taken from Powheg + Pythia6 with the CT10 PDF at the best-fit top quark mass $m_t$ for each distribution. The data statistical uncertainties are shown by the black error bars around a ratio of unity, and the total experimental uncertainties by the cyan band

The ratios of predictions to data at the best-fit top quark mass found by the application of the template fit method to each distribution are shown in Fig. 18. The data are generally well-described by these predictions, as can also be seen from the $\chi ^2$ values in Table 14, except for the $E^{e}+E^{\mu }$ distribution. This distribution is quite sensitive to PDFs as well as $m_t$, and is better described by the HERAPDF PDFs than the CT10 PDFs used here to extract $m_t$, resulting in a low fitted value with a large PDF uncertainty, and a large variation between the template and moment fit results. Total uncertainties in $m_t$ of about 2 GeV are obtained from the template fits to the $p_{\mathrm T}^{\ell }$, $p_{\mathrm T}^{e\mu }$ and $p_{\mathrm T}^{e}+p_{\mathrm T}^{\mu }$ distributions. These results have relatively small theoretical uncertainties, and the experimental uncertainties are dominated by $t\bar{t}-Wt$ interference and the electron energy scale. The $m^{e\mu }$ distribution is intrinsically less sensitive to $m_t$, having larger statistical, experimental and theoretical systematic uncertainties. The results from the extraction based on moments have larger uncertainties than those from the template fit, reflecting that the moments do not take into account the relative precision on the different bins of the distributions, and that the higher moments are more sensitive to the tails of the distributions, which are less precisely measured and subject to larger theoretical uncertainties. Within each distribution, the $m_t$ values from the different moments are close, though 3–4 GeV lower than the template fit results for $m^{e\mu }$, and up to 7 GeV lower in the case of $E^{e}+E^{\mu }$.

The central values of the template fit results from the five distributions exhibit a spread (envelope) of about 6 GeV. The results from the fits of $p_{\mathrm T}^{\ell }$ and $p_{\mathrm T}^{e}+p_{\mathrm T}^{\mu }$ lie 4–5 GeV below that from $p_{\mathrm T}^{e\mu }$, which is close to the world-average mass value from reconstruction of top quark decay products of $173.34\pm 0.76$ GeV [121]. The consistency of the fit results was assessed by combining them using the best linear unbiased estimate (BLUE) technique [122]. Correlations in the statistical uncertainties were assessed using pseudo-experiments as described in Sect. 4.3. Correlations between systematic uncertainties were determined by assuming the effects on $m_t$ from each individual experimental or theoretical component to be fully correlated between distributions. PDF uncertainties were assessed using the eigenvector pairs of the CT10 PDF only. The combination of all five distributions has a $\chi ^2$ probability of 4%, indicating that the systematic uncertainties may be underestimated.

The Powheg + Pythia6 $t\bar{t}$ samples used here do not provide a good modelling of the top quark $p_{\text {T}}$ spectrum [18–20, 22, 23], potentially biasing the results. The size of this possible bias was explored by fitting the distributions from the Powheg + Pythia6 baseline sample reweighted to the top quark $p_{\text {T}}$ spectrum calculated at NNLO precision in Ref. [25]. The reweighted sample gives a better description of the $p_{\mathrm T}^{\ell }$ and $p_{\mathrm T}^{e}+p_{\mathrm T}^{\mu }$ distributions, as can be seen from the $\chi ^2$ values for ‘Powheg + PY6 $p_{\text {T}}$ NNLO’ in Table 8. The mass shifts between the baseline and reweighted samples, representing the amount that the top quark mass measured in data would be shifted upwards if the templates were based on reweighted samples, are shown in Table 13. These shifts are larger (1.3–1.8 GeV) for $p_{\mathrm T}^{e}+p_{\mathrm T}^{\mu }$ and $p_{\mathrm T}^{\ell }$ than for $p_{\mathrm T}^{e\mu }$ (0.3 GeV), and would bring the results shown in Fig. 17 into closer agreement with each other. However, given that this reweighting is relatively crude, and does not take into account the potential NNLO effects on other distributions important for modelling the lepton and dilepton kinematics (e.g. the invariant mass and rapidity of the $t\bar{t}$ system), the shifts are taken to be purely indicative, and no attempt has been made to correct the quoted central values for these effects. The predictions for the $p_{\mathrm T}^{\ell }$ and $p_{\mathrm T}^{e}+p_{\mathrm T}^{\mu }$ distributions are also sensitive to the choice of PDF. The PDF uncertainties shown for $p_{\mathrm T}^{\ell }$ and $p_{\mathrm T}^{e}+p_{\mathrm T}^{\mu }$ in Table 14 are significantly larger than those for $p_{\mathrm T}^{e\mu }$, and as shown in Sect. 6.2, the Powheg + Pythia6 sample generated using HERAPDF 1.5 instead of CT10 gives a significantly better description of both distributions at ${m_t}=172.5$ GeV.

The predictions from Powheg + Pythia6, based on NLO matrix elements interfaced to parton showers, hence suffer from significant uncertainties due to missing NNLO corrections and lack of knowledge of the PDFs. Consequently, they do not have sufficient precision to extract the top quark mass from individual distributions with a theoretical uncertainty better than about 2 GeV, slightly larger than the uncertainties corresponding to the precision of the experimental measurements. These limitations are addressed by the approach discussed below, where several distributions are fitted simultaneously to extract $m_t$ whilst constraining the uncertainties in the theoretical predictions.

### Mass extraction using fixed-order predictions

The NLO fixed-order predictions for each distribution were generated using MCFM as discussed in Sect. 6.3, for top quark masses in the range 161–180 GeV in steps of 0.5 GeV, with various PDF choices. The $\chi ^2$ for the consistency of each prediction with the data was calculated using Eq. (9), incorporating both PDF and QCD scale uncertainties into the theoretical uncertainties represented by the nuisance parameters ${\mathbf b}_{\mathrm {th}}$. The central scales were again chosen to be $\mu _F=\mu _R={m_t}/2$, with the values varying with $m_t$ in the mass scan, and independent variations of $\mu _F$ and $\mu _R$ by factors of two and one-half defining the one standard deviation up and down scale variations. The $\chi ^2$ was evaluated at each mass point, and interpolated using a fourth-order polynomial. The asymmetric uncertainty in the fitted value of $m_t$ was defined as the points at which the $\chi ^2$ increases by one unit either side of the minimum point. This uncertainty naturally includes both experimental statistical and systematic uncertainties in the measurements, and theoretical uncertainties due to PDFs and QCD scale choices.

In this method, the top quark mass can be extracted from each measured distribution individually, or from the combination of several distributions, where the sum *i* in Eq. (9) runs over the bins of all considered distributions, and the experimental covariance matrix includes both statistical and systematic correlations between bins of the same and different distributions, evaluated as discussed in Sect. 6.2. When fitting several distributions simultaneously, the system is over-constrained, profiling the various sources of theoretical uncertainty. For example, when including all eight measured distributions, the $|\eta ^{\ell }|$ and $|y^{e\mu }|$ distributions have little sensitivity to $m_t$, but constrain the PDF parameters. The $\Delta \phi ^{e\mu }$ distribution constrains the QCD scale parameters $\mu _F$ and $\mu _R$, under the assumption that uncertainties in higher-order QCD corrections are parameterised by $\mu _F$ and $\mu _R$ in a way that can be transported from one distribution to another. Two alternative dynamical scale choices were also tested in order to probe this assumption, as discussed in Sect. 8.5 below.

Potential biases in the method were checked by using predictions with ${m_t}=172.5$ GeV as pseudo-data, and considering both experimental and theoretical uncertainties in the $\chi ^2$ definition. The resulting fitted values of $m_t$ were within 0.1 GeV of the input value for all five fitted individual distributions ($p_{\mathrm T}^{\ell }$, $p_{\mathrm T}^{e\mu }$, $m^{e\mu }$, $p_{\mathrm T}^{e}+p_{\mathrm T}^{\mu }$ and $E^{e}+E^{\mu }$), and 0.01 GeV from the input value for a combined fit of all eight distributions, also including $|\eta ^{\ell }|$, $|y^{e\mu }|$ and $\Delta \phi ^{e\mu }$. The widths of the pull distributions were found to be compatible with unity, confirming the validity of the uncertainty estimates from the fits.

### Mass results from fixed-order predictions

**Fig. 19:**
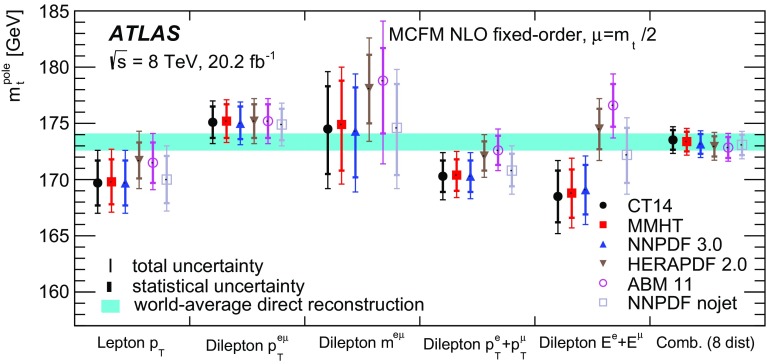
Measurements of the top quark mass using predictions derived from MCFM with the CT14, MMHT, NNPDF 3.0, HERAPDF 2.0, ABM 11 and NNPDF 3.0_nojet PDF sets. The central factorisation and renormalisation scales are set to $\mu _F=\mu _R={m_t}/2$. The results from fitting templates of the single lepton $p_{\mathrm T}^{\ell }$ and dilepton $p_{\mathrm T}^{e\mu }$, $m^{e\mu }$, $p_{\mathrm T}^{e}+p_{\mathrm T}^{\mu }$ and $E^{e}+E^{\mu }$ distributions one at a time, and of a combined fit to these five distributions plus the $|\eta ^{\ell }|$, $|y^{e\mu }|$ and $\Delta \phi ^{e\mu }$ distributions together, are shown. For comparison, the world-average of mass measurements from reconstruction of the top quark decay products and its uncertainty [121] is shown by the cyan band

The results of the fits to NLO QCD fixed-order predictions with MCFM and the CT14 PDF set are shown for the individual distributions in Table 15, and the results using the CT14, MMHT, NNPDF 3.0, HERAPDF 2.0, ABM 11 [123] and NNPDF 3.0_nojet [105] PDF sets are shown in Fig. 19. As shown in Sect. 7, the constraint on the gluon PDF from the leptonic $t\bar{t}$ measurements is consistent with the PDF determination from DIS data. The use of the NNPDF 3.0_nojet PDF set, which does not include Tevatron and LHC jet production data, allows the effects on $m_t$ of any possible tension between DIS and jet data in the determination of the gluon PDF to be tested. The results from combined fits to all eight distributions, using predictions from all six PDF sets, are shown in Table 16 and Fig. 19. In Tables 15 and 16, the decomposition of the total uncertainty from each mass fit into statistical, experimental and theoretical (PDF and QCD scales) uncertainties was obtained in analogy to the numerical procedure outlined in Ref. [124]. For each individual source of statistical or systematic uncertainty (corresponding to a nuisance parameter $b_{j,{\mathrm {exp}}}$ or $b_{k,{\mathrm {th}}}$ in Eq. (9)), the data were shifted by plus or minus one standard deviation, and a new $m_t$ value obtained by re-minimising the $\chi ^2$ function. The resulting shifts in $m_t$ were added in quadrature to obtain the decomposition into the various categories. The quadrature sum of the decomposed uncertainties agrees with the total to within 10% in all cases, the residual differences being due to non-linearity between the uncertainty sources and the extracted values of $m_t$.

**Table 15:** Measurements of the top quark mass from individual fits to the lepton $p_{\mathrm T}^{\ell }$ and dilepton $p_{\mathrm T}^{e\mu }$, $m^{e\mu }$, $p_{\mathrm T}^{e}+p_{\mathrm T}^{\mu }$ and $E^{e}+E^{\mu }$ distributions, using fixed-order predictions from MCFM with the CT14 PDF set. The $\chi ^2$ value at the best-fit mass for each distribution, the fitted mass with its total uncertainty, and the individual uncertainty contributions from data statistics, experimental systematics, and uncertainties in the predictions from PDF and QCD scale effects are shown

	$p_{\mathrm T}^{\ell }$	$p_{\mathrm T}^{e\mu }$	$m^{e\mu }$	$p_{\mathrm T}^{e}+p_{\mathrm T}^{\mu }$	$E^{e}+E^{\mu }$
$\chi ^2/N_{\mathrm dof}$	9/8	5/7	11/10	11/6	8/8
$m_t^{\mathrm {pole}}$ (GeV)	$169.7\,^{+2.9}_{-2.7}$	$175.1\pm 1.9$	$174.5\,^{+5.1}_{-5.3}$	$170.3\pm 2.1$	$168.5\,^{+3.2}_{-3.3}$
Data statistics	$\pm \,2.0$	$\pm \,1.4$	$\,^{+3.8}_{-4.0}$	$\pm \,1.4$	$\pm \,2.3$
Expt. systematic	$\,^{+2.5}_{-2.3}$	$\pm \,0.9$	$\,^{+2.9}_{-3.3}$	$\,^{+1.5}_{-1.6}$	$\pm \,2.0$
PDF uncertainty	$\pm \,0.5$	$\pm \,0.1$	$\pm \,1.1$	$\pm \,0.5$	$\pm \,1.4$
QCD scales	$\pm \,1.1$	$\,^{+0.7}_{-0.8}$	$\pm \,2.6$	$\,^{+0.4}_{-0.5}$	$\pm \,0.7$

**Table 16:** Measurements of the top quark mass from combined fits to all eight lepton and dilepton distributions, using fixed-order predictions from MCFM with the CT14, MMHT, NNPDF 3.0, HERAPDF 2.0, ABM 11 and NNPDF 3.0_nojet PDF sets, and various choices for the central QCD factorisation and renormalisation scales $\mu _F$ and $\mu _R$. The upper section of the table gives the results for $\mu _F=\mu _R={m_t}/2$, showing the $\chi ^2$ values at the best-fit mass for each PDF set, the fitted mass with its total uncertainty, and the breakdown of individual uncertainty contributions from data statistics, experimental systematics, and uncertainties in the predictions from PDF and QCD scale effects. Uncertainties given as ‘0.0’ are smaller than 0.05 GeV. The lower parts of the table give the $\chi ^2$ values, fitted mass and total uncertainty for alternative scale choices of $\mu _F=\mu _R=H_T/4$ and $E_T/2$

	CT14	MMHT	NNPDF 3.0	HERAPDF 2.0	ABM 11	NNPDF nojet
$$\mu _F=\mu _R={m_t}/2$$						
$\chi ^2/N_{{\mathrm {dof}}}$	71/68	70/68	67/68	67/68	71/68	64/68
$m_t^{\mathrm {pole}}$ (GeV)	$173.5\pm 1.2$	$173.4\pm 1.2$	$173.2\pm 1.2$	$172.9\pm 1.2$	$172.8\,^{+1.3}_{-1.2}$	$173.1\pm 1.2$
Data statistics	$\pm \,0.9$	$\pm \,0.9$	$\pm \,0.9$	$\pm \,0.9$	$\pm \,0.9$	$\pm \,0.9$
Expt. systematic	$\,^{+0.7}_{-0.8}$	$\pm \,0.8$	$\pm \,0.8$	$\pm \,0.9$	$\,^{+0.9}_{-0.8}$	$\pm \,0.8$
PDF uncertainty	$\pm \,0.1$	$\pm \,0.1$	$\,^{+0.1}_{-0.2}$	$\pm \,0.1$	$\pm \,0.1$	$\pm \,0.4$
QCD scales	$\pm \,0.1$	$\pm \,0.1$	$\,^{+0.1}_{-0.0}$	$\pm \,0.1$	$\pm \,0.1$	$\pm \,0.0$
$$\mu _F=\mu _R=H_T/4$$						
$\chi ^2/N_{\mathrm dof}$	69/68	67/68	64/68	61/68	66/68	60/68
$m_t^{\mathrm {pole}}$ (GeV)	$173.6\pm 1.3$	$173.4\pm 1.3$	$173.2\pm 1.3$	$173.6\pm 1.3$	$173.7\,^{+1.3}_{-1.2}$	$173.2\,^{+1.3}_{-1.4}$
$$\mu _F=\mu _R=E_T/2$$						
$\chi ^2/N_{{\mathrm {dof}}}$	71/68	70/68	66/68	64/68	68/68	64/68
$m_t^{\mathrm {pole}}$ (GeV)	$174.7\pm 1.4$	$174.5\,^{+1.5}_{-1.4}$	$174.3\,^{+1.5}_{-1.4}$	$173.6\,^{+1.3}_{-1.2}$	$173.4\,^{+1.2}_{-1.1}$	$174.0\,^{+1.5}_{-1.4}$

The MCFM fixed-order results for individual distributions shown in Table 15 and Fig. 19 show some similar patterns to those from the Powheg + Pythia6-based template fits shown in Table 14 and Fig. 17. The results from $p_{\mathrm T}^{\ell }$ and $p_{\mathrm T}^{e}+p_{\mathrm T}^{\mu }$ are close, the largest $m_t$ values come from $p_{\mathrm T}^{e\mu }$, the smallest from $E^{e}+E^{\mu }$ and the least precise determination is obtained from $m^{e\mu }$. The envelope of the central values is similar (6 GeV), but all values are shifted up by a few GeV compared to the corresponding Powheg + Pythia6-based template fit results for the same distribution. The $\chi ^2$ values are reasonable, indicating a satisfactory description of the data by the predictions at the best-fit $m_t$ values. The various distributions show different relative sensitivities to the PDF and QCD scale uncertainties.

As shown in Table 16, the combination of all eight measured distributions (including $|\eta ^{\ell }|$, $|y^{e\mu }|$ and $\Delta \phi ^{e\mu }$ which are not sensitive to $m_t$) significantly reduces the theoretical uncertainties due to both PDF and QCD scale effects. The $\chi ^2$ values for the combined description of all eight distributions are reasonable for all PDFs, implying that there is no significant tension between the mass fit results from the individual distributions, once the correlations between the distributions are taken into account. Several additional tests using the predictions based on NNPDF 3.0 were performed to probe the compatibility of the top quark mass values extracted from the different distributions, and the accuracy of the physics modelling used to perform the extraction. The combined fit was repeated removing one distribution at a time. The largest shift of $-1.4\pm 1.1$ GeV was observed when removing the $p_{\mathrm T}^{e\mu }$ distribution, where the uncertainty corresponds to the quadrature difference of the fit uncertainties with and without the $p_{\mathrm T}^{e\mu }$ distribution included. The removal of any other single distribution changed the result by less than 0.3 GeV, and a fit to only the five distributions directly sensitive to $m_t$ (excluding $|\eta ^{\ell }|$, $|y^{e\mu }|$ and $\Delta \phi ^{e\mu }$) gave a result of $173.1\pm 1.2$ GeV, corresponding to a shift of −0.1 GeV with respect to the eight-distribution result. Finally, the individual measurements from the five directly-sensitive distributions were combined using the HAverager program [125, 126]. Correlated statistical and systematic uncertainties were taken into account using nuisance parameters, but post-fit correlations between these nuisance parameters were neglected, unlike in the simultaneous fit approach with xFitter. The average of the five measurements is $173.4\pm 1.6$ GeV with a $\chi ^2$ of 6.4 / 4, in reasonable agreement with the result from the simultaneous fit of the five distributions. No additional uncertainty was included as a result of these tests.

The combined-fit $\chi ^2$ values in Table 16 are smallest for the HERAPDF 2.0 and NNPDF 3.0_nojet PDF sets, which do not include the constraints on the gluon PDF from LHC and Tevatron jet data in the region relevant for $t\bar{t}$ production. However, the $m_t$ values resulting from the NNPDF 3.0 and NNPDF 3.0_nojet PDFs are close, indicating that the results are not sensitive to whether the jet data are included or not. Amongst the ‘global fit’ PDF sets incorporating a larger set of experimental data, the smallest $\chi ^2$ values result from the fit with NNPDF 3.0, though the values from the other PDFs are also reasonable. The results using NNPDF 3.0 were therefore used to define the central $m_t$ value from the combined fit to all eight distributions, and an additional uncertainty of 0.3 GeV, corresponding to half the difference of the envelope encompassing all the other PDFs, was added in quadrature to the PDF uncertainty from NNPDF 3.0 alone. The effect of the uncertainty in the value of $\alpha _{\text {S}}$ was found to be 0.01 GeV. The residual dependence of the measured differential cross-sections on the top quark mass assumed in the simulation (see Sect. 5.1) is very small. A $\pm 5$ GeV variation around the baseline value of ${m_t}=172.5$ GeV was assumed, giving a 0.1 GeV change on the result of the combined fit.

The choice of a fixed central scale, $\mu _F=\mu _R={m_t}/2$ is expected to provide a good description of the inclusive $t\bar{t}$ cross-section and differential distributions in the kinematic regions dominated by top quarks with relatively low $p_{\text {T}}$. However, dynamical scales, which vary as a function of the top quark kinematics, are expected to be more appropriate for modelling the regions with high $p_{\text {T}}$ [107]. Two alternative dynamical central scale choices for the $t\bar{t}$ production process were explored to test the sensitivity of the results to this choice:

**Fig. 20:**
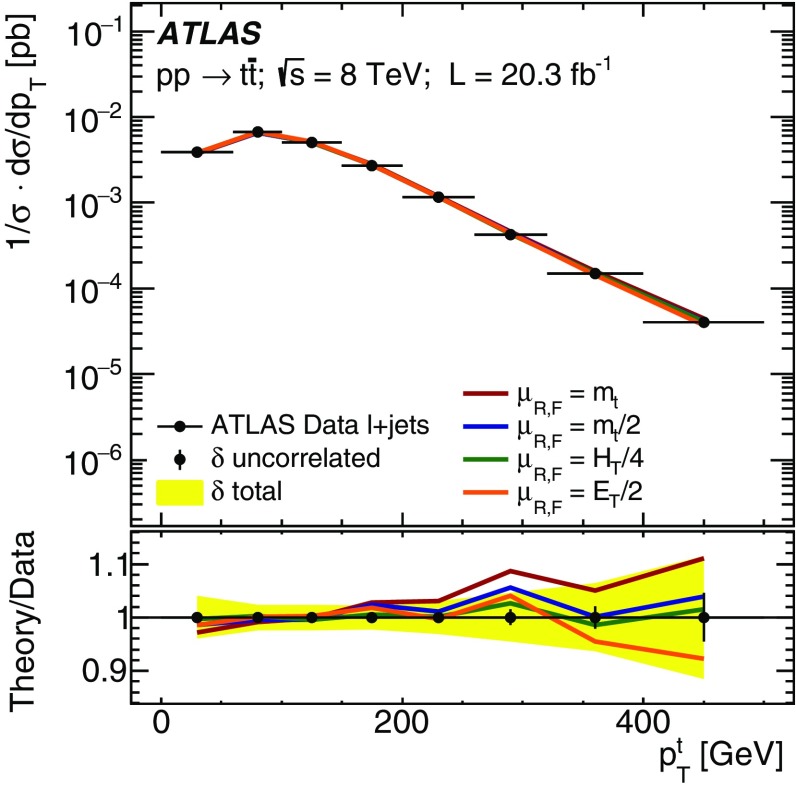
Measurement of the top quark $p_{\text {T}}$ spectrum in *pp* collisions at $\sqrt{s}=8$ TeV from ATLAS events with a lepton and at least four jets [20], compared to the predictions from MCFM as used in this analysis with NNPDF 3.0, ${m_t}=173.3$ GeV, and QCD scale choices of $\mu _F=\mu _R={m_t}/2$, $H_T/4$ and $E_T/2$, as well as with $\mu _F=\mu _R={m_t}$. The measurement uncertainties are represented by the yellow band, with the uncorrelated component shown by the black error bar. The lower plots show the ratios of the different predictions to the data

**Fig. 21:**
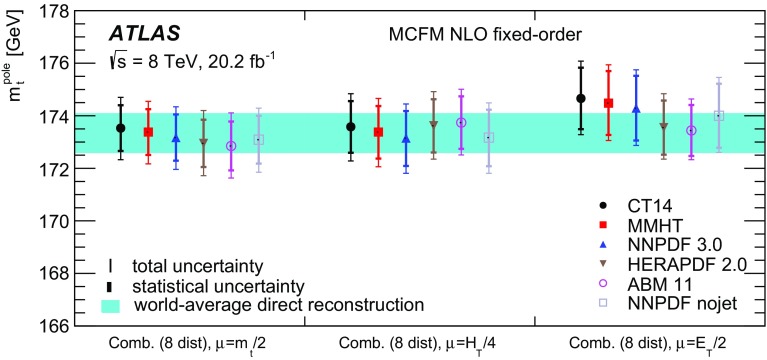
Measurements of the top quark mass using predictions derived from MCFM with the CT14, MMHT, NNPDF 3.0, HERAPDF 2.0, ABM 11 and NNPDF 3.0_nojet PDF sets, and the central QCD factorisation and renormalisation scales $\mu _F$ and $\mu _R$ set to ${m_t}/2$, $H_T/4$ and $E_T/2$. The results are derived from a combined fit to all eight lepton and dilepton distributions. For comparison, the world-average of mass measurements from reconstruction of the top quark decay products [121] is shown by the cyan band

- $\mu _F=\mu _R=H_T/4$ where $H_T$ is defined as $\sqrt{{m_t}^2+p_{\text {T}} (t)^2}+\sqrt{{m_t}^2+p_{\text {T}} (\bar{t})^2}$ and $p_{\text {T}} (t)$ and $p_{\text {T}} (\bar{t})$ are the transverse momentum of the top quark and antiquark, corresponding to one of the dynamical scales suggested in Ref. [107].
- $\mu _F=\mu _R=E_T/2$ where $E_T$ is defined as $\sqrt{{m_t}^2+p_{\text {T}} (t\bar{t})^2}$ and $p_{\text {T}} (t\bar{t})$ is the $p_{\text {T}}$ of the $t\bar{t}$ system, analogously to a scale $\sqrt{m_W^2+p_{\text {T}} (W)^2}$ used in the description of jet production in association with *W* bosons [127, 128].

In both cases, the central scale for the top quark decay process $t\rightarrow b\ell \nu +X$ was fixed at ${m_t}/2$. The corresponding predictions for the top quark $p_{\text {T}}$ spectrum from MCFM with NNPDF 3.0 and these scale choices are shown in Fig. 20, and compared to the ATLAS $\sqrt{s}=8$ TeV measurement using $t\bar{t}$ events with a lepton and at least four jets [20]. Unlike the predictions of Powheg + Pythia6 used in Sect. 8.3, the MCFM predictions with central scale choices of $\mu _F=\mu _R={m_t}/2$, $H_T/4$ and $E_T/2$ provide good descriptions of the measured top quark $p_{\text {T}}$ spectrum, whereas $\mu _F=\mu _R={m_t}$ is too hard.

The results from the combined fit to all eight distributions with these scale choices and all six PDF sets are shown in the lower part of Table 16, and displayed graphically in Fig. 21. In the same way as for the fixed central scale, the actual factorisation and normalisation scales used in the predictions were allowed to vary independently around the dynamical central scales, with one standard deviation variations corresponding to factors of two and one-half. The $\chi ^2$ values for the fits with a central scale of $H_T/4$ are all improved compared to those for ${m_t}/2$, reflecting a generally better description of the high-$p_{\text {T}}$ tails of the distributions. The $\chi ^2$ values from the $E_T/2$ fits lie between the other two choices. The largest difference in the $m_t$ values from a dynamical scale and the fixed scale with any PDF (1.1 GeV for $E_T/2$ vs. ${m_t}/2$ with the CT14 PDF) was used to define an additional theoretical uncertainty due to the choice of the functional form of the QCD scales.

**Fig. 22:**
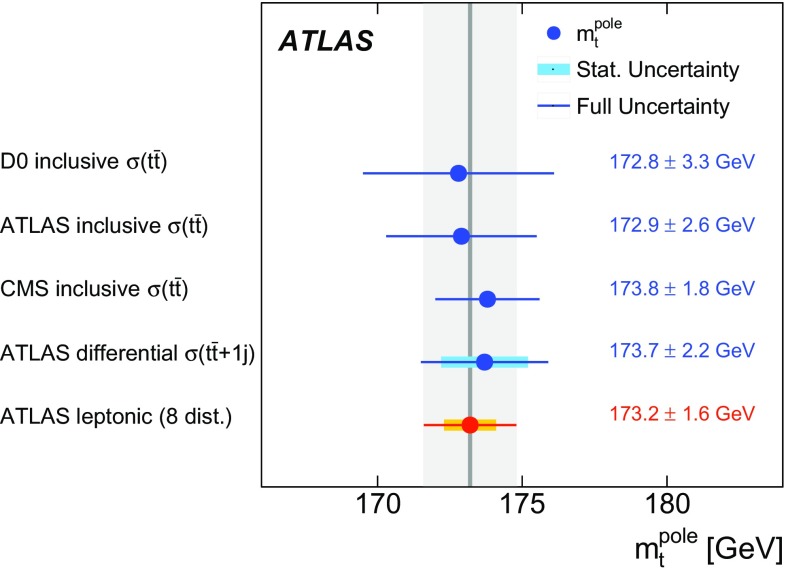
Result of the top quark pole mass determination from the combined fit to eight leptonic distributions (shown by the red point and grey band), compared to other determinations from inclusive and differential cross-section measurements in $t\bar{t}$ events [13, 15, 40, 41]. The statistical uncertainties are shown separately by the thick error bars where available

The final top quark mass value from the combination of all distributions is:

$$\begin{aligned} {m_t^{\mathrm {pole}}}=173.2\pm 0.9\pm 0.8\pm 1.2\,\mathrm{GeV}, \end{aligned}$$

where the three uncertainties arise from data statistics, experimental systematic effects, and uncertainties in the theoretical predictions, giving a total uncertainty of $1.6$ GeV. The theoretical uncertainty is dominated by the comparison of results with different QCD central scale choices. Figure 22 shows a comparison with previous determinations of the top quark pole mass from the inclusive $t\bar{t}$ production cross-section [13, 15, 40] and from the invariant mass distribution of the $t\bar{t}$ plus one jet system [41]. The present result is in agreement with these other results, all of which have larger uncertainties. It is also in agreement with the Tevatron and LHC average measurement of $173.34\pm 0.76$ GeV from reconstruction of the top quark decay products [121], as well as with more precise recent results using similar techniques [35, 36, 129]. However, the precision of the present pole mass result is not sufficient to probe potential differences between it and the other techniques at the 1 GeV level.

The theoretical uncertainty of 1.2 GeV on the final result using fixed-order predictions is significantly smaller than the uncertainties due to $t\bar{t}$ modelling and potential NNLO effects in the top quark $p_{\text {T}}$ spectrum for the fits based on Powheg + Pythia6 templates. In the fixed-order approach, the potential missing NNLO corrections are absorbed into the variations of the QCD scales $\mu _F$ and $\mu _R$, which are significantly constrained by the fit to the complete set of distributions, including those with little sensitivity to $m_t$. However, there remains a significant uncertainty of about 1 GeV due to the choice of the functional form of the QCD scales, limiting the gain from the combined fit. This approach would therefore benefit significantly from the availability of fixed-order calculations including NNLO effects in the top quark production and decay [130], which should reduce the uncertainties due to scale choices. Off-shell and interference effects in the $pp\rightarrow WWb\bar{b} \rightarrow e\mu \nu \bar{\nu }b\bar{b} +X$ process (including both $t\bar{t}$ and single top *Wt* contributions) [131–137], as well as NLO electroweak corrections [138, 139], were not considered in this analysis. They are expected to be small compared to the theoretical uncertainties of the current result, but likely cannot be neglected in a determination of $m_t$ based on NNLO QCD predictions. These theoretical advances would allow the power of the full set of distributions to be utilised more effectively, especially in view of the likely reduction in the experimental statistical and systematic uncertainties from the larger $t\bar{t}$ samples now becoming available from LHC running at $\sqrt{s}=13$ TeV.

## Conclusions

Lepton and dilepton differential cross-section distributions have been measured in $t\bar{t} \rightarrow e\mu \nu \bar{\nu }b\bar{b}$ events selected from 20.2 $\hbox {fb}^{-1}$of *pp* collisions at $\sqrt{s}=8$ TeV recorded by the ATLAS detector at the LHC. The absolute and normalised cross-sections were measured using opposite-charge $e\mu$ events with one or two *b*-tagged jets, and corrected to a fiducial volume corresponding to the experimental acceptance of the leptons and no requirements on jets. Eight single lepton and dilepton differential distributions were measured, with relative uncertainties varying in the range 1–10%, and presented with and without the contribution from leptonic decays of $\tau$-leptons produced in the *W* decays.

The results were compared to the predictions of various $t\bar{t}$ NLO and LO multileg matrix element event generators interfaced to several parton shower and hadronisation models. These generally give a good description of the distributions, though some distributions are modelled poorly by certain event generators. Those involving rapidity information are better described by the HERAPDF PDF sets than the CT10 set used as default. The distributions also show some sensitivity to NNLO corrections in the description of the top quark $p_{\text {T}}$ spectrum. The data are sensitive to the gluon PDF around $x\approx 0.1$ and have the potential to reduce PDF uncertainties in this region.

Several of the measured distributions are sensitive to the top quark mass, in a way which is complementary to traditional measurements of $m_t$ using the invariant mass of the reconstructed top quark decay products. Various techniques for extracting the top quark mass from the measured distributions were explored, including fits using templates from Powheg + Pythia6 simulated samples, mass determinations based on moments of the distributions, and fits to fixed-order NLO QCD predictions, giving access to the top quark pole mass in a well-defined renormalisation scheme as implemented in MCFM. The most precise result was obtained from a fit of fixed-order predictions to all eight measured distributions simultaneously, extracting $m_t^{\mathrm {pole}}$ whilst simultaneously constraining uncertainties due to PDFs and QCD scales. The final result is:

$$\begin{aligned} {m_t^{\mathrm {pole}}}=173.2\pm 0.9\pm 0.8\pm 1.2\,\mathrm{GeV}, \end{aligned}$$

where the three uncertainties arise from data statistics, experimental systematic effects, and uncertainties in the theoretical predictions. This result is in excellent agreement with other determinations of $m_t^{\mathrm {pole}}$ from inclusive and differential cross-sections, and traditional measurements based on reconstruction of the top quark decay products.

## Footnotes

1 Charge-conjugate decay modes are implied unless otherwise stated.

2 ATLAS uses a right-handed coordinate system with its origin at the nominal interaction point in the centre of the detector, and the *z* axis along the beam line. Pseudorapidity is defined in terms of the polar angle $\theta$ as $\eta =-\ln \tan {\theta /2}$, and transverse momentum and energy are defined relative to the beamline as $p_{\text {T}} =p\sin \theta$ and $E_{\text {T}} =E\sin \theta$. The azimuthal angle around the beam line is denoted by $\phi$, and distances in $(\eta ,\phi )$ space by $\Delta R=\sqrt{(\Delta \eta )^2+(\Delta \phi )^2}$. The rapidity is defined as $y=\frac{1}{2}\ln \left( \frac{E+p_z}{E-p_z}\right)$, where $p_z$ is the *z*-component of the momentum and *E* is the energy of the relevant object or system.

3 The reconstructed vertex with the largest sum of $p_{\text {T}} ^2$ for the constituent tracks was selected as the primary vertex.

4 The background event counts and uncertainties shown in Table 2 differ from those in Ref. [13] due to the use of different simulation samples and the estimation of the background in bins of lepton kinematic variables.

5 The integrals of the $p_{\mathrm T}^{\ell }$ and $|\eta ^{\ell }|$ distributions correspond to twice these values, as the definitions include two leptons per event.

6 Although HERAPDF 1.5 has been superseded by HERAPDF 2.0 [97], which uses the final combined DIS data from the H1 and ZEUS experiments, HERAPDF 1.5 is used here due to availability of the corresponding simulation sample.

7 The $\chi ^2$ value does not depend on the choice of which bin is removed.
